# A Machine Learning Model for Predicting a Major Response to Neoadjuvant Chemotherapy in Advanced Gastric Cancer

**DOI:** 10.3389/fonc.2021.675458

**Published:** 2021-06-01

**Authors:** Yonghe Chen, Kaikai Wei, Dan Liu, Jun Xiang, Gang Wang, Xiaochun Meng, Junsheng Peng

**Affiliations:** ^1^ Department of Gastrointestinal Surgery, The Sixth Affiliated Hospital, Sun Yat-sen University, Guangzhou, China; ^2^ Department of Radiology, The Sixth Affiliated Hospital, Sun Yat-sen University, Guangzhou, China; ^3^ Department of Laboratory Science, The Second Affiliated Hospital, Guangzhou University of Chinese Medicine, Guangzhou, China; ^4^ School of Public Health, Sun Yat-sen University, Shenzhen, China; ^5^ Guangdong Institute of Gastroenterology, Guangdong Provincial Key Laboratory of Colorectal and Pelvic Floor Diseases, Guangzhou, China

**Keywords:** advanced gastric cancer, neoadjuvant chemotherapy, radiomics, pathological response, machine learning

## Abstract

**Aims:**

To develop and validate a model for predicting major pathological response to neoadjuvant chemotherapy (NAC) in advanced gastric cancer (AGC) based on a machine learning algorithm.

**Method:**

A total of 221 patients who underwent NAC and radical gastrectomy between February 2013 and September 2020 were enrolled in this study. A total of 144 patients were assigned to the training cohort for model building, and 77 patients were assigned to the validation cohort. A major pathological response was defined as primary tumor regressing to ypT0 or T1. Radiomic features extracted from venous-phase computed tomography (CT) images were selected by machine learning algorithms to calculate a radscore. Together with other clinical variables selected by univariate analysis, the radscores were included in a binary logistic regression analysis to construct an integrated prediction model. The data obtained for the validation cohort were used to test the predictive accuracy of the model.

**Result:**

A total of 27.6% (61/221) patients achieved a major pathological response. Five features of 572 radiomic features were selected to calculate the radscores. The final established model incorporates adenocarcinoma differentiation and radscores. The model showed satisfactory predictive accuracy with a C-index of 0.763 and good fitting between the validation data and the model in the calibration curve.

**Conclusion:**

A prediction model incorporating adenocarcinoma differentiation and radscores was developed and validated. The model helps stratify patients according to their potential sensitivity to NAC and could serve as an individualized treatment strategy-making tool for AGC patients.

## Introduction

Gastric cancer is the fifth most common malignancy in the world and the third leading cause of cancer-related death (1). The majority of patients are diagnosed at an advanced stage with a poor prognosis (2). In recent years, neoadjuvant chemotherapy (NAC) plus subsequent radical gastrectomy has become a popular treatment modality for advanced gastric cancer (AGC). Some scholars stated that NAC could result in tumor downstaging and a higher curative resection rate and may eventually prolong survival for AGC patients (3, 4). Some other trials stated that NAC failed to offer any survival benefit (5, 6). Moreover, well-designed prospective RCTs are still lacking. Thus, the benefit and necessity of NAC remain controversial. Previous studies have found that the survival benefit of NAC vastly depends on the pathological response of the tumor. Those with a major pathological response and significant downstaging gained more survival benefit than others (7, 8). However, for those with a minor response, NAC offers no survival benefit but only toxicity and the risk of tumor progression during chemotherapy that may hinder surgical resection. Thus, to achieve personalized precision medicine, a pre-intervention prediction model to identify major responders and minor responders is needed.

Radiomics, a newly developed textural analysis method based on high-throughput extraction of quantitative imaging features within the tumor region (9), has shown potential as a noninvasive predictor for histological grade (10, 11), tumor stage (12), and prognosis (13) in gastric cancer. In certain cancers, radiomic features have been demonstrated to be an effective predictor for responses to anticancer therapy (14, 15). However, similar work for AGC patients is lacking.

Thus, we conducted this study to evaluate the predictive value of radiomic features for a major response to NAC in AGC patients, aiming to build a predictive model integrated with clinical and radiomic parameters and to provide a practical tool for developing individualized treatment strategies.

## Methods

### Study Population and Data Collection

This study was approved by the ethical committee of the Sixth Affiliated Hospital, Sun Yat-sen University. We reviewed the gastric cancer database of our institution and included patients according to the following criteria:

Inclusion criteria: (i) patients with histologically confirmed adenocarcinoma of the stomach or esophagogastric junction who received NAC and radical gastrectomy; (ii) patients who underwent abdominal multidetector computed tomography (CT) inspection before any intervention started; and (iii) tumor lesions that are assessable according to The Response Evaluation Criteria in Solid Tumors Version 1.1 (16).

The exclusion criteria were as follows: (i) patients who received preoperative radiotherapy, trastuzumab therapy, or immunotherapy as a part of neoadjuvant therapy; (ii) patients with indistinguishable tumor lesions on the CT images due to insufficient filling of the stomach during the CT inspection; and (iii) patients with insufficient data.

All available pre-intervention clinical information was retrieved from the database, including sex, age, body mass index (BMI), adenocarcinoma differentiation, and tumor staging information according to the staging system of the AJCC 8th edition (17), as listed in **Table 1**.

**Table 1:** Patients characteristic in the training and validation cohort.

Characteristic	Training cohort			p-value	Validation cohort			p-value
	All (n = 144)	Minor response (n = 107)	Major response (n = 37)		All (n = 77)	Minor response (n = 53)	Major response (n = 24)	
**Sex (%)**								
*Male*	106 (73.6)	77 (72.0)	29 (78.4)	0.584	54 (70.1)	36 (67.9)	18 (75.0)	0.719
*Female*	38 (26.4)	30 (28.0)	8 (21.6)		23 (29.9)	17 (32.1)	6 (25.0)	
**Age**	57.94 ± 9.35	57.59 ± 9.51	58.97 ± 8.91	0.439	56.04 ± 11.35	54.75 ± 11.97	58.88 ± 9.44	0.141
**Location (%)**								
*Upper*	52 (36.1)	36 (33.6)	16 (43.2)	0.273	28 (36.4)	21 (39.6)	7 (29.2)	0.654
*Middle*	27 (18.8)	24 (22.4)	3 (8.1)		12 (15.6)	9 (17.0)	3 (12.5)	
*Lower*	62 (43.1)	45 (42.1)	17 (45.9)		35 (45.5)	22 (41.5)	13 (54.2)	
*Whole*	3 (2.1)	2 (1.9)	1 (2.7)		2 (2.6)	1 (1.9)	1 (4.2)	
**Differentiation of adenocarcinoma (%)**								
*Well*	6 (4.2)	2 (1.9)	4 (10.8)	0.001	2 (2.6)	1 (1.9)	1 (4.2)	0.178
*Moderately*	63 (43.8)	40 (37.4)	23 (62.2)		25 (32.5)	14 (26.4)	11 (45.8)	
*Poorly*	75 (52.1)	65 (60.7)	10 (27.0)		50 (64.9)	38 (71.7)	12 (50.0)	
**Clinical T stage (%)**								
*T2*	3 (2.1)	2 (1.9)	1 (2.7)	0.609	2 (2.6)	2 (3.8)	0 (0.0)	0.593
*T3*	73 (50.7)	51 (47.7)	22 (59.5)		33 (42.9)	21 (39.6)	12 (50.0)	
*T4a*	55 (38.2)	44 (41.1)	11 (29.7)		32 (41.6)	22 (41.5)	10 (41.7)	
*T4b*	13 (9.0)	10 (9.3)	3 (8.1)		10 (13.0)	8 (15.1)	2 (8.3)	
**Clinical N stage (%)**								
*N0*	6 (4.2)	5 (4.7)	1 (2.7)	0.968	2 (2.6)	2 (3.8)	0 (0.0)	1
*N+*	138 (95.8)	102 (95.3)	36 (97.3)		75 (97.4)	51 (96.2)	24 (100.0)	
**Regimen (%)**								
*Doublet*	58 (40.3)	46 (43.0)	12 (32.4)	0.35	31 (40.3)	21 (39.6)	10 (41.7)	1
*Triplet*	86 (59.7)	61 (57.0)	25 (67.6)		46 (59.7)	32 (60.4)	14 (58.3)	
**Cycles**	4.00 [4.00, 4.00]	4.00 [3.00, 4.00]	4.00 [4.00, 5.00]	0.045	4.00 [4.00, 5.00]	4.00 [4.00, 5.00]	4.00 [4.00, 4.00]	0.748
**Resection (%)**								
*Distal gastrectomy*	63 (43.8)	45 (42.1)	18 (48.6)	0.614	33 (42.9)	20 (37.7)	13 (54.2)	0.271
*Total gastrectomy*	81 (56.2)	62 (57.9)	19 (51.4)		44 (57.1)	33 (62.3)	11 (45.8)	
**Laparoscopy surgery(%)**								
*No*	28 (19.4)	20 (18.7)	8 (21.6)	0.883	10 (13.0)	8 (15.1)	2 (8.3)	0.652
*Yes*	116 (80.6)	87 (81.3)	29 (78.4)		67 (87.0)	45 (84.9)	22 (91.7)	
**Multivisceral resection(%)**								
*No*	132 (91.7)	96 (89.7)	36 (97.3)	0.275	70 (90.9)	48 (90.6)	22 (91.7)	1
*Yes*	12 (8.3)	11 (10.3)	1 (2.7)		7 (9.1)	5 (9.4)	2 (8.3)	
**Pathological T stage (%)**								
*T0*	23 (16.0)	0 (0.0)	23 (62.2)	<0.001	12 (15.6)	0 (0.0)	12 (50.0)	<0.001
*T1*	14 (9.7)	0 (0.0)	14 (37.8)		12 (15.6)	0 (0.0)	12 (50.0)	
*T2*	15 (10.4)	15 (14.0)	0 (0.0)		9 (11.7)	9 (17.0)	0 (0.0)	
*T3*	86 (59.7)	86 (80.4)	0 (0.0)		39 (50.6)	39 (73.6)	0 (0.0)	
*T4*	6 (4.2)	6 (5.6)	0 (0.0)		5 (6.5)	5 (9.4)	0 (0.0)	
**Pathological N stage (%)**								
*N0*	67 (46.5)	41 (38.3)	26 (70.3)	0.01	45 (58.4)	25 (47.2)	20 (83.3)	0.02
*N1*	31 (21.5)	24 (22.4)	7 (18.9)		9 (11.7)	6 (11.3)	3 (12.5)	
*N2*	24 (16.7)	22 (20.6)	2 (5.4)		12 (15.6)	11 (20.8)	1 (4.2)	
*N3a*	19 (13.2)	17 (15.9)	2 (5.4)		10 (13.0)	10 (18.9)	0 (0.0)	
*N3b*	3 (2.1)	3 (2.8)	0 (0.0)		1 (1.3)	1 (1.9)	0 (0.0)	
**Harvested Lymph Node**	29 ± 12	29 ± 12	27 ± 13	0.286	27 ± 12	27 ± 12	28 ± 12	0.842
**Radscore**	0.11 [-0.76, 0.86]	-0.04 [-0.92, 0.64]	1.05 [-0.29, 1.66]	<0.001	0.40 [-0.99, 1.01]	0.04 [-1.18, 0.58]	1.04 [0.33, 1.33]	0.001

### CT Image Acquisition, Retrieval Procedure, Radiomics Feature Extraction Methodology, and Determination of Pathological Response

**Figure 1:**
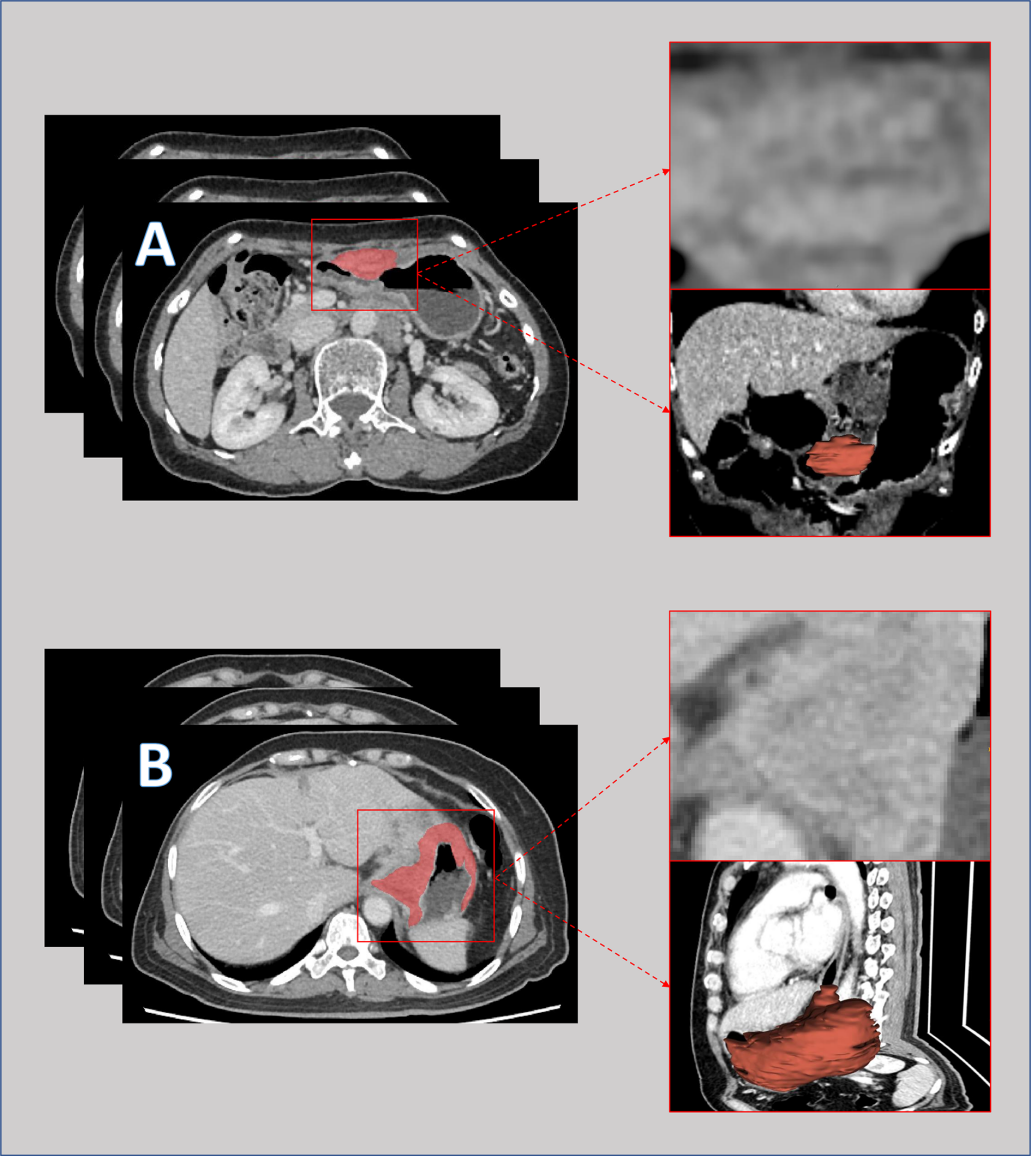
Pre-intervention venous-phase computed tomography images of a patient with major response **(A)** and a patient with minor response **(B)** to neoadjuvant chemotherapy. The lesions were delineated slice by slice and merged into a 3-dimensional region for features extraction.

The workflow of this study is depicted in **Supplementary Material S1**. Venous-phase contrast-enhanced abdominal CT images were retrieved from the picture archiving and communication system (details described in **Supplementary Material S2**). The region of interest (ROI) was delineated at each cross section of the primary tumor lesions by two senior licensed radiologists. Delineations were strictly confined within the tumor border using the segmentation tool ITK SNAP (18) ver. 3.6.0 (University of Pennsylvania, PA, USA). An example of CT image delineation was shown in **Figure 1**. Radiomic features of the ROI were extracted using the ‘pyradiomics’ package (19) in the Python programming language ver. 3.7.0 (Python Software Foundation, Virginia, USA; www.python.org). The list of extracted features is depicted in **Supplementary Materials S3 **and** S4**.

For pathological response assessment, all resection specimens were examined by two senior pathologists. A major response was defined as primary tumor regressing to ypT0 (absence of residual cancer cells in the primary tumor) or yp T1 (scattered cancer cells in the mucosa layer). The other cases were defined as a minor response.

### Statistical Analysis

All statistical analyses were performed by R software version 3.6.1 (The R Foundation for Statistical Computing, Vienna, Austria; www.r-project.org). Details of the machine learning algorithm and packages utilized are described in **Supplementary Material S5**. P-values<0.05 were identified as statistically significant.

### Features Selection and Radscore Calculation

Clinical feature selection: Pre-intervention clinical characteristics that were significantly correlated with pathological response were selected.

Radiomic features were selected in 4 steps: In step 1, all radiomic features values were standardized according to the distance to mean value. In step 2, the correlations between the radiomic features and pathological response were tested by univariate analysis, and features with a P-value<0.05 were selected. In step 3, the machine learning algorithm of the least absolute shrinkage and selection operator (LASSO) method was used to reduce data dimensionalities, and features with a nonzero coefficient were further selected. In step 4, the radscore was calculated by linearly combining the coefficients of features from the third step.

### Development of an Individualized Prediction Model Integrating Clinical and Radiomic Features

After an individualized radscore was calculated for each patient, the total sample was randomized into a training cohort and a validation cohort. In the training cohort, the correlation between radscores and pathological responses was tested by univariate analysis. The selected clinical features and radscore are added to a multivariate binary logistic regression model. An individualized model integrating clinical features and radscore is established based on data obtained from the training cohort, visualizing the weights of each parameter in the model.

### Validation of the Integrated Model and Decision Curve Analysis

The data obtained from the validation cohort were used to test the prediction precision of the model. A calibration curve was plotted to assess the calibration between the model and the validation data set. The receiver’s operative curve (ROC) and the respective area under the curve (AUC) were used to test the discriminative power. Decision curve analysis was conducted to determine the predictive value of the integrated model compared to the prediction model based on the clinical characteristics or radiomic features alone.

## Results

### Patients Characteristic

From February 2013 to September 2020, 221 patients who received NAC and D2 radical gastrectomy were enrolled in the study. Patient characteristics in the training and validation cohorts are depicted in **Table 1**. The majority of patients were male (72.4%, 160/221), and the lesions were mostly poorly differentiated adenocarcinoma (56.6%, 125/221) with a clinical stage of T3-T4 (97.7%, 216/221) and radiologically suspicious lymph node metastasis (96.4%, 213/221). Cases were randomly assigned to a training cohort (n=144) for prediction model construction and a validation cohort (n=77) for model validation according to a preset 2:1 ratio. The demographic characteristics were similar in both cohorts, as shown in **Table 1**.

### Neoadjuvant Chemotherapy and Pathological Findings

Enrolled patients received a median of 4 cycles of NAC. Triplet agent regimens were the mainstream regimen (59.7%, 132/221). Most lesions were resected through laparoscopy (82.8%, 183/221). In the final pathological analysis, a total of 61 patients (27.6%) achieved a major response, of whom 35 regressed to ypT0 (15.8%) and 26 regressed to ypT1 (11.8%).

### Feature Selection and Radscore Calculation

**Figure 2:**
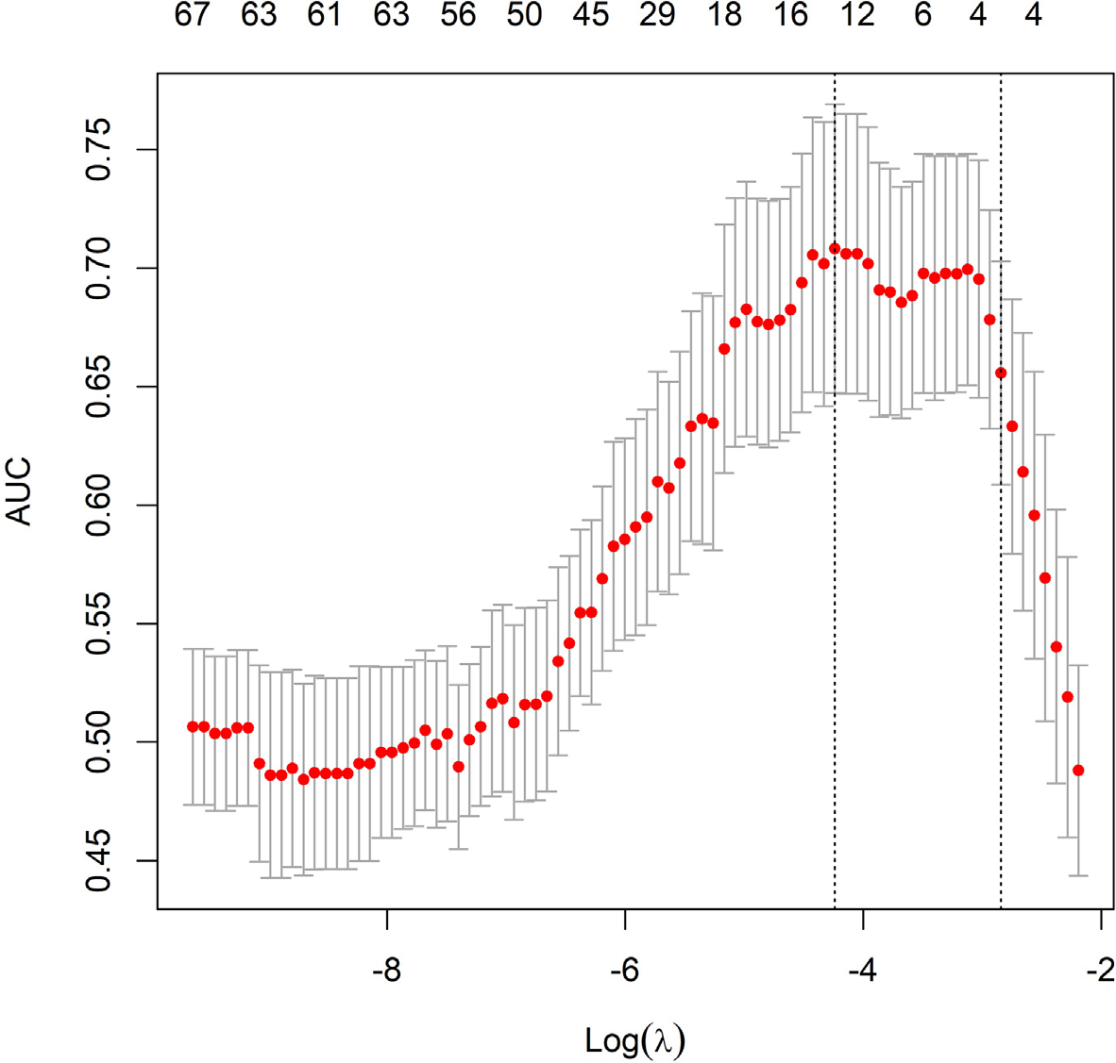
Radiomic feature selection using the least absolute shrinkage and selection operator (LASSO) model. The area under the receiver operating characteristic (ROC) curve was plotted versus the logarithm of tuning parameter λ. Dotted vertical lines were drawn at the optimal values using the minimum criteria and the 1 standard error of the minimum criteria (the 1-SE criteria).

**Figure 3:**
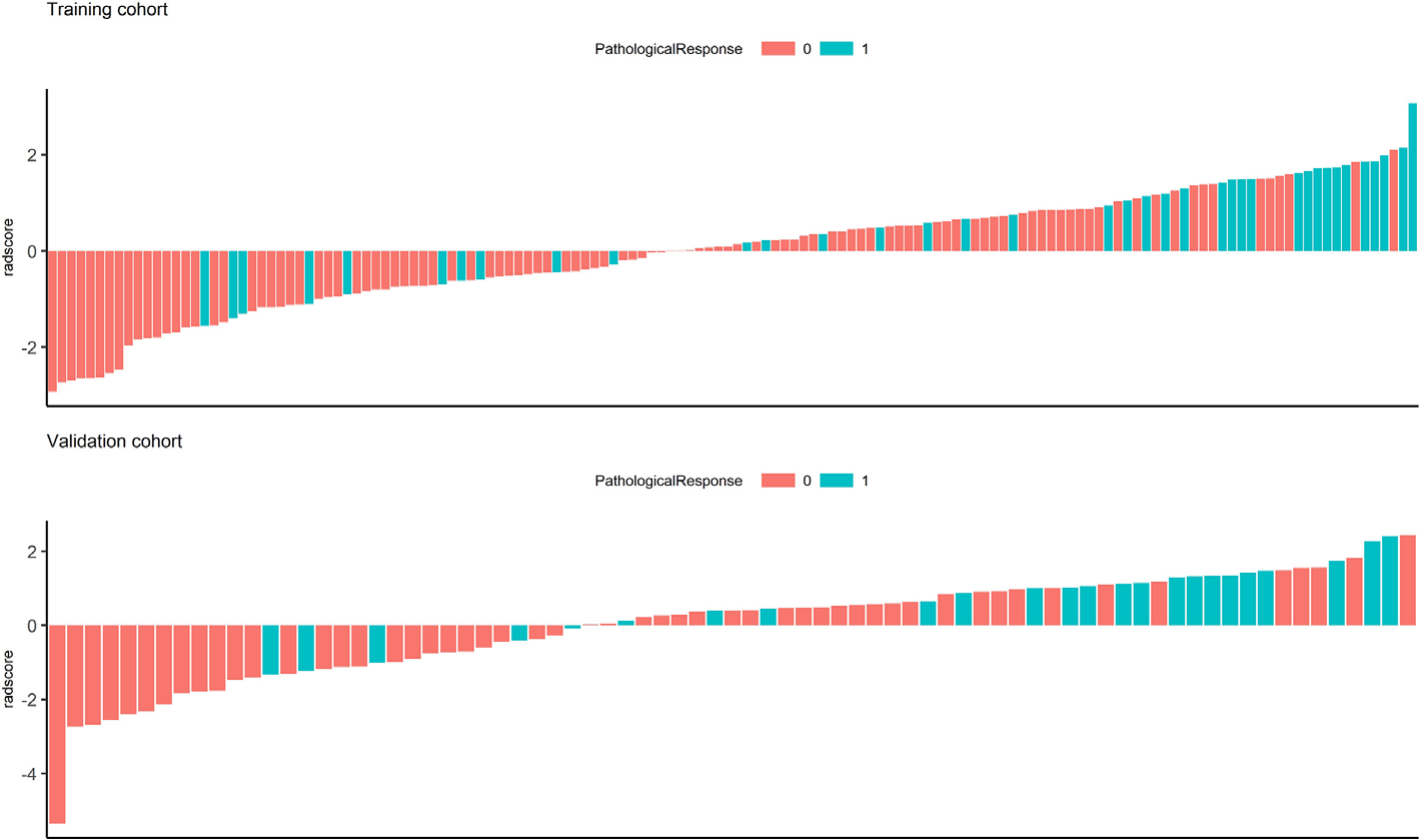
Waterfall chart showing radscores for each patient in the training and validation cohorts. The red columns represent patients with minor pathological responses, and the green columns represent those with major pathological responses.

In the univariate analysis, 92 of 572 features were selected according to the P-value (<0.05). In the binary LASSO regression, which is depicted in **Figure 2**, 5 features with nonzero coefficients were included in the radscore calculation formula (**Supplementary Material S6**). The distribution of radscore and responses to NAC is depicted in **Figure 3**.

### Development of a Prediction Model Integrating Clinical and Radiomic Parameters

**Figure 4:**
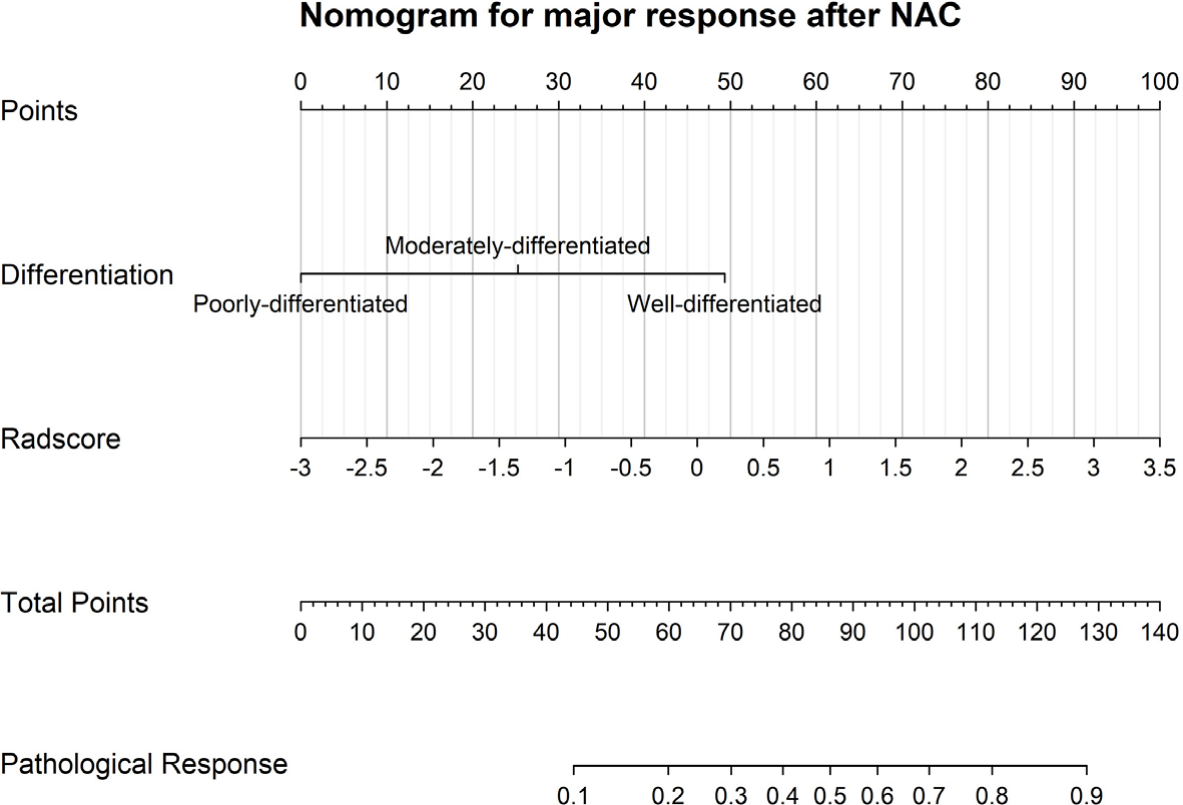
A visualized model for predicting major pathological response after neoadjuvant chemotherapy incorporating only pre-intervention characteristics, such as adenocarcinoma differentiation and CT radscores.

Among all the pre-intervention characteristics of the training cohort listed in **Table 1**, only adenocarcinoma differentiation and radscores were significantly correlated with major pathological response. Thus, these two factors were included in the binary logistic regression analysis. Based on their weight in the model, a model integrating clinical and radiomic parameters for predicting major response after NAC was constructed (**Figure 4**) with the radscore yielding the heaviest weight in the prediction model.

### Validation of the Integrated Model

**Figure 5:**
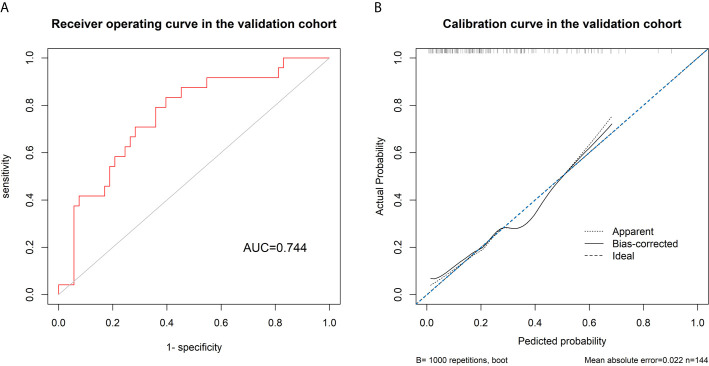
**(A)** Receiver’s operating curve for validating the discriminative power of the model using data in the validation cohort, showing a satisfactory discriminative power of the model with an area under the curve of 0.744. **(B)** The calibration curve shows a good fit between the data of the validation cohort and the model with a C-index of 0.763.

**Figure 6:**
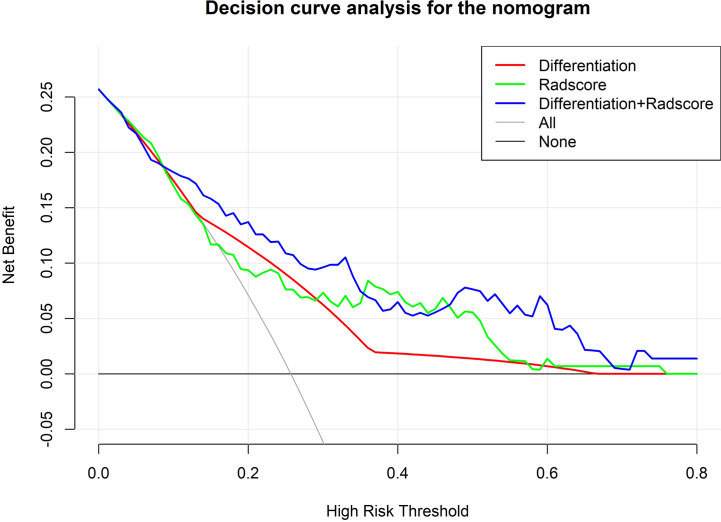
Decision curve analysis comparing the predictive value of different models. The Y-axis measures the net benefits. The X-axis represents the threshold probability for “positive” (indicating the patient is likely to achieve a major response after NAC and should be recommended for NAC). The green line represents predictions based on only radscores. The red line represents predictions based on only adenocarcinoma differentiation. The purple line represents predictions based on the model incorporating both radscores and differentiation. As shown in the figure, in most thresholds, the integrated model demonstrates superiority and more net benefit gains.

The AUC of the ROC curve of the model based on the data of the validation cohort was 0.744, showing satisfactory predictive discriminative power (**Figure 5A**). The calibration curve of the integrated model for the probability of a major response demonstrated satisfactory agreement between the training and validation cohorts (**Figure 5B**). The C-index based on the validation cohort for the training model was 0.763 (95% CI: 0.648-0.878), suggesting a good model fit. The result of the decision curve analysis is presented in **Figure 6**. We compared the predictive power of models including only the clinical parameter (adenocarcinoma differentiation) or radiomic parameters (radscore) to the model integrating both factors. The results confirmed the superiority of the integrated model, indicating that adenocarcinoma differentiation and radiomic features have an intercrossing incremental effect on each other, adding up to a more satisfactory prediction model for major responses to NAC.

## Discussion

In this study, we managed to develop and validate a model for predicting major response to NAC in AGC patients based on a machine learning approach. This model incorporates only pre-intervention clinical and CT radiomic features and effectively stratifies patients according to their sensitivity to NAC, making it a simple and practical tool for assisting individualized treatment strategy development.

In the model, the radscore represents the pre-intervention CT characteristics of each patient. The radscore was calculated in 3 steps. In the first step of univariate analysis, features without significant correction to major response were eliminated, and 92 features of 572 features were selected. In the second step, a machine learning algorithm, LASSO regression, was utilized, and features with collinearity and weak predictive strength were further eliminated, leaving only 5 features. In the third step, the remaining 5 features with the strongest independent predictive value were fit into a single radscore *via* linear combination weighted by coefficients. This approach was proven to be stable and effective and has been embraced by similar previous studies (20–23). Additionally, in the ROI delineation procedure, we adopted the 3-dimensional delineation method, which means that each cross section of the tumor was included and rebuilt into a 3-dimensional model. Previous research has indicated that this approach provides extracted features that are more stable, precise and reflect more detailed information on the tumor nature compared with the 2-dimensional delineation method (24). The radscore also retains a heavier weight in the final established prediction model, indicating satisfactory prediction power.

In the final established model, not only radiomic features but also clinical features were integrated. Among all the clinical features analyzed, only adenocarcinoma differentiation and cycles of NAC achieved statistical significance. Given that cycles of NAC were not a pre-intervention parameter, only differentiation was included. A higher differentiation grade was associated with a poorer response to chemotherapy, which is consistent with previous reports (25, 26).

For the choice of the outcome variable, we defined primary tumor regressing to ypT0 or T1 as a major response to NAC, as it is the definition used in early gastric cancer (27). Other previous reports also stated that the regression of the T stage is an important survival predictor, and patients with lower ypT stage are associated with more survival benefit gain from NAC (28–30). Thus, this variable can be used as an effective surrogate endpoint for survival (31).

Validation of the model showed a good fit between the validation cohort and the model. A c-index of 0.763 indicates robust predictive power. Decision curve analysis showed that by integrating radiomics and differentiation into the model, the prediction accuracy was higher than the prediction based on radscore or differentiation alone, indicating an intercrossing incremental value and further demonstrating the superiority of the integrated model. The model could serve as a useful reference tool for developing treatment strategies for AGC patients, especially since NAC has yet to become the standard approach for AGC. First, stratifying patients according to the probability of achieving a major response could not only help us identify patients with good sensitivity to NAC but also help patients with poor sensitivity to NAC avoid unnecessary toxicity and the risk of tumor progression. Second, the features included in our model were all easily achievable by pre-intervention routine inspection, with easily accessible tools and no excessive trauma to the patients.

A few limitations to our study should be noted. First, there was a lack of genomic data, such as microsatellite stability status, which are potential chemosensitivity predictors according to previous literature (32). Second, there was a lack of a prospective validation cohort from an independent institution to prove the model’s universality. Nevertheless, the image sets analyzed in our study were retrieved from CT scanners of various manufacturers, and the total sample was randomly divided into a training and a validation cohort based on a reasonable ratio. The final established model should be reliable and robust.

## Conclusion

In conclusion, a model integrating pre-intervention clinical and CT features for predicting major response to NAC was successfully developed and validated. The model helps stratify AGC patients according to their potential chemosensitivity and can serve as a practical tool for the development of individualized treatment strategies for advanced gastric cancer patients.

## Data Availability Statement

The raw data supporting the conclusions of this article will be made available by the authors, without undue reservation.

## Ethics Statement

The study was reviewed and approved by the ethics committee of The Sixth Affiliated Hospital, Sun Yat-Sen University. This study was conducted in accordance with the 1964 Helsinki Declaration.

## Author Contributions

JP, XM, and YC designed the study. YC, KW, and DL contributed equally to acquiring, analyzing, interpreting the data, and drafting the initial manuscript. GW performed the data analysis, and JX made important revisions to the manuscript. YC, KW, and DL contributed equally to this work. All authors contributed to the article and approved the submitted version.

## Funding

This study is supported by the Science and Technology Planning Project of Guangdong Province, China (grant number 2017A010105004), Research Fund of the Sixth Affiliated Hospital of Sun Yat-sen University (grant number P20200217202309876), and National Key Clinical Discipline.

## Conflict of Interest

The authors declare that the research was conducted in the absence of any commercial or financial relationships that could be construed as a potential conflict of interest.
