# Supplementary material for: A Machine Learning Model for Predicting a Major Response to Neoadjuvant Chemotherapy in Advanced Gastric Cancer
Source: Front Oncol. 2021 Jun 1;11:675458. doi: 10.3389/fonc.2021.675458 (PMC8204104; doi:10.3389/fonc.2021.675458)
Supplement: Supplementary file 1 [file DataSheet_1.docx]

***The workflow of regions of interest delineation, radiomic features extraction, selection, and predictive model building.***

***
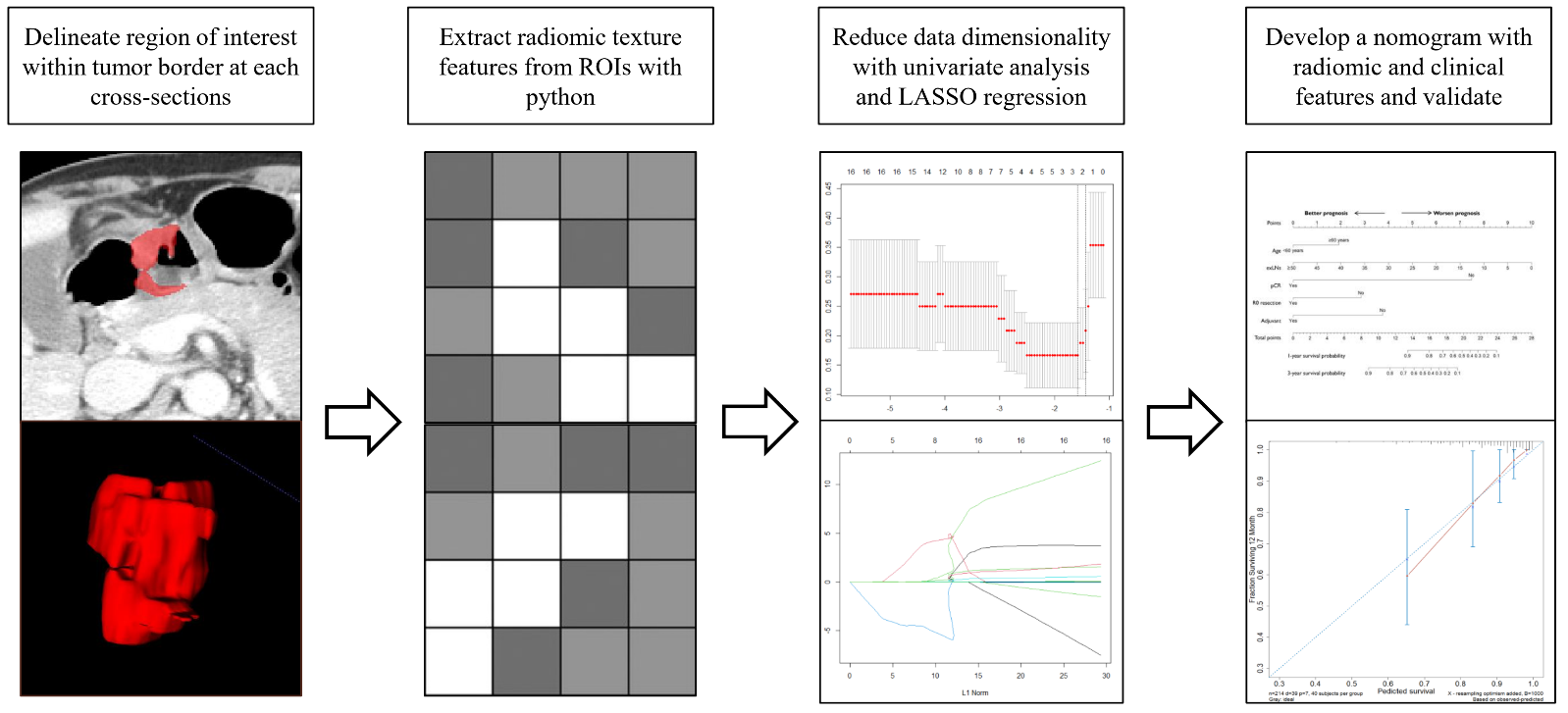
***
