# Supplementary material for: A Machine Learning Model for Predicting a Major Response to Neoadjuvant Chemotherapy in Advanced Gastric Cancer
Source: Front Oncol. 2021 Jun 1;11:675458. doi: 10.3389/fonc.2021.675458 (PMC8204104; doi:10.3389/fonc.2021.675458)
Supplement: Supplementary file 2 [file DataSheet_2.docx]

***CT Image Acquisition and Retrieval Procedure***

All patients underwent contrast-enhanced abdominal computed tomography (CT) with multidetector CT, and the acquisition parameters were as follows:

acquisition devices (GE optima 660 128-slice CT, TOSHIBA Aquilion One 320-slice CT); helical scan; 0.6-0.9, helical pitch; 120 Kv, auto mA; 0.6 second rotation time; field of view, 32 cm×32 cm-35 cm×35 cm; adaptive statistical iterative reconstruction (ASIR) technology. Arterial and venous phases were performed 28 and 60 s after a pre-noncontrast scan with venous injection of iodinated contrast material (Ultravist 370, Bayer Schering Pharma, Berlin, Germany; Omnipaque 300, Ge Healthcare Shanghai, China) at a rate of 3.0 or 3.5 ml/s with a pump injector. Contrast-enhanced CT was reconstructed with a reconstruction thickness of 3 mm or 3.75 mm. Venous phase CT images were saved from the picture archiving and comnumication system (PACS) for feature extraction in a digital imaging and communications in medicine (DICOM) format.
