# Supplementary material for: A Machine Learning Model for Predicting a Major Response to Neoadjuvant Chemotherapy in Advanced Gastric Cancer
Source: Front Oncol. 2021 Jun 1;11:675458. doi: 10.3389/fonc.2021.675458 (PMC8204104; doi:10.3389/fonc.2021.675458)
Supplement: Supplementary file 3 [file DataSheet_3.docx]

***Radiomic features of the minor and major response group in the training cohort*** ***and results of univariate analysis***

|  | Overall  (n=144) | Minor response  (n=107) | Major response  (n=37) | p |
| --- | --- | --- | --- | --- |
| original_shape_Elongation (median [IQR]) | 0.70[0.62,0.80] | 0.70[0.62,0.80] | 0.71[0.62,0.79] | 0.914 |
| original_shape_Flatness (median [IQR]) | 0.48[0.40,0.57] | 0.48[0.40,0.56] | 0.52[0.34,0.61] | 0.617 |
| original_shape_LeastAxisLength (median [IQR]) | 32.65 [24.92, 43.43] | 35.48 [27.21, 44.28] | 26.36 [18.73, 38.57] | <0.001 |
| original_shape_MajorAxisLength (median [IQR]) | 68.81[53.79,93.14] | 72.95[58.44,98.39] | 62.60[46.14,73.72] | 0.006 |
| original_shape_Maximum2DDiameterColumn (median [IQR]) | 73.88[54.49,96.58] | 75.67[60.75,101.16] | 58.19[45.81,82.53] | 0.008 |
| original_shape_Maximum2DDiameterRow (median [IQR]) | 67.56[53.55,89.64] | 70.77[57.99,94.99] | 58.22[44.21,71.73] | 0.003 |
| original_shape_Maximum2DDiameterSlice (median [IQR]) | 83.06[60.61,100.85] | 84.72[64.31,105.17] | 63.63[48.09,90.38] | 0.006 |
| original_shape_Maximum3DDiameter (median [IQR]) | 86.73 [65.87, 109.99] | 91.28 [72.51, 117.54] | 68.23 [54.11, 86.30] | <0.001 |
| original_shape_MeshVolume (median [IQR]) | 41253.42[20341.64,83307.93] | 48932.48[25941.55,88511.62] | 27031.44[11560.72,53149.38] | 0.005 |
| original_shape_MinorAxisLength (median [IQR]) | 51.47[38.91,62.97] | 53.07[41.71,64.55] | 42.39[30.58,55.90] | 0.007 |
| original_shape_Sphericity (median [IQR]) | 0.40[0.34,0.46] | 0.39[0.34,0.44] | 0.42[0.36,0.49] | 0.072 |
| original_shape_SurfaceArea (median [IQR]) | 15032.70[8602.52,25517.53] | 15940.91[10128.07,27053.96] | 9291.19[5313.70,19580.80] | 0.007 |
| original_shape_SurfaceVolumeRatio (median [IQR]) | 0.36[0.31,0.44] | 0.35[0.30,0.42] | 0.39[0.34,0.46] | 0.02 |
| original_shape_VoxelVolume (median [IQR]) | 41489.35[20459.26,83706.86] | 49129.02[26176.68,88820.60] | 27186.33[11678.57,53461.36] | 0.005 |
| original_firstorder_10Percentile (median [IQR]) | 53.00[43.00,63.00] | 55.00[44.00,63.00] | 48.00[39.00,57.00] | 0.038 |
| original_firstorder_90Percentile (median [IQR]) | 105.00[94.75,121.00] | 107.00[99.00,121.00] | 96.00[88.00,112.00] | 0.004 |
| original_firstorder_Energy (median [IQR]) | 146044582.50[72655263.00,283625532.00] | 174488595.00[100823912.00,313403376.00] | 85356783.00[33140689.00,170551769.00] | <0.001 |
| original_firstorder_Entropy (median [IQR]) | 1.92[1.73,2.06] | 1.93[1.74,2.06] | 1.90[1.61,2.02] | 0.172 |
| original_firstorder_InterquartileRange (median [IQR]) | 29.00[23.00,32.00] | 29.00[24.00,32.00] | 27.00[22.00,32.00] | 0.268 |
| original_firstorder_Kurtosis (median [IQR]) | 4.03[3.46,5.20] | 4.12[3.48,5.25] | 3.86[3.41,4.79] | 0.486 |
| original_firstorder_Maximum (median [IQR]) | 164.00[150.00,183.25] | 166.00[153.00,186.50] | 157.00[137.00,176.00] | 0.033 |
| original_firstorder_MeanAbsoluteDeviation (median [IQR]) | 17.34[14.81,19.43] | 17.36[15.01,19.43] | 16.86[13.40,18.98] | 0.198 |
| original_firstorder_Mean (median [IQR]) | 81.05[68.55,91.36] | 82.17[72.92,93.83] | 71.58[66.06,86.97] | 0.009 |
| original_firstorder_Median (median [IQR]) | 83.00[70.75,94.00] | 84.00[73.50,96.00] | 74.00[66.00,89.00] | 0.008 |
| original_firstorder_Minimum (median [IQR]) | -66.50[-121.25,-35.00] | -66.00[-129.50,-34.50] | -68.00[-100.00,-36.00] | 0.646 |
| original_firstorder_Range (median [IQR]) | 243.50[185.00,308.25] | 249.00[187.50,325.00] | 240.00[180.00,259.00] | 0.16 |
| original_firstorder_RobustMeanAbsoluteDeviation (median [IQR]) | 12.08[10.03,13.59] | 12.12[10.12,13.51] | 11.66[9.36,13.58] | 0.276 |
| original_firstorder_RootMeanSquared (median [IQR]) | 83.34 [72.45, 95.55] | 84.58 [76.74, 96.99] | 74.39 [69.35, 88.80] | 0.003 |
| original_firstorder_Skewness (median [IQR]) | -0.45[-0.71,-0.17] | -0.50[-0.73,-0.18] | -0.38[-0.59,-0.06] | 0.183 |
| original_firstorder_TotalEnergy (median [IQR]) | 309569582.30[143783317.21,600108712.44] | 364808894.98[192091294.28,652335759.58] | 189082186.38[69671057.50,360400635.38] | <0.001 |
| original_firstorder_Uniformity (median [IQR]) | 0.31[0.28,0.36] | 0.31[0.28,0.36] | 0.32[0.28,0.38] | 0.186 |
| original_firstorder_Variance (median [IQR]) | 482.70[366.18,625.40] | 490.46[376.46,627.96] | 470.55[291.52,565.60] | 0.148 |
| original_glcm_Autocorrelation (median [IQR]) | 49.88[31.32,85.63] | 52.76[32.58,94.48] | 44.90[28.81,66.72] | 0.093 |
| original_glcm_ClusterProminence (median [IQR]) | 19.17[11.88,33.62] | 19.51[13.32,34.01] | 16.59[8.61,32.83] | 0.097 |
| original_glcm_ClusterShade (median [IQR]) | -0.71[-1.53,-0.12] | -0.81[-1.82,-0.15] | -0.42[-1.27,0.17] | 0.085 |
| original_glcm_ClusterTendency (median [IQR]) | 2.31[1.76,3.02] | 2.41[1.83,3.04] | 2.17[1.35,2.76] | 0.109 |
| original_glcm_Contrast (median [IQR]) | 0.83[0.67,0.98] | 0.82[0.69,0.99] | 0.85[0.62,0.97] | 0.491 |
| original_glcm_Correlation (median [IQR]) | 0.47[0.40,0.55] | 0.48[0.42,0.55] | 0.44[0.33,0.55] | 0.099 |
| original_glcm_DifferenceAverage (median [IQR]) | 0.60[0.53,0.67] | 0.60[0.54,0.67] | 0.62[0.52,0.67] | 0.63 |
| original_glcm_DifferenceEntropy (median [IQR]) | 1.34[1.25,1.42] | 1.33[1.25,1.42] | 1.35[1.20,1.41] | 0.551 |
| original_glcm_DifferenceVariance (median [IQR]) | 0.43[0.38,0.50] | 0.43[0.39,0.50] | 0.44[0.35,0.49] | 0.357 |
| original_glcm_Id (median [IQR]) | 0.73[0.71,0.75] | 0.73[0.71,0.75] | 0.72[0.70,0.76] | 0.699 |
| original_glcm_Idm (median [IQR]) | 0.72[0.70,0.75] | 0.72[0.70,0.74] | 0.71[0.69,0.75] | 0.686 |
| original_glcm_Idmn (median [IQR]) | 0.99[0.99,1.00] | 0.99[0.99,1.00] | 0.99[0.99,0.99] | 0.208 |
| original_glcm_Idn (median [IQR]) | 0.95[0.94,0.96] | 0.95[0.94,0.96] | 0.95[0.93,0.96] | 0.234 |
| original_glcm_Imc1 (median [IQR]) | -0.13[-0.16,-0.10] | -0.14[-0.16,-0.10] | -0.12[-0.16,-0.08] | 0.143 |
| original_glcm_Imc2 (median [IQR]) | 0.56[0.47,0.64] | 0.57[0.50,0.65] | 0.51[0.42,0.64] | 0.103 |
| original_glcm_InverseVariance (median [IQR]) | 0.45[0.43,0.47] | 0.45[0.43,0.47] | 0.46[0.43,0.47] | 0.976 |
| original_glcm_JointAverage (median [IQR]) | 7.03[5.57,9.22] | 7.22[5.69,9.69] | 6.69[5.35,8.15] | 0.094 |
| original_glcm_JointEnergy (median [IQR]) | 0.13[0.11,0.17] | 0.13[0.11,0.16] | 0.14[0.11,0.19] | 0.371 |
| original_glcm_JointEntropy (median [IQR]) | 3.46[3.12,3.72] | 3.47[3.15,3.69] | 3.33[2.91,3.75] | 0.284 |
| original_glcm_MCC (median [IQR]) | 0.50[0.42,0.57] | 0.51[0.44,0.58] | 0.47[0.37,0.56] | 0.098 |
| original_glcm_MaximumProbability (median [IQR]) | 0.25[0.20,0.30] | 0.24[0.20,0.30] | 0.26[0.20,0.32] | 0.469 |
| original_glcm_SumAverage (median [IQR]) | 14.07[11.13,18.44] | 14.45[11.38,19.38] | 13.38[10.71,16.30] | 0.094 |
| original_glcm_SumEntropy (median [IQR]) | 2.61[2.40,2.78] | 2.63[2.40,2.79] | 2.57[2.23,2.73] | 0.121 |
| original_glcm_SumSquares (median [IQR]) | 0.80[0.62,0.98] | 0.81[0.63,0.99] | 0.78[0.52,0.92] | 0.149 |
| original_glrlm_GrayLevelNonUniformity (median [IQR]) | 3773.47[1693.44,6561.32] | 4177.14[2122.57,7294.02] | 2163.67[1253.66,4505.49] | 0.006 |
| original_glrlm_GrayLevelNonUniformityNormalized (median [IQR]) | 0.28[0.26,0.32] | 0.28[0.26,0.31] | 0.29[0.27,0.34] | 0.137 |
| original_glrlm_GrayLevelVariance (median [IQR]) | 1.01[0.84,1.25] | 1.02[0.87,1.26] | 1.01[0.73,1.17] | 0.124 |
| original_glrlm_HighGrayLevelRunEmphasis (median [IQR]) | 49.06[30.61,82.58] | 50.93[31.96,91.18] | 43.21[27.85,64.25] | 0.13 |
| original_glrlm_LongRunEmphasis (median [IQR]) | 4.70[4.05,5.70] | 4.77[4.09,5.67] | 4.43[3.79,5.80] | 0.512 |
| original_glrlm_LongRunHighGrayLevelEmphasis (median [IQR]) | 227.04[136.75,495.21] | 276.85[144.41,520.97] | 203.06[126.65,390.42] | 0.106 |
| original_glrlm_LongRunLowGrayLevelEmphasis (median [IQR]) | 0.11[0.06,0.16] | 0.11[0.05,0.16] | 0.12[0.08,0.18] | 0.165 |
| original_glrlm_LowGrayLevelRunEmphasis (median [IQR]) | 0.02[0.01,0.04] | 0.02[0.01,0.04] | 0.03[0.02,0.04] | 0.128 |
| original_glrlm_RunEntropy (median [IQR]) | 3.58[3.44,3.72] | 3.60[3.46,3.73] | 3.52[3.33,3.65] | 0.039 |
| original_glrlm_RunLengthNonUniformity (median [IQR]) | 6441.58[3063.88,11507.34] | 7366.83[3863.58,12948.63] | 3555.39[1840.85,7910.87] | 0.002 |
| original_glrlm_RunLengthNonUniformityNormalized (median [IQR]) | 0.47[0.43,0.51] | 0.47[0.44,0.50] | 0.48[0.42,0.52] | 0.43 |
| original_glrlm_RunPercentage (median [IQR]) | 0.62[0.58,0.66] | 0.61[0.58,0.65] | 0.62[0.55,0.67] | 0.324 |
| original_glrlm_RunVariance (median [IQR]) | 1.62[1.30,2.18] | 1.62[1.32,2.13] | 1.62[1.11,2.22] | 0.56 |
| original_glrlm_ShortRunEmphasis (median [IQR]) | 0.70[0.67,0.73] | 0.70[0.67,0.72] | 0.71[0.66,0.73] | 0.463 |
| original_glrlm_ShortRunHighGrayLevelEmphasis (median [IQR]) | 33.17[20.77,56.93] | 34.03[21.89,59.53] | 30.61[19.96,42.83] | 0.172 |
| original_glrlm_ShortRunLowGrayLevelEmphasis (median [IQR]) | 0.02[0.01,0.03] | 0.02[0.01,0.03] | 0.02[0.01,0.03] | 0.137 |
| original_glszm_GrayLevelNonUniformity (median [IQR]) | 127.44[64.04,201.52] | 142.92[85.84,226.82] | 75.42[43.05,146.82] | 0.006 |
| original_glszm_GrayLevelNonUniformityNormalized (median [IQR]) | 0.21[0.18,0.24] | 0.21[0.18,0.23] | 0.20[0.18,0.28] | 0.359 |
| original_glszm_GrayLevelVariance (median [IQR]) | 3.06[2.49,3.65] | 3.07[2.58,3.60] | 2.95[2.15,3.70] | 0.28 |
| original_glszm_HighGrayLevelZoneEmphasis (median [IQR]) | 40.13[26.70,68.04] | 41.68[27.41,79.27] | 37.17[24.46,52.80] | 0.235 |
| original_glszm_LargeAreaEmphasis (median [IQR]) | 197039.81[87032.17,433105.98] | 242324.50[107661.65,463475.81] | 131928.73[50497.88,302236.12] | 0.024 |
| original_glszm_LargeAreaHighGrayLevelEmphasis (median [IQR]) | 9988904.83[2809595.65,40837671.82] | 11795149.50[4367759.13,50887494.41] | 5208160.81[1852455.19,15209319.60] | 0.019 |
| original_glszm_LargeAreaLowGrayLevelEmphasis (median [IQR]) | 3226.91[2040.39,6374.39] | 3839.28[2077.61,7881.59] | 2391.42[1528.61,5816.75] | 0.084 |
| original_glszm_LowGrayLevelZoneEmphasis (median [IQR]) | 0.04[0.02,0.07] | 0.04[0.02,0.06] | 0.04[0.03,0.07] | 0.161 |
| original_glszm_SizeZoneNonUniformity (median [IQR]) | 157.92[94.61,303.29] | 171.64[110.30,320.02] | 100.31[51.22,228.24] | 0.008 |
| original_glszm_SizeZoneNonUniformityNormalized (median [IQR]) | 0.28[0.24,0.30] | 0.27[0.24,0.30] | 0.29[0.25,0.33] | 0.099 |
| original_glszm_SmallAreaEmphasis (median [IQR]) | 0.54[0.50,0.56] | 0.53[0.50,0.56] | 0.55[0.50,0.59] | 0.133 |
| original_glszm_SmallAreaHighGrayLevelEmphasis (median [IQR]) | 20.75[13.49,36.12] | 21.18[13.53,42.28] | 20.18[13.42,27.77] | 0.336 |
| original_glszm_SmallAreaLowGrayLevelEmphasis (median [IQR]) | 0.02[0.01,0.04] | 0.02[0.01,0.04] | 0.03[0.02,0.05] | 0.104 |
| original_glszm_ZoneEntropy (median [IQR]) | 5.12[4.86,5.37] | 5.16[4.93,5.39] | 4.94[4.50,5.24] | 0.011 |
| original_glszm_ZonePercentage (median [IQR]) | 0.03[0.02,0.04] | 0.03[0.02,0.03] | 0.03[0.02,0.04] | 0.384 |
| original_glszm_ZoneVariance (median [IQR]) | 183452.45 [78606.27, 399454.54] | 241113.22 [106615.87, 461873.99] | 100030.70 [43532.00, 190490.06] | 0.001 |
| original_ngtdm_Busyness (median [IQR]) | 20.88[13.62,34.69] | 21.78[14.63,39.89] | 19.26[11.30,29.01] | 0.066 |
| original_ngtdm_Coarseness (median [IQR]) | 0.00[0.00,0.00] | 0.00[0.00,0.00] | 0.00[0.00,0.00] | 0.005 |
| original_ngtdm_Complexity (median [IQR]) | 24.55[15.79,41.77] | 25.95[16.56,43.16] | 22.68[13.00,33.88] | 0.104 |
| original_ngtdm_Contrast (median [IQR]) | 0.01[0.01,0.01] | 0.01[0.01,0.01] | 0.01[0.01,0.01] | 0.477 |
| original_ngtdm_Strength (median [IQR]) | 0.03[0.02,0.05] | 0.03[0.02,0.04] | 0.03[0.02,0.06] | 0.18 |
| original_gldm_DependenceEntropy (median [IQR]) | 6.08[5.89,6.24] | 6.10[5.94,6.24] | 6.03[5.72,6.21] | 0.038 |
| original_gldm_DependenceNonUniformity (median [IQR]) | 1117.28[517.92,2178.83] | 1359.71[661.87,2397.25] | 634.06[302.21,1516.08] | 0.004 |
| original_gldm_DependenceNonUniformityNormalized (median [IQR]) | 0.05[0.05,0.06] | 0.05[0.05,0.06] | 0.05[0.05,0.06] | 0.307 |
| original_gldm_DependenceVariance (median [IQR]) | 28.42[24.15,32.08] | 28.65[25.22,32.18] | 27.44[23.01,31.85] | 0.338 |
| original_gldm_GrayLevelNonUniformity (median [IQR]) | 6906.15[3125.82,12804.22] | 7788.03[3645.81,13418.77] | 3845.13[1969.46,7894.40] | 0.008 |
| original_gldm_GrayLevelVariance (median [IQR]) | 0.86[0.67,1.09] | 0.87[0.69,1.09] | 0.85[0.55,0.98] | 0.148 |
| original_gldm_HighGrayLevelEmphasis (median [IQR]) | 49.54[31.27,83.94] | 51.53[32.34,93.34] | 44.24[28.41,66.50] | 0.108 |
| original_gldm_LargeDependenceEmphasis (median [IQR]) | 148.30[124.55,172.66] | 150.37[128.40,172.33] | 139.54[116.00,196.49] | 0.324 |
| original_gldm_LargeDependenceHighGrayLevelEmphasis (median [IQR]) | 6802.64[4496.39,13901.65] | 8091.21[4730.99,16077.90] | 5785.79[3804.57,11652.77] | 0.073 |
| original_gldm_LargeDependenceLowGrayLevelEmphasis (median [IQR]) | 3.26[1.72,4.60] | 3.23[1.55,4.61] | 3.52[2.41,4.59] | 0.288 |
| original_gldm_LowGrayLevelEmphasis (median [IQR]) | 0.02[0.01,0.04] | 0.02[0.01,0.03] | 0.02[0.02,0.04] | 0.115 |
| original_gldm_SmallDependenceEmphasis (median [IQR]) | 0.04[0.03,0.04] | 0.04[0.03,0.04] | 0.04[0.03,0.05] | 0.384 |
| original_gldm_SmallDependenceHighGrayLevelEmphasis (median [IQR]) | 1.54[1.05,2.89] | 1.57[1.04,3.07] | 1.39[1.05,2.21] | 0.42 |
| original_gldm_SmallDependenceLowGrayLevelEmphasis (median [IQR]) | 0.00[0.00,0.00] | 0.00[0.00,0.00] | 0.00[0.00,0.00] | 0.114 |
| log.sigma.1.0.mm.3D_firstorder_10Percentile (median [IQR]) | -21.25[-27.18,-17.24] | -21.41[-28.23,-17.06] | -20.80[-26.12,-17.47] | 0.591 |
| log.sigma.1.0.mm.3D_firstorder_90Percentile (median [IQR]) | 6.14[4.76,7.51] | 6.17[4.83,7.53] | 5.84[4.73,7.44] | 0.882 |
| log.sigma.1.0.mm.3D_firstorder_Energy (median [IQR]) | 7506580.63[2425900.48,21948415.46] | 8764913.18[3417504.60,27250339.60] | 3879028.22[1278201.67,14912411.01] | 0.021 |
| log.sigma.1.0.mm.3D_firstorder_Entropy (median [IQR]) | 1.30[1.15,1.51] | 1.30[1.15,1.52] | 1.29[1.16,1.50] | 0.779 |
| log.sigma.1.0.mm.3D_firstorder_InterquartileRange (median [IQR]) | 13.08[11.16,15.79] | 13.33[11.27,15.70] | 12.91[10.56,16.49] | 0.489 |
| log.sigma.1.0.mm.3D_firstorder_Kurtosis (median [IQR]) | 19.89[9.25,32.76] | 19.84[8.87,31.85] | 22.55[10.89,42.29] | 0.381 |
| log.sigma.1.0.mm.3D_firstorder_Maximum (median [IQR]) | 44.04[32.56,61.10] | 44.13[33.38,62.39] | 42.74[31.10,57.43] | 0.384 |
| log.sigma.1.0.mm.3D_firstorder_MeanAbsoluteDeviation (median [IQR]) | 10.02[7.92,12.90] | 9.96[7.91,13.19] | 10.13[8.43,12.43] | 0.751 |
| log.sigma.1.0.mm.3D_firstorder_Mean (median [IQR]) | -7.58[-10.83,-5.29] | -7.46[-11.02,-5.30] | -8.03[-9.30,-5.30] | 0.409 |
| log.sigma.1.0.mm.3D_firstorder_Median (median [IQR]) | -5.05[-6.59,-3.45] | -5.16[-6.55,-3.52] | -4.48[-6.59,-3.10] | 0.399 |
| log.sigma.1.0.mm.3D_firstorder_Minimum (median [IQR]) | -217.61[-289.46,-122.21] | -229.03[-293.70,-124.94] | -198.00[-253.74,-121.29] | 0.214 |
| log.sigma.1.0.mm.3D_firstorder_Range (median [IQR]) | 260.05[161.98,345.15] | 294.44[166.49,352.74] | 232.82[158.74,302.20] | 0.184 |
| log.sigma.1.0.mm.3D_firstorder_RobustMeanAbsoluteDeviation (median [IQR]) | 5.66[4.71,6.97] | 5.65[4.76,7.04] | 5.70[4.44,6.91] | 0.409 |
| log.sigma.1.0.mm.3D_firstorder_RootMeanSquared (median [IQR]) | 16.82[13.37,25.54] | 16.83[13.15,25.87] | 16.59[14.56,24.06] | 0.846 |
| log.sigma.1.0.mm.3D_firstorder_Skewness (median [IQR]) | -3.37[-4.16,-1.41] | -3.24[-4.06,-1.27] | -3.62[-4.32,-1.79] | 0.296 |
| log.sigma.1.0.mm.3D_firstorder_TotalEnergy (median [IQR]) | 15286733.87[4685370.70,47145861.50] | 16732627.29[5595258.17,52794805.24] | 6631749.08[2475221.86,33443996.76] | 0.019 |
| log.sigma.1.0.mm.3D_firstorder_Uniformity (median [IQR]) | 0.48[0.45,0.53] | 0.48[0.45,0.52] | 0.48[0.44,0.54] | 0.9 |
| log.sigma.1.0.mm.3D_firstorder_Variance (median [IQR]) | 251.13[134.40,520.73] | 249.77[126.77,577.42] | 268.24[149.49,458.47] | 0.8 |
| log.sigma.1.0.mm.3D_glcm_Autocorrelation (median [IQR]) | 86.17[29.27,147.31] | 102.15[33.44,150.31] | 66.21[28.20,127.37] | 0.248 |
| log.sigma.1.0.mm.3D_glcm_ClusterProminence (median [IQR]) | 42.44[3.31,126.32] | 44.79[3.17,149.72] | 27.25[3.86,85.89] | 0.696 |
| log.sigma.1.0.mm.3D_glcm_ClusterShade (median [IQR]) | -3.90[-12.01,-0.22] | -4.69[-13.43,-0.19] | -3.14[-8.74,-0.44] | 0.751 |
| log.sigma.1.0.mm.3D_glcm_ClusterTendency (median [IQR]) | 1.29[0.77,2.33] | 1.32[0.77,2.44] | 1.24[0.82,2.06] | 0.782 |
| log.sigma.1.0.mm.3D_glcm_Contrast (median [IQR]) | 0.52[0.42,0.77] | 0.52[0.42,0.79] | 0.51[0.45,0.65] | 0.754 |
| log.sigma.1.0.mm.3D_glcm_Correlation (median [IQR]) | 0.40[0.28,0.48] | 0.40[0.28,0.48] | 0.38[0.29,0.49] | 0.747 |
| log.sigma.1.0.mm.3D_glcm_DifferenceAverage (median [IQR]) | 0.44[0.39,0.50] | 0.44[0.39,0.51] | 0.44[0.39,0.48] | 0.62 |
| log.sigma.1.0.mm.3D_glcm_DifferenceEntropy (median [IQR]) | 1.10[1.00,1.23] | 1.10[1.00,1.25] | 1.08[1.03,1.18] | 0.682 |
| log.sigma.1.0.mm.3D_glcm_DifferenceVariance (median [IQR]) | 0.32[0.26,0.49] | 0.32[0.25,0.51] | 0.32[0.28,0.46] | 0.965 |
| log.sigma.1.0.mm.3D_glcm_Id (median [IQR]) | 0.79[0.78,0.81] | 0.79[0.78,0.81] | 0.79[0.78,0.82] | 0.842 |
| log.sigma.1.0.mm.3D_glcm_Idm (median [IQR]) | 0.79[0.77,0.81] | 0.79[0.77,0.81] | 0.79[0.77,0.81] | 0.768 |
| log.sigma.1.0.mm.3D_glcm_Idmn (median [IQR]) | 1.00[0.99,1.00] | 1.00[0.99,1.00] | 0.99[0.99,1.00] | 0.305 |
| log.sigma.1.0.mm.3D_glcm_Idn (median [IQR]) | 0.96[0.95,0.97] | 0.96[0.96,0.97] | 0.96[0.95,0.97] | 0.27 |
| log.sigma.1.0.mm.3D_glcm_Imc1 (median [IQR]) | -0.11[-0.13,-0.08] | -0.11[-0.13,-0.09] | -0.11[-0.13,-0.08] | 0.973 |
| log.sigma.1.0.mm.3D_glcm_Imc2 (median [IQR]) | 0.42[0.33,0.49] | 0.42[0.33,0.50] | 0.43[0.34,0.49] | 0.958 |
| log.sigma.1.0.mm.3D_glcm_InverseVariance (median [IQR]) | 0.38[0.35,0.39] | 0.38[0.35,0.39] | 0.38[0.34,0.40] | 0.617 |
| log.sigma.1.0.mm.3D_glcm_JointAverage (median [IQR]) | 9.28[5.40,12.12] | 10.09[5.76,12.25] | 8.13[5.30,11.27] | 0.248 |
| log.sigma.1.0.mm.3D_glcm_JointEnergy (median [IQR]) | 0.27[0.24,0.31] | 0.27[0.24,0.30] | 0.27[0.24,0.32] | 0.918 |
| log.sigma.1.0.mm.3D_glcm_JointEntropy (median [IQR]) | 2.36[2.14,2.66] | 2.37[2.14,2.66] | 2.32[2.15,2.64] | 0.643 |
| log.sigma.1.0.mm.3D_glcm_MCC (median [IQR]) | 0.53[0.38,0.62] | 0.53[0.38,0.63] | 0.52[0.42,0.62] | 0.991 |
| log.sigma.1.0.mm.3D_glcm_MaximumProbability (median [IQR]) | 0.42[0.38,0.49] | 0.43[0.38,0.48] | 0.42[0.37,0.51] | 0.61 |
| log.sigma.1.0.mm.3D_glcm_SumAverage (median [IQR]) | 18.55[10.80,24.24] | 20.17[11.53,24.50] | 16.25[10.60,22.55] | 0.248 |
| log.sigma.1.0.mm.3D_glcm_SumEntropy (median [IQR]) | 1.86[1.70,2.09] | 1.86[1.70,2.09] | 1.87[1.72,2.08] | 0.727 |
| log.sigma.1.0.mm.3D_glcm_SumSquares (median [IQR]) | 0.45[0.30,0.77] | 0.46[0.29,0.79] | 0.43[0.33,0.70] | 0.814 |
| log.sigma.1.0.mm.3D_glrlm_GrayLevelNonUniformity (median [IQR]) | 4623.97[2208.27,8612.33] | 5261.54[2556.91,9421.72] | 2638.32[1322.63,5817.48] | 0.006 |
| log.sigma.1.0.mm.3D_glrlm_GrayLevelNonUniformityNormalized (median [IQR]) | 0.42[0.36,0.46] | 0.41[0.36,0.46] | 0.42[0.37,0.46] | 0.509 |
| log.sigma.1.0.mm.3D_glrlm_GrayLevelVariance (median [IQR]) | 0.82[0.45,1.61] | 0.83[0.42,1.69] | 0.81[0.48,1.40] | 0.779 |
| log.sigma.1.0.mm.3D_glrlm_HighGrayLevelRunEmphasis (median [IQR]) | 86.27[29.14,143.54] | 98.72[32.77,146.96] | 64.50[28.17,125.82] | 0.237 |
| log.sigma.1.0.mm.3D_glrlm_LongRunEmphasis (median [IQR]) | 8.38[7.25,9.57] | 8.39[7.56,9.50] | 7.87[7.03,9.73] | 0.29 |
| log.sigma.1.0.mm.3D_glrlm_LongRunHighGrayLevelEmphasis (median [IQR]) | 733.27[255.31,1218.03] | 787.91[277.86,1239.37] | 487.08[194.04,1033.15] | 0.213 |
| log.sigma.1.0.mm.3D_glrlm_LongRunLowGrayLevelEmphasis (median [IQR]) | 0.10[0.06,0.29] | 0.09[0.06,0.27] | 0.13[0.07,0.28] | 0.32 |
| log.sigma.1.0.mm.3D_glrlm_LowGrayLevelRunEmphasis (median [IQR]) | 0.01[0.01,0.04] | 0.01[0.01,0.03] | 0.02[0.01,0.04] | 0.214 |
| log.sigma.1.0.mm.3D_glrlm_RunEntropy (median [IQR]) | 3.45[3.23,3.63] | 3.47[3.26,3.69] | 3.36[3.15,3.57] | 0.072 |
| log.sigma.1.0.mm.3D_glrlm_RunLengthNonUniformity (median [IQR]) | 4506.77[2101.33,8177.94] | 5103.40[2482.44,8837.81] | 2482.80[1605.37,6143.25] | 0.005 |
| log.sigma.1.0.mm.3D_glrlm_RunLengthNonUniformityNormalized (median [IQR]) | 0.37[0.35,0.40] | 0.37[0.34,0.40] | 0.38[0.35,0.41] | 0.282 |
| log.sigma.1.0.mm.3D_glrlm_RunPercentage (median [IQR]) | 0.52[0.49,0.55] | 0.52[0.49,0.54] | 0.53[0.50,0.56] | 0.181 |
| log.sigma.1.0.mm.3D_glrlm_RunVariance (median [IQR]) | 3.30[2.71,3.80] | 3.31[2.86,3.78] | 3.05[2.45,3.83] | 0.223 |
| log.sigma.1.0.mm.3D_glrlm_ShortRunEmphasis (median [IQR]) | 0.60[0.58,0.63] | 0.60[0.58,0.63] | 0.61[0.59,0.64] | 0.29 |
| log.sigma.1.0.mm.3D_glrlm_ShortRunHighGrayLevelEmphasis (median [IQR]) | 50.50[18.47,84.72] | 57.52[19.30,87.70] | 40.85[18.57,69.37] | 0.237 |
| log.sigma.1.0.mm.3D_glrlm_ShortRunLowGrayLevelEmphasis (median [IQR]) | 0.01[0.01,0.02] | 0.01[0.01,0.02] | 0.01[0.01,0.03] | 0.175 |
| log.sigma.1.0.mm.3D_glszm_GrayLevelNonUniformity (median [IQR]) | 73.50[39.67,111.85] | 75.23[45.79,119.56] | 41.45[27.74,94.67] | 0.003 |
| log.sigma.1.0.mm.3D_glszm_GrayLevelNonUniformityNormalized (median [IQR]) | 0.20[0.14,0.35] | 0.20[0.13,0.35] | 0.21[0.15,0.33] | 0.53 |
| log.sigma.1.0.mm.3D_glszm_GrayLevelVariance (median [IQR]) | 4.99[1.77,6.48] | 5.15[1.74,6.51] | 4.35[1.80,6.32] | 0.754 |
| log.sigma.1.0.mm.3D_glszm_HighGrayLevelZoneEmphasis (median [IQR]) | 63.34[27.02,98.79] | 66.02[29.75,98.30] | 46.75[22.24,99.39] | 0.237 |
| log.sigma.1.0.mm.3D_glszm_LargeAreaEmphasis (median [IQR]) | 605295.89[260175.92,1365502.90] | 835133.20[324082.48,1575326.07] | 391323.12[145334.85,867001.65] | 0.016 |
| log.sigma.1.0.mm.3D_glszm_LargeAreaHighGrayLevelEmphasis (median [IQR]) | 47866865.73[9796998.54,148589157.00] | 60029954.53[12382223.83,157656330.57] | 24912042.72[3851367.77,74213844.90] | 0.023 |
| log.sigma.1.0.mm.3D_glszm_LargeAreaLowGrayLevelEmphasis (median [IQR]) | 9613.77[3584.32,32012.72] | 13213.15[3881.45,35464.74] | 7716.37[2627.21,22363.75] | 0.125 |
| log.sigma.1.0.mm.3D_glszm_LowGrayLevelZoneEmphasis (median [IQR]) | 0.03[0.02,0.07] | 0.03[0.02,0.06] | 0.05[0.02,0.08] | 0.089 |
| log.sigma.1.0.mm.3D_glszm_SizeZoneNonUniformity (median [IQR]) | 66.46[31.69,137.31] | 74.71[36.04,151.61] | 42.51[19.30,108.17] | 0.033 |
| log.sigma.1.0.mm.3D_glszm_SizeZoneNonUniformityNormalized (median [IQR]) | 0.20[0.18,0.23] | 0.20[0.18,0.23] | 0.21[0.19,0.24] | 0.137 |
| log.sigma.1.0.mm.3D_glszm_SmallAreaEmphasis (median [IQR]) | 0.45[0.41,0.48] | 0.44[0.41,0.48] | 0.45[0.43,0.50] | 0.322 |
| log.sigma.1.0.mm.3D_glszm_SmallAreaHighGrayLevelEmphasis (median [IQR]) | 27.92[12.57,43.08] | 30.35[13.40,42.87] | 19.39[9.98,43.11] | 0.307 |
| log.sigma.1.0.mm.3D_glszm_SmallAreaLowGrayLevelEmphasis (median [IQR]) | 0.02[0.01,0.03] | 0.01[0.01,0.02] | 0.03[0.01,0.04] | 0.037 |
| log.sigma.1.0.mm.3D_glszm_ZoneEntropy (median [IQR]) | 5.37[4.73,5.94] | 5.48[4.79,5.96] | 5.14[4.55,5.71] | 0.104 |
| log.sigma.1.0.mm.3D_glszm_ZonePercentage (median [IQR]) | 0.02[0.01,0.02] | 0.02[0.01,0.02] | 0.02[0.01,0.02] | 0.43 |
| log.sigma.1.0.mm.3D_glszm_ZoneVariance (median [IQR]) | 602184.51[257373.69,1360762.82] | 831416.17[322381.97,1568282.88] | 390218.25[142197.83,860867.65] | 0.016 |
| log.sigma.1.0.mm.3D_ngtdm_Busyness (median [IQR]) | 22.62[11.01,49.60] | 25.12[13.06,52.79] | 16.66[10.38,44.47] | 0.08 |
| log.sigma.1.0.mm.3D_ngtdm_Coarseness (median [IQR]) | 0.00[0.00,0.00] | 0.00[0.00,0.00] | 0.00[0.00,0.00] | 0.006 |
| log.sigma.1.0.mm.3D_ngtdm_Complexity (median [IQR]) | 30.08[9.47,56.03] | 36.74[9.42,56.85] | 23.56[9.72,42.17] | 0.234 |
| log.sigma.1.0.mm.3D_ngtdm_Contrast (median [IQR]) | 0.00[0.00,0.01] | 0.00[0.00,0.01] | 0.00[0.00,0.01] | 0.463 |
| log.sigma.1.0.mm.3D_ngtdm_Strength (median [IQR]) | 0.05[0.02,0.09] | 0.04[0.02,0.09] | 0.07[0.02,0.11] | 0.142 |
| log.sigma.1.0.mm.3D_gldm_DependenceEntropy (median [IQR]) | 5.53[5.37,5.70] | 5.55[5.38,5.74] | 5.51[5.37,5.67] | 0.331 |
| log.sigma.1.0.mm.3D_gldm_DependenceNonUniformity (median [IQR]) | 1066.95[450.48,2039.02] | 1253.15[553.38,2187.62] | 565.42[320.66,1305.37] | 0.005 |
| log.sigma.1.0.mm.3D_gldm_DependenceNonUniformityNormalized (median [IQR]) | 0.05[0.05,0.05] | 0.05[0.05,0.05] | 0.05[0.05,0.05] | 0.065 |
| log.sigma.1.0.mm.3D_gldm_DependenceVariance (median [IQR]) | 32.13[29.46,35.73] | 32.53[29.56,36.91] | 31.62[28.94,33.88] | 0.198 |
| log.sigma.1.0.mm.3D_gldm_GrayLevelNonUniformity (median [IQR]) | 10807.73[4857.95,18668.72] | 12330.42[6236.50,21368.67] | 6181.61[2696.35,12145.29] | 0.003 |
| log.sigma.1.0.mm.3D_gldm_GrayLevelVariance (median [IQR]) | 0.54[0.33,0.98] | 0.53[0.31,1.03] | 0.55[0.36,0.85] | 0.867 |
| log.sigma.1.0.mm.3D_gldm_HighGrayLevelEmphasis (median [IQR]) | 85.83[29.24,146.10] | 101.35[33.09,149.24] | 65.40[28.09,126.67] | 0.252 |
| log.sigma.1.0.mm.3D_gldm_LargeDependenceEmphasis (median [IQR]) | 214.12[196.37,232.23] | 216.05[199.91,232.58] | 205.12[183.93,231.95] | 0.153 |
| log.sigma.1.0.mm.3D_gldm_LargeDependenceHighGrayLevelEmphasis (median [IQR]) | 19435.05[6638.71,31274.47] | 21473.12[6871.16,31766.58] | 14179.35[4458.23,25135.72] | 0.209 |
| log.sigma.1.0.mm.3D_gldm_LargeDependenceLowGrayLevelEmphasis (median [IQR]) | 2.45[1.49,6.22] | 2.31[1.40,6.87] | 3.19[1.94,6.00] | 0.307 |
| log.sigma.1.0.mm.3D_gldm_LowGrayLevelEmphasis (median [IQR]) | 0.01[0.01,0.04] | 0.01[0.01,0.03] | 0.02[0.01,0.04] | 0.221 |
| log.sigma.1.0.mm.3D_gldm_SmallDependenceEmphasis (median [IQR]) | 0.02[0.02,0.03] | 0.02[0.02,0.03] | 0.02[0.02,0.03] | 0.278 |
| log.sigma.1.0.mm.3D_gldm_SmallDependenceHighGrayLevelEmphasis (median [IQR]) | 1.49[0.54,2.66] | 1.57[0.56,2.71] | 1.42[0.55,2.07] | 0.359 |
| log.sigma.1.0.mm.3D_gldm_SmallDependenceLowGrayLevelEmphasis (median [IQR]) | 0.00[0.00,0.00] | 0.00[0.00,0.00] | 0.00[0.00,0.00] | 0.053 |
| log.sigma.1.5.mm.3D_firstorder_10Percentile (median [IQR]) | -30.43[-48.71,-23.82] | -31.01[-50.22,-23.87] | -28.91[-43.93,-23.80] | 0.466 |
| log.sigma.1.5.mm.3D_firstorder_90Percentile (median [IQR]) | 3.82[2.36,5.58] | 3.86[2.31,5.63] | 3.63[2.50,5.31] | 0.98 |
| log.sigma.1.5.mm.3D_firstorder_Energy (median [IQR]) | 15034575.10[4633947.80,61778447.49] | 20962312.71[5383712.73,65826906.96] | 8296923.18[2497910.26,37626726.71] | 0.027 |
| log.sigma.1.5.mm.3D_firstorder_Entropy (median [IQR]) | 1.50[1.24,1.81] | 1.51[1.24,1.85] | 1.46[1.24,1.78] | 0.509 |
| log.sigma.1.5.mm.3D_firstorder_InterquartileRange (median [IQR]) | 16.74[13.64,20.99] | 16.91[13.73,21.76] | 15.07[13.07,20.31] | 0.237 |
| log.sigma.1.5.mm.3D_firstorder_Kurtosis (median [IQR]) | 15.91[7.43,26.25] | 15.93[7.00,24.02] | 15.89[10.13,33.68] | 0.276 |
| log.sigma.1.5.mm.3D_firstorder_Maximum (median [IQR]) | 48.99[37.12,74.60] | 49.49[37.11,74.75] | 48.08[37.74,68.93] | 0.676 |
| log.sigma.1.5.mm.3D_firstorder_MeanAbsoluteDeviation (median [IQR]) | 12.73[9.98,19.90] | 12.80[9.74,20.92] | 12.59[10.35,17.99] | 0.646 |
| log.sigma.1.5.mm.3D_firstorder_Mean (median [IQR]) | -13.70[-19.98,-9.92] | -13.96[-20.32,-9.92] | -13.08[-18.96,-10.78] | 0.486 |
| log.sigma.1.5.mm.3D_firstorder_Median (median [IQR]) | -9.77[-13.20,-6.54] | -9.88[-13.24,-6.59] | -8.64[-13.07,-5.91] | 0.404 |
| log.sigma.1.5.mm.3D_firstorder_Minimum (median [IQR]) | -286.34[-345.18,-152.29] | -296.33[-352.17,-152.19] | -224.58[-332.55,-152.54] | 0.305 |
| log.sigma.1.5.mm.3D_firstorder_Range (median [IQR]) | 330.61[203.52,416.30] | 350.14[198.98,424.85] | 291.79[207.51,374.87] | 0.221 |
| log.sigma.1.5.mm.3D_firstorder_RobustMeanAbsoluteDeviation (median [IQR]) | 7.24[5.76,9.30] | 7.28[5.77,9.49] | 7.20[5.56,8.66] | 0.414 |
| log.sigma.1.5.mm.3D_firstorder_RootMeanSquared (median [IQR]) | 26.49[18.27,40.14] | 26.73[18.05,40.79] | 26.26[19.22,35.86] | 0.761 |
| log.sigma.1.5.mm.3D_firstorder_Skewness (median [IQR]) | -2.90[-4.00,-1.67] | -2.79[-3.84,-1.32] | -3.21[-4.53,-2.18] | 0.158 |
| log.sigma.1.5.mm.3D_firstorder_TotalEnergy (median [IQR]) | 31198082.54[8767298.92,118683378.02] | 37771431.50[11237628.69,131765357.84] | 17231358.76[4345105.76,100004928.95] | 0.024 |
| log.sigma.1.5.mm.3D_firstorder_Uniformity (median [IQR]) | 0.46[0.39,0.52] | 0.46[0.38,0.52] | 0.48[0.40,0.56] | 0.412 |
| log.sigma.1.5.mm.3D_firstorder_Variance (median [IQR]) | 546.92[203.44,1258.90] | 564.02[190.75,1303.05] | 529.81[229.59,942.35] | 0.821 |
| log.sigma.1.5.mm.3D_glcm_Autocorrelation (median [IQR]) | 140.11[49.20,201.51] | 148.21[48.59,202.16] | 87.55[50.05,196.58] | 0.334 |
| log.sigma.1.5.mm.3D_glcm_ClusterProminence (median [IQR]) | 196.24[9.98,645.22] | 213.53[6.92,702.53] | 134.98[16.35,451.33] | 0.709 |
| log.sigma.1.5.mm.3D_glcm_ClusterShade (median [IQR]) | -15.83[-47.85,-1.03] | -16.50[-51.23,-0.61] | -14.48[-30.91,-2.04] | 0.703 |
| log.sigma.1.5.mm.3D_glcm_ClusterTendency (median [IQR]) | 2.53[1.04,5.77] | 2.77[1.04,5.89] | 2.33[1.19,4.48] | 0.73 |
| log.sigma.1.5.mm.3D_glcm_Contrast (median [IQR]) | 0.58[0.39,0.99] | 0.59[0.38,1.03] | 0.49[0.42,0.80] | 0.623 |
| log.sigma.1.5.mm.3D_glcm_Correlation (median [IQR]) | 0.58[0.43,0.68] | 0.58[0.42,0.67] | 0.57[0.46,0.69] | 0.86 |
| log.sigma.1.5.mm.3D_glcm_DifferenceAverage (median [IQR]) | 0.42[0.34,0.52] | 0.44[0.34,0.55] | 0.40[0.34,0.49] | 0.441 |
| log.sigma.1.5.mm.3D_glcm_DifferenceEntropy (median [IQR]) | 1.11[0.97,1.32] | 1.12[0.96,1.34] | 1.07[0.98,1.25] | 0.548 |
| log.sigma.1.5.mm.3D_glcm_DifferenceVariance (median [IQR]) | 0.36[0.26,0.69] | 0.36[0.25,0.71] | 0.36[0.28,0.57] | 0.754 |
| log.sigma.1.5.mm.3D_glcm_Id (median [IQR]) | 0.81[0.78,0.84] | 0.81[0.78,0.84] | 0.82[0.79,0.85] | 0.371 |
| log.sigma.1.5.mm.3D_glcm_Idm (median [IQR]) | 0.81[0.78,0.84] | 0.80[0.77,0.84] | 0.81[0.78,0.84] | 0.362 |
| log.sigma.1.5.mm.3D_glcm_Idmn (median [IQR]) | 1.00[0.99,1.00] | 1.00[0.99,1.00] | 1.00[0.99,1.00] | 0.944 |
| log.sigma.1.5.mm.3D_glcm_Idn (median [IQR]) | 0.97[0.96,0.98] | 0.97[0.96,0.98] | 0.97[0.96,0.98] | 0.922 |
| log.sigma.1.5.mm.3D_glcm_Imc1 (median [IQR]) | -0.20[-0.22,-0.16] | -0.20[-0.22,-0.16] | -0.20[-0.22,-0.17] | 0.832 |
| log.sigma.1.5.mm.3D_glcm_Imc2 (median [IQR]) | 0.59[0.49,0.67] | 0.59[0.47,0.66] | 0.58[0.50,0.67] | 0.995 |
| log.sigma.1.5.mm.3D_glcm_InverseVariance (median [IQR]) | 0.32[0.29,0.35] | 0.32[0.30,0.34] | 0.32[0.28,0.36] | 0.81 |
| log.sigma.1.5.mm.3D_glcm_JointAverage (median [IQR]) | 11.74[7.00,14.19] | 12.15[6.95,14.20] | 9.33[7.06,14.00] | 0.345 |
| log.sigma.1.5.mm.3D_glcm_JointEnergy (median [IQR]) | 0.28[0.23,0.35] | 0.28[0.23,0.34] | 0.29[0.23,0.42] | 0.458 |
| log.sigma.1.5.mm.3D_glcm_JointEntropy (median [IQR]) | 2.56[2.16,3.05] | 2.58[2.16,3.08] | 2.52[2.17,2.95] | 0.391 |
| log.sigma.1.5.mm.3D_glcm_MCC (median [IQR]) | 0.66[0.50,0.76] | 0.65[0.50,0.76] | 0.67[0.55,0.74] | 0.786 |
| log.sigma.1.5.mm.3D_glcm_MaximumProbability (median [IQR]) | 0.47[0.40,0.56] | 0.47[0.40,0.54] | 0.48[0.38,0.62] | 0.754 |
| log.sigma.1.5.mm.3D_glcm_SumAverage (median [IQR]) | 23.48[14.00,28.38] | 24.30[13.90,28.41] | 18.66[14.12,27.99] | 0.345 |
| log.sigma.1.5.mm.3D_glcm_SumEntropy (median [IQR]) | 2.06[1.77,2.43] | 2.07[1.78,2.46] | 2.05[1.77,2.37] | 0.409 |
| log.sigma.1.5.mm.3D_glcm_SumSquares (median [IQR]) | 0.80[0.36,1.70] | 0.88[0.35,1.73] | 0.74[0.40,1.32] | 0.737 |
| log.sigma.1.5.mm.3D_glrlm_GrayLevelNonUniformity (median [IQR]) | 3511.76[1804.55,6861.97] | 4008.09[2059.75,7283.07] | 2185.87[1074.92,4821.89] | 0.008 |
| log.sigma.1.5.mm.3D_glrlm_GrayLevelNonUniformityNormalized (median [IQR]) | 0.36[0.28,0.42] | 0.36[0.27,0.42] | 0.37[0.31,0.43] | 0.324 |
| log.sigma.1.5.mm.3D_glrlm_GrayLevelVariance (median [IQR]) | 1.83[0.59,3.57] | 1.87[0.58,3.82] | 1.58[0.77,2.65] | 0.758 |
| log.sigma.1.5.mm.3D_glrlm_HighGrayLevelRunEmphasis (median [IQR]) | 128.60[48.02,194.24] | 145.19[48.21,194.51] | 82.94[48.38,187.84] | 0.34 |
| log.sigma.1.5.mm.3D_glrlm_LongRunEmphasis (median [IQR]) | 13.08[9.88,15.84] | 13.47[9.96,16.15] | 12.23[9.73,14.94] | 0.506 |
| log.sigma.1.5.mm.3D_glrlm_LongRunHighGrayLevelEmphasis (median [IQR]) | 1537.32[581.66,2530.92] | 1676.80[637.31,2576.38] | 1205.63[558.81,2300.80] | 0.286 |
| log.sigma.1.5.mm.3D_glrlm_LongRunLowGrayLevelEmphasis (median [IQR]) | 0.11[0.06,0.30] | 0.10[0.06,0.30] | 0.12[0.08,0.29] | 0.433 |
| log.sigma.1.5.mm.3D_glrlm_LowGrayLevelRunEmphasis (median [IQR]) | 0.01[0.01,0.03] | 0.01[0.01,0.03] | 0.01[0.01,0.02] | 0.29 |
| log.sigma.1.5.mm.3D_glrlm_RunEntropy (median [IQR]) | 3.87[3.59,4.13] | 3.91[3.62,4.14] | 3.77[3.49,4.04] | 0.084 |
| log.sigma.1.5.mm.3D_glrlm_RunLengthNonUniformity (median [IQR]) | 4296.52[2023.70,7600.43] | 4548.82[2304.93,8644.78] | 2360.59[1284.81,5646.88] | 0.003 |
| log.sigma.1.5.mm.3D_glrlm_RunLengthNonUniformityNormalized (median [IQR]) | 0.36[0.32,0.40] | 0.36[0.32,0.41] | 0.37[0.33,0.40] | 0.709 |
| log.sigma.1.5.mm.3D_glrlm_RunPercentage (median [IQR]) | 0.49[0.45,0.53] | 0.49[0.45,0.53] | 0.49[0.47,0.53] | 0.452 |
| log.sigma.1.5.mm.3D_glrlm_RunVariance (median [IQR]) | 6.04[4.44,7.59] | 6.15[4.49,7.73] | 5.46[4.12,7.45] | 0.441 |
| log.sigma.1.5.mm.3D_glrlm_ShortRunEmphasis (median [IQR]) | 0.60[0.56,0.64] | 0.60[0.55,0.65] | 0.61[0.57,0.64] | 0.73 |
| log.sigma.1.5.mm.3D_glrlm_ShortRunHighGrayLevelEmphasis (median [IQR]) | 76.03[28.69,110.59] | 84.10[28.44,116.30] | 50.14[28.98,97.53] | 0.309 |
| log.sigma.1.5.mm.3D_glrlm_ShortRunLowGrayLevelEmphasis (median [IQR]) | 0.01[0.00,0.02] | 0.01[0.00,0.02] | 0.01[0.01,0.02] | 0.26 |
| log.sigma.1.5.mm.3D_glszm_GrayLevelNonUniformity (median [IQR]) | 55.81[30.03,87.71] | 59.25[39.52,92.99] | 33.64[19.84,66.64] | 0.001 |
| log.sigma.1.5.mm.3D_glszm_GrayLevelNonUniformityNormalized (median [IQR]) | 0.16[0.10,0.30] | 0.15[0.10,0.31] | 0.16[0.11,0.25] | 0.751 |
| log.sigma.1.5.mm.3D_glszm_GrayLevelVariance (median [IQR]) | 8.07[2.25,10.86] | 8.21[2.17,11.41] | 7.29[3.69,9.88] | 0.585 |
| log.sigma.1.5.mm.3D_glszm_HighGrayLevelZoneEmphasis (median [IQR]) | 91.18[41.65,134.09] | 102.33[44.94,134.49] | 65.15[35.90,123.75] | 0.272 |
| log.sigma.1.5.mm.3D_glszm_LargeAreaEmphasis (median [IQR]) | 624302.43[240978.83,1429741.21] | 775713.84[267791.78,1621242.39] | 429709.22[155853.10,940003.99] | 0.021 |
| log.sigma.1.5.mm.3D_glszm_LargeAreaHighGrayLevelEmphasis (median [IQR]) | 68193359.50[11978992.86,185808234.95] | 89505581.05[15471127.18,217042625.09] | 33884613.83[5061745.26,103602356.40] | 0.031 |
| log.sigma.1.5.mm.3D_glszm_LargeAreaLowGrayLevelEmphasis (median [IQR]) | 7537.42[2282.42,23235.11] | 10308.98[2525.01,24834.91] | 4124.81[1654.00,20711.06] | 0.148 |
| log.sigma.1.5.mm.3D_glszm_LowGrayLevelZoneEmphasis (median [IQR]) | 0.03[0.02,0.05] | 0.02[0.02,0.04] | 0.03[0.02,0.05] | 0.142 |
| log.sigma.1.5.mm.3D_glszm_SizeZoneNonUniformity (median [IQR]) | 43.49[23.28,109.76] | 47.18[25.63,118.77] | 30.65[14.50,77.25] | 0.043 |
| log.sigma.1.5.mm.3D_glszm_SizeZoneNonUniformityNormalized (median [IQR]) | 0.16[0.14,0.18] | 0.16[0.13,0.17] | 0.16[0.14,0.18] | 0.272 |
| log.sigma.1.5.mm.3D_glszm_SmallAreaEmphasis (median [IQR]) | 0.39[0.35,0.42] | 0.38[0.35,0.41] | 0.39[0.36,0.42] | 0.329 |
| log.sigma.1.5.mm.3D_glszm_SmallAreaHighGrayLevelEmphasis (median [IQR]) | 35.60[16.30,52.21] | 40.39[16.79,52.83] | 25.55[14.67,50.27] | 0.282 |
| log.sigma.1.5.mm.3D_glszm_SmallAreaLowGrayLevelEmphasis (median [IQR]) | 0.01[0.01,0.02] | 0.01[0.01,0.02] | 0.02[0.01,0.03] | 0.237 |
| log.sigma.1.5.mm.3D_glszm_ZoneEntropy (median [IQR]) | 6.01[5.20,6.57] | 6.16[5.23,6.62] | 5.84[5.04,6.24] | 0.084 |
| log.sigma.1.5.mm.3D_glszm_ZonePercentage (median [IQR]) | 0.01[0.01,0.02] | 0.01[0.01,0.02] | 0.02[0.01,0.02] | 0.444 |
| log.sigma.1.5.mm.3D_glszm_ZoneVariance (median [IQR]) | 621299.05[240068.20,1420544.58] | 768028.39[267063.80,1610367.08] | 424092.06[153172.84,925286.83] | 0.02 |
| log.sigma.1.5.mm.3D_ngtdm_Busyness (median [IQR]) | 13.62[6.21,28.64] | 14.92[7.07,30.57] | 7.13[5.18,21.47] | 0.033 |
| log.sigma.1.5.mm.3D_ngtdm_Coarseness (median [IQR]) | 0.00[0.00,0.00] | 0.00[0.00,0.00] | 0.00[0.00,0.00] | 0.005 |
| log.sigma.1.5.mm.3D_ngtdm_Complexity (median [IQR]) | 44.77[12.60,72.24] | 53.07[12.98,75.85] | 29.16[12.90,54.40] | 0.258 |
| log.sigma.1.5.mm.3D_ngtdm_Contrast (median [IQR]) | 0.01[0.00,0.01] | 0.01[0.00,0.01] | 0.00[0.00,0.01] | 0.864 |
| log.sigma.1.5.mm.3D_ngtdm_Strength (median [IQR]) | 0.08[0.03,0.19] | 0.07[0.03,0.18] | 0.13[0.04,0.21] | 0.071 |
| log.sigma.1.5.mm.3D_gldm_DependenceEntropy (median [IQR]) | 5.79[5.54,6.05] | 5.80[5.56,6.07] | 5.74[5.46,5.97] | 0.307 |
| log.sigma.1.5.mm.3D_gldm_DependenceNonUniformity (median [IQR]) | 1029.19[447.91,1870.95] | 1171.86[554.28,2058.64] | 583.22[290.34,1247.78] | 0.004 |
| log.sigma.1.5.mm.3D_gldm_DependenceNonUniformityNormalized (median [IQR]) | 0.05[0.04,0.05] | 0.05[0.04,0.05] | 0.05[0.04,0.05] | 0.575 |
| log.sigma.1.5.mm.3D_gldm_DependenceVariance (median [IQR]) | 41.57[37.64,46.12] | 42.08[37.69,46.89] | 39.86[37.57,44.90] | 0.264 |
| log.sigma.1.5.mm.3D_gldm_GrayLevelNonUniformity (median [IQR]) | 9841.07[4465.06,18061.44] | 11303.71[5361.72,19686.64] | 5920.48[2521.99,10943.20] | 0.006 |
| log.sigma.1.5.mm.3D_gldm_GrayLevelVariance (median [IQR]) | 0.96[0.41,2.14] | 1.01[0.40,2.15] | 0.92[0.47,1.58] | 0.832 |
| log.sigma.1.5.mm.3D_gldm_HighGrayLevelEmphasis (median [IQR]) | 139.07[48.86,201.12] | 147.17[48.46,201.48] | 86.63[49.37,194.69] | 0.331 |
| log.sigma.1.5.mm.3D_gldm_LargeDependenceEmphasis (median [IQR]) | 247.68[215.24,273.91] | 249.44[215.07,275.88] | 236.48[217.65,266.18] | 0.412 |
| log.sigma.1.5.mm.3D_gldm_LargeDependenceHighGrayLevelEmphasis (median [IQR]) | 32544.61[11887.53,51251.63] | 34182.46[12043.71,51852.37] | 21839.27[11261.29,43787.54] | 0.324 |
| log.sigma.1.5.mm.3D_gldm_LargeDependenceLowGrayLevelEmphasis (median [IQR]) | 1.82[1.15,5.06] | 1.71[1.12,5.11] | 2.34[1.42,4.80] | 0.329 |
| log.sigma.1.5.mm.3D_gldm_LowGrayLevelEmphasis (median [IQR]) | 0.01[0.01,0.02] | 0.01[0.01,0.02] | 0.01[0.01,0.02] | 0.32 |
| log.sigma.1.5.mm.3D_gldm_SmallDependenceEmphasis (median [IQR]) | 0.02[0.02,0.03] | 0.02[0.02,0.03] | 0.02[0.02,0.03] | 0.412 |
| log.sigma.1.5.mm.3D_gldm_SmallDependenceHighGrayLevelEmphasis (median [IQR]) | 2.12[0.72,3.64] | 2.29[0.69,4.18] | 1.89[0.73,3.29] | 0.469 |
| log.sigma.1.5.mm.3D_gldm_SmallDependenceLowGrayLevelEmphasis (median [IQR]) | 0.00[0.00,0.00] | 0.00[0.00,0.00] | 0.00[0.00,0.00] | 0.152 |
| log.sigma.2.0.mm.3D_firstorder_10Percentile (median [IQR]) | -36.55[-73.78,-29.54] | -37.49[-79.24,-29.62] | -34.00[-62.84,-29.45] | 0.322 |
| log.sigma.2.0.mm.3D_firstorder_90Percentile (median [IQR]) | 1.25[-1.14,3.65] | 1.12[-1.09,3.60] | 1.37[-1.33,3.63] | 0.896 |
| log.sigma.2.0.mm.3D_firstorder_Energy (median [IQR]) | 23092483.43[6576038.53,92610727.35] | 31209092.27[7917914.82,106705204.10] | 13913951.32[3555376.13,59042485.36] | 0.025 |
| log.sigma.2.0.mm.3D_firstorder_Entropy (median [IQR]) | 1.63[1.32,2.07] | 1.65[1.32,2.10] | 1.57[1.32,1.98] | 0.417 |
| log.sigma.2.0.mm.3D_firstorder_InterquartileRange (median [IQR]) | 18.53[15.21,24.57] | 18.95[15.65,24.96] | 16.77[14.67,22.11] | 0.164 |
| log.sigma.2.0.mm.3D_firstorder_Kurtosis (median [IQR]) | 11.93[5.95,21.57] | 11.92[5.56,19.53] | 12.41[7.85,26.98] | 0.27 |
| log.sigma.2.0.mm.3D_firstorder_Maximum (median [IQR]) | 57.05[39.63,91.47] | 62.21[41.37,94.38] | 55.88[32.55,88.22] | 0.241 |
| log.sigma.2.0.mm.3D_firstorder_MeanAbsoluteDeviation (median [IQR]) | 15.16[10.77,25.83] | 16.01[10.86,26.99] | 14.63[10.59,21.56] | 0.588 |
| log.sigma.2.0.mm.3D_firstorder_Mean (median [IQR]) | -18.90[-29.11,-14.13] | -18.96[-29.28,-14.11] | -17.93[-25.20,-14.51] | 0.438 |
| log.sigma.2.0.mm.3D_firstorder_Median (median [IQR]) | -14.89[-19.30,-10.03] | -15.16[-19.50,-10.02] | -13.08[-18.31,-10.19] | 0.239 |
| log.sigma.2.0.mm.3D_firstorder_Minimum (median [IQR]) | -292.77[-350.61,-164.43] | -301.80[-355.08,-165.33] | -256.78[-322.09,-164.68] | 0.268 |
| log.sigma.2.0.mm.3D_firstorder_Range (median [IQR]) | 347.63[207.66,434.96] | 373.91[205.15,439.84] | 332.98[217.09,415.46] | 0.248 |
| log.sigma.2.0.mm.3D_firstorder_RobustMeanAbsoluteDeviation (median [IQR]) | 7.97[6.55,11.66] | 8.10[6.60,12.39] | 7.30[6.10,11.37] | 0.296 |
| log.sigma.2.0.mm.3D_firstorder_RootMeanSquared (median [IQR]) | 32.83[22.13,51.35] | 33.49[22.54,52.02] | 31.88[21.88,43.55] | 0.597 |
| log.sigma.2.0.mm.3D_firstorder_Skewness (median [IQR]) | -2.51[-3.51,-1.44] | -2.38[-3.37,-1.30] | -2.66[-4.18,-1.99] | 0.124 |
| log.sigma.2.0.mm.3D_firstorder_TotalEnergy (median [IQR]) | 49374679.26[13518242.07,187465451.59] | 61658139.76[17363668.76,216052910.10] | 28095080.08[7877468.13,163550510.31] | 0.021 |
| log.sigma.2.0.mm.3D_firstorder_Uniformity (median [IQR]) | 0.44[0.36,0.51] | 0.43[0.35,0.50] | 0.44[0.39,0.52] | 0.25 |
| log.sigma.2.0.mm.3D_firstorder_Variance (median [IQR]) | 735.33[255.46,1846.68] | 861.27[234.83,1935.69] | 687.20[305.23,1312.74] | 0.751 |
| log.sigma.2.0.mm.3D_glcm_Autocorrelation (median [IQR]) | 141.19[47.27,196.23] | 145.71[48.25,204.85] | 119.41[47.23,175.86] | 0.322 |
| log.sigma.2.0.mm.3D_glcm_ClusterProminence (median [IQR]) | 345.89[18.54,1285.21] | 366.46[12.98,1307.65] | 259.91[35.34,805.92] | 0.716 |
| log.sigma.2.0.mm.3D_glcm_ClusterShade (median [IQR]) | -27.79[-88.04,-2.07] | -29.88[-92.02,-1.50] | -21.38[-50.67,-4.42] | 0.699 |
| log.sigma.2.0.mm.3D_glcm_ClusterTendency (median [IQR]) | 3.74[1.27,9.10] | 4.30[1.17,9.60] | 3.53[1.69,6.10] | 0.679 |
| log.sigma.2.0.mm.3D_glcm_Contrast (median [IQR]) | 0.51[0.36,1.09] | 0.52[0.36,1.14] | 0.48[0.40,0.79] | 0.48 |
| log.sigma.2.0.mm.3D_glcm_Correlation (median [IQR]) | 0.69[0.53,0.78] | 0.70[0.52,0.78] | 0.67[0.59,0.79] | 0.74 |
| log.sigma.2.0.mm.3D_glcm_DifferenceAverage (median [IQR]) | 0.40[0.31,0.53] | 0.41[0.31,0.54] | 0.38[0.32,0.46] | 0.303 |
| log.sigma.2.0.mm.3D_glcm_DifferenceEntropy (median [IQR]) | 1.08[0.92,1.34] | 1.09[0.93,1.36] | 1.05[0.92,1.23] | 0.463 |
| log.sigma.2.0.mm.3D_glcm_DifferenceVariance (median [IQR]) | 0.36[0.25,0.76] | 0.37[0.25,0.80] | 0.35[0.26,0.61] | 0.594 |
| log.sigma.2.0.mm.3D_glcm_Id (median [IQR]) | 0.82[0.79,0.85] | 0.82[0.78,0.85] | 0.83[0.80,0.85] | 0.262 |
| log.sigma.2.0.mm.3D_glcm_Idm (median [IQR]) | 0.82[0.78,0.85] | 0.81[0.77,0.85] | 0.83[0.79,0.85] | 0.274 |
| log.sigma.2.0.mm.3D_glcm_Idmn (median [IQR]) | 1.00[0.99,1.00] | 1.00[0.99,1.00] | 1.00[0.99,1.00] | 0.842 |
| log.sigma.2.0.mm.3D_glcm_Idn (median [IQR]) | 0.97[0.96,0.98] | 0.97[0.96,0.98] | 0.97[0.96,0.98] | 0.597 |
| log.sigma.2.0.mm.3D_glcm_Imc1 (median [IQR]) | -0.26[-0.30,-0.22] | -0.26[-0.29,-0.22] | -0.26[-0.31,-0.23] | 0.666 |
| log.sigma.2.0.mm.3D_glcm_Imc2 (median [IQR]) | 0.69[0.58,0.78] | 0.70[0.58,0.78] | 0.68[0.60,0.80] | 0.925 |
| log.sigma.2.0.mm.3D_glcm_InverseVariance (median [IQR]) | 0.30[0.28,0.33] | 0.30[0.28,0.33] | 0.30[0.25,0.33] | 0.313 |
| log.sigma.2.0.mm.3D_glcm_JointAverage (median [IQR]) | 11.76[6.86,13.94] | 12.01[6.92,14.27] | 10.90[6.85,13.24] | 0.331 |
| log.sigma.2.0.mm.3D_glcm_JointEnergy (median [IQR]) | 0.28[0.21,0.35] | 0.27[0.20,0.34] | 0.31[0.24,0.36] | 0.144 |
| log.sigma.2.0.mm.3D_glcm_JointEntropy (median [IQR]) | 2.67[2.23,3.31] | 2.75[2.24,3.42] | 2.65[2.18,3.09] | 0.298 |
| log.sigma.2.0.mm.3D_glcm_MCC (median [IQR]) | 0.77[0.59,0.84] | 0.75[0.57,0.83] | 0.80[0.67,0.85] | 0.376 |
| log.sigma.2.0.mm.3D_glcm_MaximumProbability (median [IQR]) | 0.48[0.38,0.56] | 0.48[0.37,0.55] | 0.50[0.40,0.58] | 0.189 |
| log.sigma.2.0.mm.3D_glcm_SumAverage (median [IQR]) | 23.51[13.72,27.87] | 24.02[13.84,28.54] | 21.80[13.69,26.47] | 0.331 |
| log.sigma.2.0.mm.3D_glcm_SumEntropy (median [IQR]) | 2.22[1.86,2.68] | 2.24[1.86,2.74] | 2.15[1.84,2.54] | 0.292 |
| log.sigma.2.0.mm.3D_glcm_SumSquares (median [IQR]) | 1.06[0.40,2.51] | 1.26[0.39,2.73] | 0.99[0.54,1.72] | 0.652 |
| log.sigma.2.0.mm.3D_glrlm_GrayLevelNonUniformity (median [IQR]) | 3021.27[1614.06,5817.62] | 3445.43[1865.17,6119.69] | 1962.45[932.75,4161.87] | 0.01 |
| log.sigma.2.0.mm.3D_glrlm_GrayLevelNonUniformityNormalized (median [IQR]) | 0.33[0.24,0.40] | 0.32[0.23,0.40] | 0.34[0.26,0.40] | 0.284 |
| log.sigma.2.0.mm.3D_glrlm_GrayLevelVariance (median [IQR]) | 2.38[0.69,4.97] | 2.63[0.66,5.26] | 2.18[1.11,3.66] | 0.8 |
| log.sigma.2.0.mm.3D_glrlm_HighGrayLevelRunEmphasis (median [IQR]) | 131.83[46.90,184.21] | 139.98[47.22,192.82] | 115.40[44.85,168.85] | 0.327 |
| log.sigma.2.0.mm.3D_glrlm_LongRunEmphasis (median [IQR]) | 15.78[11.67,21.41] | 16.04[11.94,21.45] | 15.02[11.09,20.18] | 0.765 |
| log.sigma.2.0.mm.3D_glrlm_LongRunHighGrayLevelEmphasis (median [IQR]) | 1949.85[726.21,3105.75] | 2102.52[768.12,3296.10] | 1513.40[644.66,2727.18] | 0.376 |
| log.sigma.2.0.mm.3D_glrlm_LongRunLowGrayLevelEmphasis (median [IQR]) | 0.13[0.07,0.38] | 0.13[0.06,0.39] | 0.15[0.08,0.28] | 0.518 |
| log.sigma.2.0.mm.3D_glrlm_LowGrayLevelRunEmphasis (median [IQR]) | 0.01[0.01,0.03] | 0.01[0.01,0.02] | 0.02[0.01,0.03] | 0.198 |
| log.sigma.2.0.mm.3D_glrlm_RunEntropy (median [IQR]) | 4.18[3.80,4.40] | 4.21[3.83,4.43] | 4.04[3.69,4.35] | 0.106 |
| log.sigma.2.0.mm.3D_glrlm_RunLengthNonUniformity (median [IQR]) | 4217.55[2014.95,7222.60] | 4704.06[2397.30,8696.70] | 2303.80[1244.99,4887.58] | 0.002 |
| log.sigma.2.0.mm.3D_glrlm_RunLengthNonUniformityNormalized (median [IQR]) | 0.36[0.31,0.41] | 0.36[0.31,0.41] | 0.35[0.33,0.39] | 0.839 |
| log.sigma.2.0.mm.3D_glrlm_RunPercentage (median [IQR]) | 0.47[0.44,0.53] | 0.48[0.43,0.53] | 0.47[0.45,0.53] | 0.896 |
| log.sigma.2.0.mm.3D_glrlm_RunVariance (median [IQR]) | 7.68[5.71,11.04] | 7.73[5.67,11.05] | 7.48[5.79,10.78] | 0.758 |
| log.sigma.2.0.mm.3D_glrlm_ShortRunEmphasis (median [IQR]) | 0.60[0.55,0.65] | 0.60[0.54,0.65] | 0.60[0.56,0.64] | 0.962 |
| log.sigma.2.0.mm.3D_glrlm_ShortRunHighGrayLevelEmphasis (median [IQR]) | 79.44[26.02,107.97] | 80.91[25.48,114.88] | 64.72[26.94,96.21] | 0.298 |
| log.sigma.2.0.mm.3D_glrlm_ShortRunLowGrayLevelEmphasis (median [IQR]) | 0.01[0.00,0.02] | 0.01[0.00,0.01] | 0.01[0.01,0.02] | 0.162 |
| log.sigma.2.0.mm.3D_glszm_GrayLevelNonUniformity (median [IQR]) | 41.98[23.07,67.36] | 45.04[28.40,73.57] | 31.70[15.18,54.54] | 0.003 |
| log.sigma.2.0.mm.3D_glszm_GrayLevelNonUniformityNormalized (median [IQR]) | 0.15[0.10,0.30] | 0.15[0.10,0.31] | 0.15[0.11,0.26] | 0.689 |
| log.sigma.2.0.mm.3D_glszm_GrayLevelVariance (median [IQR]) | 9.07[2.86,12.77] | 9.85[2.55,13.07] | 8.26[4.20,11.85] | 0.471 |
| log.sigma.2.0.mm.3D_glszm_HighGrayLevelZoneEmphasis (median [IQR]) | 105.40[49.49,143.14] | 112.07[52.19,149.56] | 70.84[41.13,134.74] | 0.23 |
| log.sigma.2.0.mm.3D_glszm_LargeAreaEmphasis (median [IQR]) | 730749.40[258594.56,1585760.38] | 856229.62[295043.44,1756859.08] | 507338.79[190002.60,1046385.62] | 0.035 |
| log.sigma.2.0.mm.3D_glszm_LargeAreaHighGrayLevelEmphasis (median [IQR]) | 75891125.95[10984926.47,235867477.53] | 91905178.09[16598697.48,266604761.04] | 41656509.14[7106395.63,99775278.10] | 0.045 |
| log.sigma.2.0.mm.3D_glszm_LargeAreaLowGrayLevelEmphasis (median [IQR]) | 8751.77[2238.03,26466.19] | 10429.64[2361.66,26806.99] | 5271.32[1481.58,17963.46] | 0.192 |
| log.sigma.2.0.mm.3D_glszm_LowGrayLevelZoneEmphasis (median [IQR]) | 0.02[0.02,0.05] | 0.02[0.02,0.04] | 0.03[0.02,0.06] | 0.077 |
| log.sigma.2.0.mm.3D_glszm_SizeZoneNonUniformity (median [IQR]) | 35.92[16.68,88.54] | 40.17[19.16,95.15] | 23.81[10.46,64.73] | 0.026 |
| log.sigma.2.0.mm.3D_glszm_SizeZoneNonUniformityNormalized (median [IQR]) | 0.15[0.13,0.17] | 0.15[0.13,0.17] | 0.15[0.13,0.18] | 0.9 |
| log.sigma.2.0.mm.3D_glszm_SmallAreaEmphasis (median [IQR]) | 0.38[0.34,0.42] | 0.39[0.34,0.41] | 0.37[0.34,0.42] | 0.607 |
| log.sigma.2.0.mm.3D_glszm_SmallAreaHighGrayLevelEmphasis (median [IQR]) | 42.43[16.83,57.61] | 47.40[17.30,58.95] | 31.20[14.47,55.44] | 0.245 |
| log.sigma.2.0.mm.3D_glszm_SmallAreaLowGrayLevelEmphasis (median [IQR]) | 0.01[0.01,0.01] | 0.01[0.01,0.01] | 0.01[0.01,0.02] | 0.15 |
| log.sigma.2.0.mm.3D_glszm_ZoneEntropy (median [IQR]) | 6.03[5.16,6.68] | 6.13[5.22,6.77] | 5.82[5.02,6.29] | 0.066 |
| log.sigma.2.0.mm.3D_glszm_ZonePercentage (median [IQR]) | 0.01[0.01,0.02] | 0.01[0.01,0.02] | 0.01[0.01,0.02] | 0.343 |
| log.sigma.2.0.mm.3D_glszm_ZoneVariance (median [IQR]) | 711382.48[250992.45,1566044.75] | 852796.81[293230.89,1745287.81] | 498612.56[185496.55,1025241.27] | 0.034 |
| log.sigma.2.0.mm.3D_ngtdm_Busyness (median [IQR]) | 11.69[4.83,21.09] | 12.72[5.70,23.05] | 5.82[3.40,18.20] | 0.029 |
| log.sigma.2.0.mm.3D_ngtdm_Coarseness (median [IQR]) | 0.00[0.00,0.00] | 0.00[0.00,0.00] | 0.00[0.00,0.00] | 0.003 |
| log.sigma.2.0.mm.3D_ngtdm_Complexity (median [IQR]) | 42.49[12.24,75.78] | 46.63[13.04,77.87] | 32.71[11.53,55.96] | 0.198 |
| log.sigma.2.0.mm.3D_ngtdm_Contrast (median [IQR]) | 0.01[0.00,0.01] | 0.01[0.00,0.01] | 0.01[0.00,0.01] | 0.929 |
| log.sigma.2.0.mm.3D_ngtdm_Strength (median [IQR]) | 0.09[0.04,0.23] | 0.07[0.04,0.19] | 0.16[0.05,0.29] | 0.036 |
| log.sigma.2.0.mm.3D_gldm_DependenceEntropy (median [IQR]) | 5.84[5.58,6.25] | 5.90[5.60,6.26] | 5.82[5.54,6.11] | 0.19 |
| log.sigma.2.0.mm.3D_gldm_DependenceNonUniformity (median [IQR]) | 1001.42 [474.37, 2012.16] | 1275.94 [571.58, 2256.01] | 520.73 [280.91, 1053.79] | <0.001 |
| log.sigma.2.0.mm.3D_gldm_DependenceNonUniformityNormalized (median [IQR]) | 0.05[0.05,0.05] | 0.05[0.05,0.05] | 0.05[0.04,0.05] | 0.782 |
| log.sigma.2.0.mm.3D_gldm_DependenceVariance (median [IQR]) | 45.82[41.01,50.01] | 45.95[41.39,50.34] | 45.20[40.90,49.77] | 0.554 |
| log.sigma.2.0.mm.3D_gldm_GrayLevelNonUniformity (median [IQR]) | 8485.07[4268.22,17073.68] | 9725.56[4558.08,18015.33] | 5796.73[2510.34,11468.13] | 0.013 |
| log.sigma.2.0.mm.3D_gldm_GrayLevelVariance (median [IQR]) | 1.24[0.46,3.03] | 1.49[0.45,3.19] | 1.17[0.57,2.14] | 0.74 |
| log.sigma.2.0.mm.3D_gldm_HighGrayLevelEmphasis (median [IQR]) | 141.03[47.41,194.29] | 145.34[48.29,202.81] | 118.40[46.70,174.19] | 0.318 |
| log.sigma.2.0.mm.3D_gldm_LargeDependenceEmphasis (median [IQR]) | 258.36[223.12,289.45] | 258.65[222.86,298.79] | 257.76[227.79,280.88] | 0.81 |
| log.sigma.2.0.mm.3D_gldm_LargeDependenceHighGrayLevelEmphasis (median [IQR]) | 35324.58[12714.68,55446.80] | 37535.66[13070.76,56257.81] | 24810.83[12230.17,52265.58] | 0.362 |
| log.sigma.2.0.mm.3D_gldm_LargeDependenceLowGrayLevelEmphasis (median [IQR]) | 1.91[1.24,6.00] | 1.84[1.08,6.05] | 2.24[1.38,5.92] | 0.399 |
| log.sigma.2.0.mm.3D_gldm_LowGrayLevelEmphasis (median [IQR]) | 0.01[0.01,0.02] | 0.01[0.01,0.02] | 0.01[0.01,0.02] | 0.252 |
| log.sigma.2.0.mm.3D_gldm_SmallDependenceEmphasis (median [IQR]) | 0.02[0.01,0.02] | 0.02[0.01,0.02] | 0.02[0.01,0.02] | 0.636 |
| log.sigma.2.0.mm.3D_gldm_SmallDependenceHighGrayLevelEmphasis (median [IQR]) | 2.15[0.73,3.74] | 2.21[0.68,3.86] | 1.91[0.76,3.26] | 0.48 |
| log.sigma.2.0.mm.3D_gldm_SmallDependenceLowGrayLevelEmphasis (median [IQR]) | 0.00[0.00,0.00] | 0.00[0.00,0.00] | 0.00[0.00,0.00] | 0.135 |
| log.sigma.2.5.mm.3D_firstorder_10Percentile (median [IQR]) | -41.94[-90.32,-34.43] | -43.39[-99.86,-34.82] | -38.55[-78.30,-33.00] | 0.241 |
| log.sigma.2.5.mm.3D_firstorder_90Percentile (median [IQR]) | -1.44[-4.48,2.07] | -1.56[-4.29,2.11] | -0.47[-4.58,1.83] | 0.585 |
| log.sigma.2.5.mm.3D_firstorder_Energy (median [IQR]) | 30995039.28[9155559.75,117944163.96] | 40451276.89[10670241.13,142042659.48] | 19378276.05[4978754.51,67138871.51] | 0.021 |
| log.sigma.2.5.mm.3D_firstorder_Entropy (median [IQR]) | 1.71[1.37,2.25] | 1.72[1.38,2.31] | 1.68[1.36,2.14] | 0.336 |
| log.sigma.2.5.mm.3D_firstorder_InterquartileRange (median [IQR]) | 19.68[16.06,28.68] | 20.21[16.54,30.04] | 17.96[15.39,28.31] | 0.133 |
| log.sigma.2.5.mm.3D_firstorder_Kurtosis (median [IQR]) | 9.57[5.11,17.71] | 9.59[5.05,16.01] | 9.56[6.20,19.48] | 0.409 |
| log.sigma.2.5.mm.3D_firstorder_Maximum (median [IQR]) | 65.06[41.71,107.26] | 70.14[43.87,107.51] | 57.71[35.49,103.28] | 0.284 |
| log.sigma.2.5.mm.3D_firstorder_MeanAbsoluteDeviation (median [IQR]) | 16.88[11.49,29.80] | 17.20[11.53,31.68] | 16.12[11.45,25.78] | 0.471 |
| log.sigma.2.5.mm.3D_firstorder_Mean (median [IQR]) | -22.83[-36.74,-17.50] | -22.88[-37.54,-17.55] | -22.67[-30.39,-16.83] | 0.288 |
| log.sigma.2.5.mm.3D_firstorder_Median (median [IQR]) | -18.40[-24.25,-12.93] | -19.06[-24.64,-12.87] | -16.77[-22.16,-13.40] | 0.135 |
| log.sigma.2.5.mm.3D_firstorder_Minimum (median [IQR]) | -270.26[-337.23,-161.16] | -285.17[-349.28,-162.64] | -241.86[-301.64,-153.05] | 0.254 |
| log.sigma.2.5.mm.3D_firstorder_Range (median [IQR]) | 350.20[205.43,441.60] | 370.88[208.80,444.07] | 325.67[205.94,397.68] | 0.292 |
| log.sigma.2.5.mm.3D_firstorder_RobustMeanAbsoluteDeviation (median [IQR]) | 8.75[6.82,14.63] | 8.89[7.15,15.68] | 8.06[6.65,12.77] | 0.2 |
| log.sigma.2.5.mm.3D_firstorder_RootMeanSquared (median [IQR]) | 37.98[25.66,59.37] | 38.92[25.99,60.80] | 34.76[23.89,50.38] | 0.497 |
| log.sigma.2.5.mm.3D_firstorder_Skewness (median [IQR]) | -2.16[-3.08,-1.31] | -2.00[-2.91,-1.26] | -2.27[-3.50,-1.65] | 0.178 |
| log.sigma.2.5.mm.3D_firstorder_TotalEnergy (median [IQR]) | 65152732.16[18880990.70,255918326.21] | 78626595.09[23070214.52,297901733.48] | 34011790.11[10227112.98,194283465.63] | 0.018 |
| log.sigma.2.5.mm.3D_firstorder_Uniformity (median [IQR]) | 0.41[0.32,0.47] | 0.40[0.30,0.47] | 0.42[0.34,0.51] | 0.246 |
| log.sigma.2.5.mm.3D_firstorder_Variance (median [IQR]) | 882.97[299.02,2183.56] | 982.14[285.74,2468.54] | 824.51[329.45,1625.05] | 0.636 |
| log.sigma.2.5.mm.3D_glcm_Autocorrelation (median [IQR]) | 122.01[44.14,180.34] | 123.41[44.09,189.34] | 101.00[44.85,146.49] | 0.35 |
| log.sigma.2.5.mm.3D_glcm_ClusterProminence (median [IQR]) | 430.47[33.01,1662.21] | 469.84[24.17,1776.10] | 319.11[47.52,983.93] | 0.73 |
| log.sigma.2.5.mm.3D_glcm_ClusterShade (median [IQR]) | -32.01[-107.34,-2.97] | -40.27[-113.96,-2.61] | -27.73[-67.85,-5.21] | 0.744 |
| log.sigma.2.5.mm.3D_glcm_ClusterTendency (median [IQR]) | 4.94[1.50,11.90] | 5.39[1.49,12.85] | 4.21[1.94,7.27] | 0.585 |
| log.sigma.2.5.mm.3D_glcm_Contrast (median [IQR]) | 0.49[0.35,1.04] | 0.51[0.35,1.14] | 0.45[0.37,0.82] | 0.417 |
| log.sigma.2.5.mm.3D_glcm_Correlation (median [IQR]) | 0.77[0.62,0.84] | 0.77[0.59,0.83] | 0.74[0.65,0.85] | 0.878 |
| log.sigma.2.5.mm.3D_glcm_DifferenceAverage (median [IQR]) | 0.38[0.30,0.55] | 0.39[0.30,0.57] | 0.37[0.30,0.47] | 0.329 |
| log.sigma.2.5.mm.3D_glcm_DifferenceEntropy (median [IQR]) | 1.05[0.91,1.36] | 1.05[0.91,1.39] | 1.04[0.92,1.27] | 0.357 |
| log.sigma.2.5.mm.3D_glcm_DifferenceVariance (median [IQR]) | 0.33[0.24,0.70] | 0.34[0.24,0.77] | 0.32[0.24,0.58] | 0.376 |
| log.sigma.2.5.mm.3D_glcm_Id (median [IQR]) | 0.83[0.78,0.86] | 0.83[0.78,0.86] | 0.83[0.81,0.86] | 0.336 |
| log.sigma.2.5.mm.3D_glcm_Idm (median [IQR]) | 0.82[0.77,0.86] | 0.82[0.77,0.85] | 0.82[0.79,0.86] | 0.329 |
| log.sigma.2.5.mm.3D_glcm_Idmn (median [IQR]) | 1.00[0.99,1.00] | 1.00[0.99,1.00] | 1.00[0.99,1.00] | 0.885 |
| log.sigma.2.5.mm.3D_glcm_Idn (median [IQR]) | 0.97[0.96,0.98] | 0.97[0.96,0.98] | 0.97[0.96,0.98] | 0.672 |
| log.sigma.2.5.mm.3D_glcm_Imc1 (median [IQR]) | -0.31[-0.35,-0.27] | -0.31[-0.35,-0.27] | -0.32[-0.37,-0.27] | 0.72 |
| log.sigma.2.5.mm.3D_glcm_Imc2 (median [IQR]) | 0.78[0.66,0.85] | 0.79[0.66,0.85] | 0.77[0.66,0.85] | 0.775 |
| log.sigma.2.5.mm.3D_glcm_InverseVariance (median [IQR]) | 0.29[0.26,0.33] | 0.29[0.26,0.33] | 0.27[0.26,0.33] | 0.343 |
| log.sigma.2.5.mm.3D_glcm_JointAverage (median [IQR]) | 10.95[6.62,13.35] | 11.03[6.62,13.66] | 10.03[6.66,11.81] | 0.34 |
| log.sigma.2.5.mm.3D_glcm_JointEnergy (median [IQR]) | 0.26[0.19,0.32] | 0.26[0.18,0.31] | 0.27[0.22,0.35] | 0.178 |
| log.sigma.2.5.mm.3D_glcm_JointEntropy (median [IQR]) | 2.77[2.29,3.53] | 2.79[2.32,3.66] | 2.65[2.26,3.46] | 0.274 |
| log.sigma.2.5.mm.3D_glcm_MCC (median [IQR]) | 0.82[0.68,0.88] | 0.81[0.67,0.87] | 0.84[0.75,0.90] | 0.46 |
| log.sigma.2.5.mm.3D_glcm_MaximumProbability (median [IQR]) | 0.44[0.34,0.54] | 0.44[0.34,0.52] | 0.45[0.34,0.57] | 0.268 |
| log.sigma.2.5.mm.3D_glcm_SumAverage (median [IQR]) | 21.89[13.25,26.71] | 22.06[13.24,27.33] | 20.06[13.31,23.62] | 0.34 |
| log.sigma.2.5.mm.3D_glcm_SumEntropy (median [IQR]) | 2.31[1.91,2.87] | 2.33[1.95,2.98] | 2.23[1.88,2.72] | 0.254 |
| log.sigma.2.5.mm.3D_glcm_SumSquares (median [IQR]) | 1.32[0.46,3.36] | 1.46[0.46,3.44] | 1.17[0.60,2.06] | 0.582 |
| log.sigma.2.5.mm.3D_glrlm_GrayLevelNonUniformity (median [IQR]) | 2791.25[1497.60,5013.87] | 3038.91[1678.18,5716.32] | 1846.70[881.79,3599.08] | 0.01 |
| log.sigma.2.5.mm.3D_glrlm_GrayLevelNonUniformityNormalized (median [IQR]) | 0.30[0.21,0.39] | 0.30[0.19,0.39] | 0.32[0.23,0.40] | 0.228 |
| log.sigma.2.5.mm.3D_glrlm_GrayLevelVariance (median [IQR]) | 2.79[0.86,5.62] | 3.09[0.85,6.35] | 2.35[1.20,4.66] | 0.772 |
| log.sigma.2.5.mm.3D_glrlm_HighGrayLevelRunEmphasis (median [IQR]) | 114.37[44.14,168.72] | 118.11[44.38,177.44] | 99.04[43.02,136.34] | 0.324 |
| log.sigma.2.5.mm.3D_glrlm_LongRunEmphasis (median [IQR]) | 16.80[12.09,23.55] | 16.54[11.90,24.23] | 17.82[12.29,21.21] | 0.882 |
| log.sigma.2.5.mm.3D_glrlm_LongRunHighGrayLevelEmphasis (median [IQR]) | 1802.37[756.21,2965.82] | 1820.30[788.90,3252.45] | 1562.02[567.79,2721.63] | 0.449 |
| log.sigma.2.5.mm.3D_glrlm_LongRunLowGrayLevelEmphasis (median [IQR]) | 0.17[0.09,0.45] | 0.16[0.08,0.43] | 0.20[0.11,0.49] | 0.414 |
| log.sigma.2.5.mm.3D_glrlm_LowGrayLevelRunEmphasis (median [IQR]) | 0.01[0.01,0.03] | 0.01[0.01,0.03] | 0.02[0.01,0.03] | 0.122 |
| log.sigma.2.5.mm.3D_glrlm_RunEntropy (median [IQR]) | 4.38[3.90,4.57] | 4.41[3.95,4.60] | 4.20[3.76,4.51] | 0.095 |
| log.sigma.2.5.mm.3D_glrlm_RunLengthNonUniformity (median [IQR]) | 3925.48[2043.18,7000.39] | 4506.45[2400.61,8732.72] | 2575.84[1158.33,4561.35] | 0.001 |
| log.sigma.2.5.mm.3D_glrlm_RunLengthNonUniformityNormalized (median [IQR]) | 0.36[0.30,0.41] | 0.36[0.30,0.41] | 0.36[0.30,0.41] | 0.807 |
| log.sigma.2.5.mm.3D_glrlm_RunPercentage (median [IQR]) | 0.47[0.42,0.53] | 0.47[0.41,0.53] | 0.46[0.43,0.52] | 0.821 |
| log.sigma.2.5.mm.3D_glrlm_RunVariance (median [IQR]) | 8.51[5.82,12.66] | 8.34[5.63,13.12] | 8.74[6.15,10.85] | 0.936 |
| log.sigma.2.5.mm.3D_glrlm_ShortRunEmphasis (median [IQR]) | 0.59[0.54,0.65] | 0.59[0.54,0.65] | 0.59[0.54,0.64] | 0.846 |
| log.sigma.2.5.mm.3D_glrlm_ShortRunHighGrayLevelEmphasis (median [IQR]) | 64.24[24.85,99.60] | 68.01[25.16,103.91] | 55.69[24.54,80.23] | 0.266 |
| log.sigma.2.5.mm.3D_glrlm_ShortRunLowGrayLevelEmphasis (median [IQR]) | 0.01[0.01,0.02] | 0.01[0.01,0.02] | 0.01[0.01,0.02] | 0.099 |
| log.sigma.2.5.mm.3D_glszm_GrayLevelNonUniformity (median [IQR]) | 36.12[19.19,58.56] | 40.60[26.20,61.96] | 25.73[11.60,46.04] | 0.004 |
| log.sigma.2.5.mm.3D_glszm_GrayLevelNonUniformityNormalized (median [IQR]) | 0.14[0.10,0.31] | 0.14[0.10,0.31] | 0.14[0.11,0.28] | 0.692 |
| log.sigma.2.5.mm.3D_glszm_GrayLevelVariance (median [IQR]) | 8.68[2.79,12.56] | 8.69[2.63,13.07] | 7.98[3.45,11.42] | 0.48 |
| log.sigma.2.5.mm.3D_glszm_HighGrayLevelZoneEmphasis (median [IQR]) | 102.52[50.50,138.48] | 105.15[52.74,140.82] | 76.21[41.71,119.81] | 0.153 |
| log.sigma.2.5.mm.3D_glszm_LargeAreaEmphasis (median [IQR]) | 787269.57[313890.92,1702579.03] | 884684.38[337955.46,2010631.32] | 531684.44[215379.87,1295995.21] | 0.057 |
| log.sigma.2.5.mm.3D_glszm_LargeAreaHighGrayLevelEmphasis (median [IQR]) | 71660418.78[13264930.96,233886707.13] | 92089723.44[18996795.43,265184416.73] | 33755055.65[7670248.99,122317380.49] | 0.056 |
| log.sigma.2.5.mm.3D_glszm_LargeAreaLowGrayLevelEmphasis (median [IQR]) | 9354.87[2475.72,31231.05] | 10250.18[2947.95,30244.68] | 7229.14[1952.66,31375.97] | 0.296 |
| log.sigma.2.5.mm.3D_glszm_LowGrayLevelZoneEmphasis (median [IQR]) | 0.03[0.02,0.05] | 0.02[0.01,0.04] | 0.04[0.02,0.07] | 0.084 |
| log.sigma.2.5.mm.3D_glszm_SizeZoneNonUniformity (median [IQR]) | 29.45[14.78,87.97] | 37.61[17.37,92.58] | 19.13[7.29,60.26] | 0.023 |
| log.sigma.2.5.mm.3D_glszm_SizeZoneNonUniformityNormalized (median [IQR]) | 0.16[0.13,0.19] | 0.16[0.13,0.19] | 0.16[0.13,0.19] | 0.588 |
| log.sigma.2.5.mm.3D_glszm_SmallAreaEmphasis (median [IQR]) | 0.40[0.35,0.45] | 0.40[0.35,0.45] | 0.39[0.34,0.44] | 0.379 |
| log.sigma.2.5.mm.3D_glszm_SmallAreaHighGrayLevelEmphasis (median [IQR]) | 41.94[18.78,66.55] | 47.52[19.68,66.83] | 31.93[17.80,56.07] | 0.172 |
| log.sigma.2.5.mm.3D_glszm_SmallAreaLowGrayLevelEmphasis (median [IQR]) | 0.01[0.01,0.01] | 0.01[0.01,0.01] | 0.01[0.01,0.02] | 0.296 |
| log.sigma.2.5.mm.3D_glszm_ZoneEntropy (median [IQR]) | 5.93[5.12,6.44] | 6.02[5.11,6.51] | 5.69[5.17,6.11] | 0.064 |
| log.sigma.2.5.mm.3D_glszm_ZonePercentage (median [IQR]) | 0.01[0.01,0.01] | 0.01[0.01,0.01] | 0.01[0.01,0.02] | 0.524 |
| log.sigma.2.5.mm.3D_glszm_ZoneVariance (median [IQR]) | 769898.68[308642.57,1690207.27] | 879555.69[329363.90,1985015.85] | 514685.21[212243.87,1257031.27] | 0.058 |
| log.sigma.2.5.mm.3D_ngtdm_Busyness (median [IQR]) | 10.37[4.51,20.28] | 12.41[5.30,20.86] | 6.00[3.15,19.43] | 0.059 |
| log.sigma.2.5.mm.3D_ngtdm_Coarseness (median [IQR]) | 0.00[0.00,0.00] | 0.00[0.00,0.00] | 0.00[0.00,0.00] | 0.003 |
| log.sigma.2.5.mm.3D_ngtdm_Complexity (median [IQR]) | 33.61[11.46,69.55] | 42.96[11.85,77.73] | 29.01[8.65,55.12] | 0.183 |
| log.sigma.2.5.mm.3D_ngtdm_Contrast (median [IQR]) | 0.01[0.00,0.01] | 0.01[0.00,0.01] | 0.01[0.00,0.01] | 0.907 |
| log.sigma.2.5.mm.3D_ngtdm_Strength (median [IQR]) | 0.08[0.04,0.21] | 0.07[0.04,0.16] | 0.15[0.05,0.31] | 0.06 |
| log.sigma.2.5.mm.3D_gldm_DependenceEntropy (median [IQR]) | 5.91[5.61,6.40] | 5.94[5.66,6.48] | 5.84[5.56,6.23] | 0.138 |
| log.sigma.2.5.mm.3D_gldm_DependenceNonUniformity (median [IQR]) | 1172.28[529.84,2243.42] | 1343.85[607.69,2470.31] | 685.46[304.44,1352.49] | 0.005 |
| log.sigma.2.5.mm.3D_gldm_DependenceNonUniformityNormalized (median [IQR]) | 0.05[0.05,0.06] | 0.05[0.05,0.06] | 0.05[0.05,0.06] | 0.703 |
| log.sigma.2.5.mm.3D_gldm_DependenceVariance (median [IQR]) | 46.63[41.21,51.14] | 46.45[41.14,51.07] | 47.30[41.37,51.55] | 0.907 |
| log.sigma.2.5.mm.3D_gldm_GrayLevelNonUniformity (median [IQR]) | 7440.85[3858.12,15942.77] | 9441.75[4344.56,16664.77] | 5712.75[2552.95,11777.84] | 0.028 |
| log.sigma.2.5.mm.3D_gldm_GrayLevelVariance (median [IQR]) | 1.53[0.55,3.56] | 1.67[0.52,4.01] | 1.38[0.63,2.65] | 0.649 |
| log.sigma.2.5.mm.3D_gldm_HighGrayLevelEmphasis (median [IQR]) | 121.28[44.61,179.95] | 124.21[44.59,188.46] | 100.65[44.70,145.54] | 0.334 |
| log.sigma.2.5.mm.3D_gldm_LargeDependenceEmphasis (median [IQR]) | 264.84[218.31,306.79] | 258.68[218.30,308.38] | 274.57[225.25,301.89] | 0.842 |
| log.sigma.2.5.mm.3D_gldm_LargeDependenceHighGrayLevelEmphasis (median [IQR]) | 30131.54[11062.05,47806.59] | 32036.15[11917.59,47897.99] | 26713.11[10146.83,43158.93] | 0.35 |
| log.sigma.2.5.mm.3D_gldm_LargeDependenceLowGrayLevelEmphasis (median [IQR]) | 2.27[1.35,6.15] | 2.06[1.22,6.09] | 2.46[1.70,6.54] | 0.3 |
| log.sigma.2.5.mm.3D_gldm_LowGrayLevelEmphasis (median [IQR]) | 0.01[0.01,0.03] | 0.01[0.01,0.03] | 0.01[0.01,0.03] | 0.192 |
| log.sigma.2.5.mm.3D_gldm_SmallDependenceEmphasis (median [IQR]) | 0.02[0.01,0.02] | 0.02[0.01,0.02] | 0.02[0.01,0.02] | 0.775 |
| log.sigma.2.5.mm.3D_gldm_SmallDependenceHighGrayLevelEmphasis (median [IQR]) | 1.68[0.70,3.55] | 2.04[0.68,3.65] | 1.53[0.71,2.59] | 0.381 |
| log.sigma.2.5.mm.3D_gldm_SmallDependenceLowGrayLevelEmphasis (median [IQR]) | 0.00[0.00,0.00] | 0.00[0.00,0.00] | 0.00[0.00,0.00] | 0.123 |
| log.sigma.3.0.mm.3D_firstorder_10Percentile (median [IQR]) | -50.86[-110.09,-37.04] | -55.50[-117.44,-38.87] | -47.09[-83.49,-35.60] | 0.178 |
| log.sigma.3.0.mm.3D_firstorder_90Percentile (median [IQR]) | -3.17[-7.01,0.85] | -3.44[-6.92,0.77] | -1.39[-7.75,0.89] | 0.364 |
| log.sigma.3.0.mm.3D_firstorder_Energy (median [IQR]) | 37552468.94[11018690.59,143232854.23] | 50259270.57[14432714.32,180684215.29] | 23083298.21[5999730.51,71996135.19] | 0.013 |
| log.sigma.3.0.mm.3D_firstorder_Entropy (median [IQR]) | 1.87[1.45,2.45] | 1.87[1.45,2.49] | 1.84[1.47,2.28] | 0.278 |
| log.sigma.3.0.mm.3D_firstorder_InterquartileRange (median [IQR]) | 21.72[17.51,36.25] | 22.32[18.15,39.52] | 19.32[16.48,31.01] | 0.105 |
| log.sigma.3.0.mm.3D_firstorder_Kurtosis (median [IQR]) | 8.13[4.36,14.16] | 8.23[4.31,13.69] | 7.59[4.98,14.89] | 0.503 |
| log.sigma.3.0.mm.3D_firstorder_Maximum (median [IQR]) | 79.44[39.49,130.68] | 80.16[42.65,131.77] | 65.14[34.17,122.14] | 0.327 |
| log.sigma.3.0.mm.3D_firstorder_MeanAbsoluteDeviation (median [IQR]) | 19.69[12.55,33.67] | 19.73[12.63,35.79] | 18.20[12.10,27.83] | 0.386 |
| log.sigma.3.0.mm.3D_firstorder_Mean (median [IQR]) | -27.10[-42.92,-20.03] | -27.34[-44.88,-20.46] | -26.67[-35.91,-18.85] | 0.187 |
| log.sigma.3.0.mm.3D_firstorder_Median (median [IQR]) | -21.87[-28.92,-15.55] | -22.53[-30.21,-15.28] | -19.66[-24.63,-15.72] | 0.089 |
| log.sigma.3.0.mm.3D_firstorder_Minimum (median [IQR]) | -253.22[-323.68,-167.06] | -269.24[-328.71,-174.33] | -227.99[-304.20,-144.83] | 0.216 |
| log.sigma.3.0.mm.3D_firstorder_Range (median [IQR]) | 353.21[204.96,449.07] | 369.61[217.27,459.07] | 330.04[202.19,389.47] | 0.167 |
| log.sigma.3.0.mm.3D_firstorder_RobustMeanAbsoluteDeviation (median [IQR]) | 9.38[7.43,18.37] | 9.47[7.80,18.88] | 8.33[6.94,14.76] | 0.144 |
| log.sigma.3.0.mm.3D_firstorder_RootMeanSquared (median [IQR]) | 41.37[27.46,65.26] | 42.68[27.86,68.34] | 38.67[25.64,53.71] | 0.35 |
| log.sigma.3.0.mm.3D_firstorder_Skewness (median [IQR]) | -1.82[-2.78,-0.98] | -1.82[-2.64,-0.98] | -1.95[-3.08,-1.14] | 0.32 |
| log.sigma.3.0.mm.3D_firstorder_TotalEnergy (median [IQR]) | 84810034.41[22336879.15,312562100.67] | 102769349.56[28881964.35,375985638.61] | 39491003.44[14006247.33,211347582.58] | 0.013 |
| log.sigma.3.0.mm.3D_firstorder_Uniformity (median [IQR]) | 0.39[0.27,0.45] | 0.38[0.26,0.44] | 0.41[0.31,0.47] | 0.184 |
| log.sigma.3.0.mm.3D_firstorder_Variance (median [IQR]) | 1002.39[331.90,2439.97] | 1211.59[337.29,2637.45] | 773.07[334.73,1846.72] | 0.572 |
| log.sigma.3.0.mm.3D_glcm_Autocorrelation (median [IQR]) | 99.04[41.92,142.46] | 112.40[50.16,155.45] | 85.19[36.35,137.89] | 0.276 |
| log.sigma.3.0.mm.3D_glcm_ClusterProminence (median [IQR]) | 426.62[32.91,1709.89] | 469.31[31.47,1794.73] | 359.35[37.98,1009.46] | 0.633 |
| log.sigma.3.0.mm.3D_glcm_ClusterShade (median [IQR]) | -33.02[-106.03,-3.43] | -36.55[-119.88,-2.80] | -25.43[-60.38,-4.56] | 0.572 |
| log.sigma.3.0.mm.3D_glcm_ClusterTendency (median [IQR]) | 5.64[1.78,13.51] | 6.28[1.78,14.66] | 4.40[1.87,9.09] | 0.521 |
| log.sigma.3.0.mm.3D_glcm_Contrast (median [IQR]) | 0.51[0.35,1.03] | 0.54[0.34,1.11] | 0.47[0.35,0.82] | 0.329 |
| log.sigma.3.0.mm.3D_glcm_Correlation (median [IQR]) | 0.79[0.65,0.86] | 0.80[0.65,0.86] | 0.79[0.66,0.86] | 0.991 |
| log.sigma.3.0.mm.3D_glcm_DifferenceAverage (median [IQR]) | 0.38[0.30,0.56] | 0.38[0.30,0.58] | 0.38[0.28,0.46] | 0.298 |
| log.sigma.3.0.mm.3D_glcm_DifferenceEntropy (median [IQR]) | 1.06[0.91,1.37] | 1.07[0.91,1.40] | 1.05[0.91,1.26] | 0.288 |
| log.sigma.3.0.mm.3D_glcm_DifferenceVariance (median [IQR]) | 0.34[0.24,0.68] | 0.36[0.24,0.72] | 0.30[0.25,0.56] | 0.309 |
| log.sigma.3.0.mm.3D_glcm_Id (median [IQR]) | 0.83[0.78,0.86] | 0.83[0.77,0.86] | 0.82[0.80,0.86] | 0.362 |
| log.sigma.3.0.mm.3D_glcm_Idm (median [IQR]) | 0.82[0.76,0.86] | 0.82[0.76,0.85] | 0.82[0.80,0.86] | 0.364 |
| log.sigma.3.0.mm.3D_glcm_Idmn (median [IQR]) | 1.00[0.99,1.00] | 1.00[0.99,1.00] | 1.00[0.99,1.00] | 0.914 |
| log.sigma.3.0.mm.3D_glcm_Idn (median [IQR]) | 0.97[0.96,0.98] | 0.97[0.96,0.98] | 0.97[0.96,0.98] | 0.73 |
| log.sigma.3.0.mm.3D_glcm_Imc1 (median [IQR]) | -0.34[-0.39,-0.29] | -0.35[-0.39,-0.29] | -0.33[-0.40,-0.29] | 0.998 |
| log.sigma.3.0.mm.3D_glcm_Imc2 (median [IQR]) | 0.83[0.70,0.88] | 0.84[0.70,0.88] | 0.81[0.67,0.86] | 0.486 |
| log.sigma.3.0.mm.3D_glcm_InverseVariance (median [IQR]) | 0.29[0.25,0.34] | 0.30[0.26,0.34] | 0.28[0.25,0.34] | 0.447 |
| log.sigma.3.0.mm.3D_glcm_JointAverage (median [IQR]) | 9.87[6.42,11.89] | 10.54[7.07,12.36] | 9.15[6.00,11.56] | 0.256 |
| log.sigma.3.0.mm.3D_glcm_JointEnergy (median [IQR]) | 0.25[0.16,0.30] | 0.25[0.15,0.30] | 0.26[0.20,0.31] | 0.167 |
| log.sigma.3.0.mm.3D_glcm_JointEntropy (median [IQR]) | 2.83[2.36,3.78] | 2.84[2.39,4.00] | 2.82[2.29,3.60] | 0.223 |
| log.sigma.3.0.mm.3D_glcm_MCC (median [IQR]) | 0.85[0.73,0.90] | 0.84[0.73,0.90] | 0.85[0.75,0.91] | 0.557 |
| log.sigma.3.0.mm.3D_glcm_MaximumProbability (median [IQR]) | 0.40[0.31,0.49] | 0.39[0.30,0.48] | 0.43[0.34,0.54] | 0.161 |
| log.sigma.3.0.mm.3D_glcm_SumAverage (median [IQR]) | 19.75[12.83,23.78] | 21.08[14.13,24.72] | 18.30[12.01,23.12] | 0.256 |
| log.sigma.3.0.mm.3D_glcm_SumEntropy (median [IQR]) | 2.43[2.02,3.12] | 2.43[2.04,3.21] | 2.36[1.91,2.85] | 0.216 |
| log.sigma.3.0.mm.3D_glcm_SumSquares (median [IQR]) | 1.50[0.53,3.73] | 1.79[0.53,3.91] | 1.23[0.58,2.45] | 0.518 |
| log.sigma.3.0.mm.3D_glrlm_GrayLevelNonUniformity (median [IQR]) | 2582.89[1389.38,4625.29] | 2836.40[1605.81,5106.40] | 1830.28[806.15,3218.65] | 0.012 |
| log.sigma.3.0.mm.3D_glrlm_GrayLevelNonUniformityNormalized (median [IQR]) | 0.27[0.19,0.37] | 0.27[0.17,0.36] | 0.31[0.21,0.39] | 0.206 |
| log.sigma.3.0.mm.3D_glrlm_GrayLevelVariance (median [IQR]) | 3.04[1.03,6.19] | 3.28[1.03,6.82] | 2.50[1.14,4.66] | 0.613 |
| log.sigma.3.0.mm.3D_glrlm_HighGrayLevelRunEmphasis (median [IQR]) | 96.02[41.38,136.01] | 103.41[51.93,144.38] | 82.69[36.84,124.47] | 0.227 |
| log.sigma.3.0.mm.3D_glrlm_LongRunEmphasis (median [IQR]) | 17.02[11.60,24.86] | 16.60[11.61,25.68] | 17.98[11.45,22.75] | 0.807 |
| log.sigma.3.0.mm.3D_glrlm_LongRunHighGrayLevelEmphasis (median [IQR]) | 1650.53[661.48,2650.17] | 1681.14[735.91,2638.23] | 1202.36[497.88,2645.47] | 0.399 |
| log.sigma.3.0.mm.3D_glrlm_LongRunLowGrayLevelEmphasis (median [IQR]) | 0.22[0.09,0.49] | 0.19[0.08,0.49] | 0.24[0.14,0.47] | 0.409 |
| log.sigma.3.0.mm.3D_glrlm_LowGrayLevelRunEmphasis (median [IQR]) | 0.01[0.01,0.03] | 0.01[0.01,0.03] | 0.02[0.01,0.04] | 0.056 |
| log.sigma.3.0.mm.3D_glrlm_RunEntropy (median [IQR]) | 4.46[3.98,4.69] | 4.50[4.03,4.72] | 4.31[3.81,4.60] | 0.084 |
| log.sigma.3.0.mm.3D_glrlm_RunLengthNonUniformity (median [IQR]) | 3744.75[2042.48,7380.94] | 4442.54[2362.26,9094.07] | 2547.24[1132.96,4645.56] | 0.002 |
| log.sigma.3.0.mm.3D_glrlm_RunLengthNonUniformityNormalized (median [IQR]) | 0.36[0.30,0.43] | 0.36[0.31,0.43] | 0.36[0.29,0.43] | 0.72 |
| log.sigma.3.0.mm.3D_glrlm_RunPercentage (median [IQR]) | 0.47[0.42,0.54] | 0.47[0.43,0.54] | 0.47[0.42,0.53] | 0.8 |
| log.sigma.3.0.mm.3D_glrlm_RunVariance (median [IQR]) | 8.68[5.68,12.60] | 8.60[5.64,13.80] | 8.98[5.84,11.63] | 0.796 |
| log.sigma.3.0.mm.3D_glrlm_ShortRunEmphasis (median [IQR]) | 0.59[0.54,0.65] | 0.59[0.54,0.66] | 0.58[0.53,0.65] | 0.74 |
| log.sigma.3.0.mm.3D_glrlm_ShortRunHighGrayLevelEmphasis (median [IQR]) | 54.87[23.50,85.85] | 57.87[26.20,88.56] | 44.20[19.59,77.05] | 0.23 |
| log.sigma.3.0.mm.3D_glrlm_ShortRunLowGrayLevelEmphasis (median [IQR]) | 0.01[0.01,0.02] | 0.01[0.01,0.02] | 0.02[0.01,0.03] | 0.043 |
| log.sigma.3.0.mm.3D_glszm_GrayLevelNonUniformity (median [IQR]) | 33.02[19.17,52.60] | 34.71[23.01,56.99] | 22.49[9.24,42.48] | 0.004 |
| log.sigma.3.0.mm.3D_glszm_GrayLevelNonUniformityNormalized (median [IQR]) | 0.15[0.10,0.29] | 0.15[0.10,0.29] | 0.14[0.11,0.28] | 0.582 |
| log.sigma.3.0.mm.3D_glszm_GrayLevelVariance (median [IQR]) | 7.84[3.62,11.09] | 8.07[3.73,11.45] | 7.44[2.97,10.47] | 0.536 |
| log.sigma.3.0.mm.3D_glszm_HighGrayLevelZoneEmphasis (median [IQR]) | 95.98[45.58,136.86] | 98.59[59.11,140.27] | 81.76[42.40,110.61] | 0.118 |
| log.sigma.3.0.mm.3D_glszm_LargeAreaEmphasis (median [IQR]) | 779649.43[267652.27,1849846.07] | 911775.41[308638.42,2130887.36] | 570741.22[234022.35,1437783.92] | 0.079 |
| log.sigma.3.0.mm.3D_glszm_LargeAreaHighGrayLevelEmphasis (median [IQR]) | 59887672.88[12568705.14,205345844.20] | 70075097.04[16847526.10,251528478.24] | 36147798.89[7942636.33,113732813.22] | 0.05 |
| log.sigma.3.0.mm.3D_glszm_LargeAreaLowGrayLevelEmphasis (median [IQR]) | 10632.34[2469.46,39028.87] | 12930.72[2675.00,40048.90] | 5878.45[2301.44,29938.84] | 0.396 |
| log.sigma.3.0.mm.3D_glszm_LowGrayLevelZoneEmphasis (median [IQR]) | 0.02[0.02,0.05] | 0.02[0.02,0.04] | 0.04[0.02,0.08] | 0.059 |
| log.sigma.3.0.mm.3D_glszm_SizeZoneNonUniformity (median [IQR]) | 27.32[11.41,90.07] | 37.90[14.61,99.44] | 17.45[7.69,50.21] | 0.037 |
| log.sigma.3.0.mm.3D_glszm_SizeZoneNonUniformityNormalized (median [IQR]) | 0.17[0.13,0.21] | 0.17[0.13,0.21] | 0.16[0.13,0.21] | 0.878 |
| log.sigma.3.0.mm.3D_glszm_SmallAreaEmphasis (median [IQR]) | 0.42[0.35,0.47] | 0.42[0.35,0.47] | 0.41[0.35,0.46] | 0.747 |
| log.sigma.3.0.mm.3D_glszm_SmallAreaHighGrayLevelEmphasis (median [IQR]) | 41.58[18.51,65.77] | 46.46[20.98,66.15] | 24.37[17.59,65.18] | 0.154 |
| log.sigma.3.0.mm.3D_glszm_SmallAreaLowGrayLevelEmphasis (median [IQR]) | 0.01[0.01,0.01] | 0.01[0.00,0.01] | 0.01[0.01,0.01] | 0.162 |
| log.sigma.3.0.mm.3D_glszm_ZoneEntropy (median [IQR]) | 5.82[5.13,6.27] | 5.95[5.14,6.34] | 5.57[5.08,5.98] | 0.053 |
| log.sigma.3.0.mm.3D_glszm_ZonePercentage (median [IQR]) | 0.01[0.01,0.01] | 0.01[0.01,0.01] | 0.01[0.01,0.01] | 0.441 |
| log.sigma.3.0.mm.3D_glszm_ZoneVariance (median [IQR]) | 755167.99[262851.44,1821409.58] | 894869.57[296245.28,2097513.27] | 544641.02[206309.90,1389770.23] | 0.08 |
| log.sigma.3.0.mm.3D_ngtdm_Busyness (median [IQR]) | 10.17[4.72,19.05] | 11.09[5.32,19.22] | 6.21[3.61,18.03] | 0.073 |
| log.sigma.3.0.mm.3D_ngtdm_Coarseness (median [IQR]) | 0.00[0.00,0.00] | 0.00[0.00,0.00] | 0.00[0.00,0.00] | 0.003 |
| log.sigma.3.0.mm.3D_ngtdm_Complexity (median [IQR]) | 30.47[11.56,68.47] | 35.48[13.33,69.98] | 26.98[10.40,50.98] | 0.116 |
| log.sigma.3.0.mm.3D_ngtdm_Contrast (median [IQR]) | 0.01[0.00,0.01] | 0.01[0.00,0.01] | 0.01[0.00,0.01] | 0.973 |
| log.sigma.3.0.mm.3D_ngtdm_Strength (median [IQR]) | 0.09[0.04,0.18] | 0.07[0.04,0.14] | 0.13[0.04,0.26] | 0.05 |
| log.sigma.3.0.mm.3D_gldm_DependenceEntropy (median [IQR]) | 5.98[5.65,6.56] | 6.01[5.68,6.71] | 5.93[5.55,6.32] | 0.126 |
| log.sigma.3.0.mm.3D_gldm_DependenceNonUniformity (median [IQR]) | 1236.87[573.26,2308.39] | 1520.73[634.19,2521.04] | 704.94[315.91,1439.03] | 0.005 |
| log.sigma.3.0.mm.3D_gldm_DependenceNonUniformityNormalized (median [IQR]) | 0.05[0.05,0.06] | 0.05[0.05,0.06] | 0.05[0.05,0.06] | 0.821 |
| log.sigma.3.0.mm.3D_gldm_DependenceVariance (median [IQR]) | 45.83[41.02,50.57] | 45.25[41.29,49.57] | 46.97[40.24,51.59] | 0.991 |
| log.sigma.3.0.mm.3D_gldm_GrayLevelNonUniformity (median [IQR]) | 6801.56[3610.76,14266.93] | 8662.13[4128.55,15359.55] | 5314.53[2583.76,11109.86] | 0.045 |
| log.sigma.3.0.mm.3D_gldm_GrayLevelVariance (median [IQR]) | 1.67[0.62,3.98] | 1.99[0.62,4.30] | 1.39[0.63,3.03] | 0.575 |
| log.sigma.3.0.mm.3D_gldm_HighGrayLevelEmphasis (median [IQR]) | 99.14[42.26,142.50] | 112.22[50.79,154.83] | 85.27[36.50,137.67] | 0.26 |
| log.sigma.3.0.mm.3D_gldm_LargeDependenceEmphasis (median [IQR]) | 262.12[211.00,308.16] | 260.84[210.55,302.66] | 269.53[218.59,311.35] | 0.761 |
| log.sigma.3.0.mm.3D_gldm_LargeDependenceHighGrayLevelEmphasis (median [IQR]) | 25900.20[11168.31,41180.41] | 27064.23[12318.19,40769.60] | 21392.75[6020.82,43627.73] | 0.34 |
| log.sigma.3.0.mm.3D_gldm_LargeDependenceLowGrayLevelEmphasis (median [IQR]) | 2.69[1.44,6.69] | 2.55[1.42,6.21] | 3.15[2.22,7.56] | 0.25 |
| log.sigma.3.0.mm.3D_gldm_LowGrayLevelEmphasis (median [IQR]) | 0.01[0.01,0.03] | 0.01[0.01,0.02] | 0.02[0.01,0.03] | 0.085 |
| log.sigma.3.0.mm.3D_gldm_SmallDependenceEmphasis (median [IQR]) | 0.02[0.01,0.02] | 0.02[0.01,0.02] | 0.02[0.01,0.02] | 0.758 |
| log.sigma.3.0.mm.3D_gldm_SmallDependenceHighGrayLevelEmphasis (median [IQR]) | 1.42[0.66,3.17] | 1.53[0.68,3.24] | 1.24[0.66,2.59] | 0.367 |
| log.sigma.3.0.mm.3D_gldm_SmallDependenceLowGrayLevelEmphasis (median [IQR]) | 0.00[0.00,0.00] | 0.00[0.00,0.00] | 0.00[0.00,0.00] | 0.092 |
