# Supplementary material for: A Machine Learning Model for Predicting a Major Response to Neoadjuvant Chemotherapy in Advanced Gastric Cancer
Source: Front Oncol. 2021 Jun 1;11:675458. doi: 10.3389/fonc.2021.675458 (PMC8204104; doi:10.3389/fonc.2021.675458)
Supplement: Supplementary file 4 [file DataSheet_4.docx]

***Radiomic features of the minor and major response group in the validation cohort and results of univariate analysis***

|  | Overall  (n=77) | Minor response  (n=53) | Major response  (n=24) | p |
| --- | --- | --- | --- | --- |
| original_shape_Elongation (median [IQR]) | 0.73 [0.60, 0.84] | 0.74 [0.60, 0.85] | 0.73 [0.60, 0.81] | 0.834 |
| original_shape_Flatness (median [IQR]) | 0.47 [0.42, 0.56] | 0.47 [0.42, 0.57] | 0.47 [0.35, 0.54] | 0.391 |
| original_shape_LeastAxisLength (median [IQR]) | 30.00 [23.35, 45.05] | 31.08 [25.67, 48.76] | 23.58 [21.66, 35.22] | 0.004 |
| original_shape_MajorAxisLength (median [IQR]) | 63.41 [50.45, 91.22] | 76.68 [50.73, 94.30] | 59.00 [48.99, 71.75] | 0.194 |
| original_shape_Maximum2DDiameterColumn (median [IQR]) | 64.63 [52.74, 98.91] | 74.50 [53.91, 102.34] | 58.99 [51.50, 73.69] | 0.088 |
| original_shape_Maximum2DDiameterRow (median [IQR]) | 66.46 [50.73, 90.72] | 70.90 [52.12, 93.20] | 62.21 [47.70, 74.90] | 0.231 |
| original_shape_Maximum2DDiameterSlice (median [IQR]) | 72.33 [53.95, 105.37] | 84.11 [55.91, 107.78] | 64.09 [53.72, 83.66] | 0.144 |
| original_shape_Maximum3DDiameter (median [IQR]) | 79.15 [60.03, 114.90] | 95.54 [66.21, 120.55] | 65.09 [55.58, 79.79] | 0.014 |
| original_shape_MeshVolume (median [IQR]) | 31018.60 [18871.64, 73720.37] | 40324.78 [20178.31, 83989.13] | 26419.07 [16447.64, 40476.45] | 0.08 |
| original_shape_MinorAxisLength (median [IQR]) | 45.65 [38.35, 65.51] | 49.66 [39.52, 67.54] | 41.07 [35.04, 51.62] | 0.106 |
| original_shape_Sphericity (median [IQR]) | 0.40 [0.33, 0.44] | 0.40 [0.33, 0.44] | 0.40 [0.34, 0.47] | 0.708 |
| original_shape_SurfaceArea (median [IQR]) | 12068.25 [7422.10, 25589.24] | 14267.34 [7893.72, 28394.00] | 10159.30 [6110.17, 15181.27] | 0.116 |
| original_shape_SurfaceVolumeRatio (median [IQR]) | 0.37 [0.30, 0.44] | 0.36 [0.28, 0.43] | 0.39 [0.34, 0.48] | 0.071 |
| original_shape_VoxelVolume (median [IQR]) | 31123.51 [19025.66, 74559.84] | 40389.40 [20404.71, 84132.80] | 26511.97 [16566.88, 40683.78] | 0.077 |
| original_firstorder_10Percentile (median [IQR]) | 56.00 [44.00, 67.00] | 57.00 [45.00, 68.00] | 53.00 [43.25, 66.25] | 0.644 |
| original_firstorder_90Percentile (median [IQR]) | 108.00 [96.00, 123.00] | 109.00 [100.00, 123.00] | 103.00 [95.50, 124.50] | 0.541 |
| original_firstorder_Energy (median [IQR]) | 137870212.00 [70244820.00, 259667832.00] | 147245846.00 [78879651.00, 264797635.00] | 84519650.50 [49801227.75, 203917076.00] | 0.077 |
| original_firstorder_Entropy (median [IQR]) | 1.91 [1.72, 2.04] | 1.92 [1.73, 2.04] | 1.90 [1.71, 2.06] | 0.886 |
| original_firstorder_InterquartileRange (median [IQR]) | 28.00 [23.00, 30.00] | 28.00 [24.00, 30.00] | 27.00 [23.00, 32.00] | 0.895 |
| original_firstorder_Kurtosis (median [IQR]) | 4.38 [3.45, 5.30] | 4.26 [3.48, 5.30] | 4.49 [3.43, 5.14] | 0.895 |
| original_firstorder_Maximum (median [IQR]) | 164.00 [149.00, 187.00] | 165.00 [149.00, 187.00] | 160.50 [145.25, 187.50] | 0.644 |
| original_firstorder_MeanAbsoluteDeviation (median [IQR]) | 17.12 [14.55, 18.81] | 17.32 [14.55, 18.67] | 16.93 [14.60, 19.18] | 0.758 |
| original_firstorder_Mean (median [IQR]) | 81.32 [70.66, 95.51] | 84.77 [72.95, 94.75] | 78.94 [67.65, 97.15] | 0.613 |
| original_firstorder_Median (median [IQR]) | 83.00 [72.00, 97.00] | 86.00 [73.00, 95.00] | 79.50 [69.00, 99.25] | 0.516 |
| original_firstorder_Minimum (median [IQR]) | -54.00 [-134.00, -16.00] | -55.00 [-122.00, -15.00] | -46.00 [-135.00, -24.25] | 0.93 |
| original_firstorder_Range (median [IQR]) | 220.00 [179.00, 293.00] | 220.00 [180.00, 293.00] | 227.00 [170.50, 273.75] | 0.672 |
| original_firstorder_RobustMeanAbsoluteDeviation (median [IQR]) | 11.80 [9.91, 12.88] | 11.94 [10.15, 12.81] | 11.44 [9.76, 13.44] | 0.843 |
| original_firstorder_RootMeanSquared (median [IQR]) | 84.81 [73.84, 97.51] | 86.41 [76.91, 97.26] | 81.40 [71.18, 99.36] | 0.367 |
| original_firstorder_Skewness (median [IQR]) | -0.46 [-0.77, -0.17] | -0.48 [-0.79, -0.17] | -0.45 [-0.72, -0.17] | 0.775 |
| original_firstorder_TotalEnergy (median [IQR]) | 269168597.48 [142399052.87, 544340620.63] | 323498467.99 [191046086.65, 645675904.66] | 147662933.66 [103991890.01, 298835247.46] | 0.022 |
| original_firstorder_Uniformity (median [IQR]) | 0.32 [0.29, 0.36] | 0.32 [0.30, 0.36] | 0.32 [0.28, 0.37] | 0.939 |
| original_firstorder_Variance (median [IQR]) | 486.72 [367.36, 586.29] | 486.72 [373.04, 586.29] | 488.33 [357.48, 587.27] | 0.817 |
| original_glcm_Autocorrelation (median [IQR]) | 44.23 [30.81, 85.08] | 45.62 [33.74, 84.45] | 39.24 [29.54, 97.33] | 0.441 |
| original_glcm_ClusterProminence (median [IQR]) | 19.64 [11.40, 29.92] | 19.64 [12.70, 30.09] | 18.19 [11.07, 29.22] | 0.7 |
| original_glcm_ClusterShade (median [IQR]) | -0.77 [-1.40, -0.18] | -0.79 [-1.43, -0.26] | -0.59 [-1.17, -0.07] | 0.416 |
| original_glcm_ClusterTendency (median [IQR]) | 2.29 [1.71, 2.81] | 2.30 [1.73, 2.79] | 2.25 [1.63, 2.99] | 0.725 |
| original_glcm_Contrast (median [IQR]) | 0.81 [0.70, 1.00] | 0.81 [0.71, 0.97] | 0.80 [0.69, 1.09] | 0.965 |
| original_glcm_Correlation (median [IQR]) | 0.46 [0.39, 0.53] | 0.46 [0.39, 0.53] | 0.45 [0.34, 0.52] | 0.598 |
| original_glcm_DifferenceAverage (median [IQR]) | 0.59 [0.55, 0.69] | 0.60 [0.56, 0.68] | 0.58 [0.54, 0.71] | 0.93 |
| original_glcm_DifferenceEntropy (median [IQR]) | 1.32 [1.26, 1.43] | 1.32 [1.26, 1.42] | 1.31 [1.26, 1.47] | 0.991 |
| original_glcm_DifferenceVariance (median [IQR]) | 0.44 [0.39, 0.51] | 0.44 [0.39, 0.50] | 0.43 [0.39, 0.52] | 0.86 |
| original_glcm_Id (median [IQR]) | 0.73 [0.70, 0.75] | 0.73 [0.71, 0.75] | 0.74 [0.69, 0.75] | 0.965 |
| original_glcm_Idm (median [IQR]) | 0.72 [0.69, 0.74] | 0.72 [0.69, 0.74] | 0.73 [0.68, 0.74] | 0.956 |
| original_glcm_Idmn (median [IQR]) | 0.99 [0.99, 1.00] | 0.99 [0.99, 1.00] | 0.99 [0.99, 0.99] | 0.921 |
| original_glcm_Idn (median [IQR]) | 0.95 [0.94, 0.96] | 0.95 [0.94, 0.96] | 0.94 [0.94, 0.96] | 0.826 |
| original_glcm_Imc1 (median [IQR]) | -0.12 [-0.16, -0.09] | -0.13 [-0.16, -0.09] | -0.11 [-0.15, -0.07] | 0.262 |
| original_glcm_Imc2 (median [IQR]) | 0.55 [0.46, 0.61] | 0.55 [0.47, 0.61] | 0.51 [0.40, 0.59] | 0.281 |
| original_glcm_InverseVariance (median [IQR]) | 0.45 [0.43, 0.48] | 0.45 [0.43, 0.47] | 0.45 [0.43, 0.48] | 0.895 |
| original_glcm_JointAverage (median [IQR]) | 6.62 [5.54, 9.17] | 6.74 [5.78, 9.16] | 6.23 [5.39, 9.83] | 0.448 |
| original_glcm_JointEnergy (median [IQR]) | 0.14 [0.11, 0.16] | 0.13 [0.12, 0.16] | 0.14 [0.10, 0.17] | 0.956 |
| original_glcm_JointEntropy (median [IQR]) | 3.42 [3.14, 3.70] | 3.43 [3.15, 3.68] | 3.39 [3.11, 3.83] | 0.956 |
| original_glcm_MCC (median [IQR]) | 0.48 [0.41, 0.56] | 0.48 [0.41, 0.55] | 0.47 [0.35, 0.56] | 0.575 |
| original_glcm_MaximumProbability (median [IQR]) | 0.25 [0.21, 0.29] | 0.25 [0.22, 0.29] | 0.25 [0.19, 0.30] | 0.939 |
| original_glcm_SumAverage (median [IQR]) | 13.24 [11.07, 18.33] | 13.48 [11.55, 18.31] | 12.46 [10.78, 19.66] | 0.448 |
| original_glcm_SumEntropy (median [IQR]) | 2.59 [2.37, 2.75] | 2.59 [2.40, 2.70] | 2.58 [2.35, 2.81] | 0.817 |
| original_glcm_SumSquares (median [IQR]) | 0.78 [0.60, 0.94] | 0.79 [0.60, 0.93] | 0.77 [0.58, 0.96] | 0.775 |
| original_glrlm_GrayLevelNonUniformity (median [IQR]) | 3088.38 [1687.78, 5671.00] | 3595.74 [1716.13, 7819.82] | 2974.95 [1354.51, 4245.51] | 0.21 |
| original_glrlm_GrayLevelNonUniformityNormalized (median [IQR]) | 0.29 [0.27, 0.32] | 0.29 [0.27, 0.32] | 0.29 [0.26, 0.32] | 0.878 |
| original_glrlm_GrayLevelVariance (median [IQR]) | 1.04 [0.83, 1.19] | 1.04 [0.86, 1.18] | 1.06 [0.82, 1.20] | 0.852 |
| original_glrlm_HighGrayLevelRunEmphasis (median [IQR]) | 43.52 [30.11, 83.49] | 45.08 [32.60, 81.03] | 37.59 [28.66, 95.45] | 0.495 |
| original_glrlm_LongRunEmphasis (median [IQR]) | 4.72 [3.77, 5.77] | 4.61 [3.85, 5.66] | 4.83 [3.53, 5.84] | 0.725 |
| original_glrlm_LongRunHighGrayLevelEmphasis (median [IQR]) | 211.74 [139.34, 460.95] | 222.81 [140.21, 454.45] | 179.74 [130.56, 469.30] | 0.448 |
| original_glrlm_LongRunLowGrayLevelEmphasis (median [IQR]) | 0.11 [0.06, 0.16] | 0.11 [0.07, 0.15] | 0.14 [0.06, 0.17] | 0.741 |
| original_glrlm_LowGrayLevelRunEmphasis (median [IQR]) | 0.03 [0.01, 0.04] | 0.03 [0.01, 0.04] | 0.03 [0.01, 0.04] | 0.509 |
| original_glrlm_RunEntropy (median [IQR]) | 3.59 [3.36, 3.69] | 3.63 [3.33, 3.70] | 3.52 [3.38, 3.64] | 0.538 |
| original_glrlm_RunLengthNonUniformity (median [IQR]) | 5378.16 [2504.05, 9351.08] | 6207.85 [3132.32, 11177.38] | 4219.57 [2160.92, 7531.91] | 0.124 |
| original_glrlm_RunLengthNonUniformityNormalized (median [IQR]) | 0.47 [0.43, 0.52] | 0.47 [0.43, 0.50] | 0.47 [0.43, 0.53] | 0.684 |
| original_glrlm_RunPercentage (median [IQR]) | 0.62 [0.58, 0.66] | 0.62 [0.58, 0.65] | 0.61 [0.58, 0.67] | 0.741 |
| original_glrlm_RunVariance (median [IQR]) | 1.62 [1.16, 2.17] | 1.54 [1.21, 2.08] | 1.69 [1.05, 2.18] | 0.628 |
| original_glrlm_ShortRunEmphasis (median [IQR]) | 0.70 [0.67, 0.73] | 0.70 [0.67, 0.73] | 0.70 [0.67, 0.74] | 0.8 |
| original_glrlm_ShortRunHighGrayLevelEmphasis (median [IQR]) | 30.99 [21.71, 53.83] | 31.02 [22.99, 53.04] | 24.07 [19.42, 64.69] | 0.516 |
| original_glrlm_ShortRunLowGrayLevelEmphasis (median [IQR]) | 0.02 [0.01, 0.03] | 0.02 [0.01, 0.03] | 0.02 [0.01, 0.03] | 0.488 |
| original_glszm_GrayLevelNonUniformity (median [IQR]) | 98.34 [59.97, 215.84] | 116.08 [74.17, 242.99] | 83.57 [51.48, 164.32] | 0.111 |
| original_glszm_GrayLevelNonUniformityNormalized (median [IQR]) | 0.21 [0.18, 0.24] | 0.20 [0.19, 0.24] | 0.21 [0.18, 0.24] | 0.8 |
| original_glszm_GrayLevelVariance (median [IQR]) | 3.06 [2.54, 3.61] | 3.06 [2.52, 3.44] | 3.13 [2.58, 3.74] | 0.575 |
| original_glszm_HighGrayLevelZoneEmphasis (median [IQR]) | 35.37 [24.61, 75.13] | 37.96 [25.32, 73.20] | 30.81 [23.46, 81.71] | 0.628 |
| original_glszm_LargeAreaEmphasis (median [IQR]) | 168824.54 [81439.60, 403925.55] | 178769.53 [82856.97, 446456.43] | 151220.30 [77067.31, 296507.44] | 0.403 |
| original_glszm_LargeAreaHighGrayLevelEmphasis (median [IQR]) | 8998256.88 [2670145.53, 39798113.47] | 9463864.39 [3158093.24, 40945696.24] | 6993210.27 [2284144.04, 30914503.12] | 0.509 |
| original_glszm_LargeAreaLowGrayLevelEmphasis (median [IQR]) | 2976.29 [1720.84, 6385.47] | 3766.22 [1816.33, 8281.22] | 2421.95 [1700.85, 3960.85] | 0.163 |
| original_glszm_LowGrayLevelZoneEmphasis (median [IQR]) | 0.04 [0.02, 0.07] | 0.04 [0.02, 0.07] | 0.05 [0.02, 0.08] | 0.428 |
| original_glszm_SizeZoneNonUniformity (median [IQR]) | 122.89 [70.99, 297.59] | 148.13 [78.84, 390.43] | 98.33 [63.51, 205.93] | 0.092 |
| original_glszm_SizeZoneNonUniformityNormalized (median [IQR]) | 0.27 [0.24, 0.31] | 0.28 [0.24, 0.31] | 0.25 [0.24, 0.29] | 0.435 |
| original_glszm_SmallAreaEmphasis (median [IQR]) | 0.54 [0.49, 0.57] | 0.54 [0.50, 0.58] | 0.51 [0.49, 0.55] | 0.173 |
| original_glszm_SmallAreaHighGrayLevelEmphasis (median [IQR]) | 18.20 [12.21, 37.36] | 18.60 [12.35, 37.36] | 15.93 [11.80, 36.62] | 0.59 |
| original_glszm_SmallAreaLowGrayLevelEmphasis (median [IQR]) | 0.03 [0.01, 0.04] | 0.03 [0.01, 0.04] | 0.03 [0.01, 0.04] | 0.86 |
| original_glszm_ZoneEntropy (median [IQR]) | 5.12 [4.77, 5.42] | 5.11 [4.77, 5.39] | 5.14 [4.81, 5.42] | 0.895 |
| original_glszm_ZonePercentage (median [IQR]) | 0.03 [0.02, 0.04] | 0.03 [0.02, 0.04] | 0.03 [0.02, 0.04] | 0.725 |
| original_glszm_ZoneVariance (median [IQR]) | 158561.82 [76913.18, 359753.92] | 176991.02 [82336.29, 443992.32] | 93118.90 [38390.24, 229852.65] | 0.05 |
| original_ngtdm_Busyness (median [IQR]) | 20.18 [12.20, 32.01] | 22.92 [12.96, 42.00] | 17.23 [10.75, 22.78] | 0.044 |
| original_ngtdm_Coarseness (median [IQR]) | 0.00 [0.00, 0.00] | 0.00 [0.00, 0.00] | 0.00 [0.00, 0.00] | 0.113 |
| original_ngtdm_Complexity (median [IQR]) | 22.07 [14.71, 37.07] | 22.07 [14.71, 36.42] | 22.78 [14.64, 41.64] | 0.783 |
| original_ngtdm_Contrast (median [IQR]) | 0.01 [0.01, 0.01] | 0.01 [0.01, 0.01] | 0.01 [0.01, 0.01] | 0.912 |
| original_ngtdm_Strength (median [IQR]) | 0.03 [0.02, 0.06] | 0.03 [0.02, 0.05] | 0.03 [0.02, 0.06] | 0.333 |
| original_gldm_DependenceEntropy (median [IQR]) | 6.02 [5.83, 6.18] | 6.09 [5.85, 6.20] | 6.00 [5.78, 6.17] | 0.56 |
| original_gldm_DependenceNonUniformity (median [IQR]) | 980.75 [485.61, 1664.55] | 1149.79 [511.15, 2300.45] | 864.03 [390.23, 1422.67] | 0.271 |
| original_gldm_DependenceNonUniformityNormalized (median [IQR]) | 0.05 [0.05, 0.06] | 0.05 [0.05, 0.06] | 0.05 [0.05, 0.06] | 0.488 |
| original_gldm_DependenceVariance (median [IQR]) | 28.92 [22.60, 31.82] | 28.90 [24.21, 32.07] | 29.12 [21.24, 31.20] | 0.668 |
| original_gldm_GrayLevelNonUniformity (median [IQR]) | 5913.41 [2904.22, 11814.65] | 6351.68 [3059.42, 13641.28] | 5442.04 [2414.28, 9046.85] | 0.235 |
| original_gldm_GrayLevelVariance (median [IQR]) | 0.87 [0.67, 1.02] | 0.86 [0.68, 1.02] | 0.87 [0.66, 1.03] | 0.852 |
| original_gldm_HighGrayLevelEmphasis (median [IQR]) | 43.16 [30.24, 85.09] | 45.39 [32.95, 82.48] | 38.48 [29.41, 96.79] | 0.482 |
| original_gldm_LargeDependenceEmphasis (median [IQR]) | 150.61 [121.89, 177.04] | 149.49 [132.57, 177.04] | 154.94 [112.55, 177.00] | 0.792 |
| original_gldm_LargeDependenceHighGrayLevelEmphasis (median [IQR]) | 6714.93 [4272.34, 15070.02] | 7678.26 [4384.78, 15070.02] | 5824.95 [3897.12, 14228.52] | 0.385 |
| original_gldm_LargeDependenceLowGrayLevelEmphasis (median [IQR]) | 3.19 [1.74, 4.91] | 3.06 [2.06, 4.17] | 3.47 [1.67, 5.11] | 0.741 |
| original_gldm_LowGrayLevelEmphasis (median [IQR]) | 0.03 [0.01, 0.04] | 0.03 [0.01, 0.03] | 0.03 [0.01, 0.04] | 0.495 |
| original_gldm_SmallDependenceEmphasis (median [IQR]) | 0.04 [0.03, 0.05] | 0.04 [0.03, 0.04] | 0.04 [0.03, 0.05] | 0.86 |
| original_gldm_SmallDependenceHighGrayLevelEmphasis (median [IQR]) | 1.40 [1.00, 3.02] | 1.40 [1.05, 2.45] | 1.37 [0.92, 3.16] | 0.621 |
| original_gldm_SmallDependenceLowGrayLevelEmphasis (median [IQR]) | 0.00 [0.00, 0.00] | 0.00 [0.00, 0.00] | 0.00 [0.00, 0.00] | 0.502 |
| log.sigma.1.0.mm.3D_firstorder_10Percentile (median [IQR]) | -21.55 [-29.11, -18.10] | -21.55 [-28.48, -18.10] | -21.60 [-29.11, -18.34] | 0.939 |
| log.sigma.1.0.mm.3D_firstorder_90Percentile (median [IQR]) | 5.99 [4.30, 7.32] | 5.85 [4.30, 6.94] | 6.11 [4.74, 7.60] | 0.628 |
| log.sigma.1.0.mm.3D_firstorder_Energy (median [IQR]) | 5310872.53 [1821579.26, 23268721.77] | 5912170.73 [1872313.71, 27181770.51] | 4245050.83 [1461851.31, 17022605.12] | 0.21 |
| log.sigma.1.0.mm.3D_firstorder_Entropy (median [IQR]) | 1.34 [1.13, 1.55] | 1.34 [1.13, 1.50] | 1.30 [1.17, 1.57] | 0.878 |
| log.sigma.1.0.mm.3D_firstorder_InterquartileRange (median [IQR]) | 13.20 [11.74, 16.05] | 12.95 [11.74, 15.69] | 14.67 [11.74, 16.19] | 0.409 |
| log.sigma.1.0.mm.3D_firstorder_Kurtosis (median [IQR]) | 15.60 [4.94, 28.27] | 17.73 [5.15, 29.38] | 13.46 [3.40, 23.34] | 0.21 |
| log.sigma.1.0.mm.3D_firstorder_Maximum (median [IQR]) | 41.21 [30.27, 60.79] | 41.59 [28.94, 59.91] | 40.89 [32.18, 69.60] | 0.66 |
| log.sigma.1.0.mm.3D_firstorder_MeanAbsoluteDeviation (median [IQR]) | 10.21 [7.94, 13.87] | 10.46 [7.94, 13.87] | 9.85 [8.36, 13.44] | 0.741 |
| log.sigma.1.0.mm.3D_firstorder_Mean (median [IQR]) | -7.67 [-12.08, -5.72] | -7.63 [-12.39, -5.96] | -8.35 [-10.81, -5.32] | 0.965 |
| log.sigma.1.0.mm.3D_firstorder_Median (median [IQR]) | -5.21 [-6.89, -3.57] | -5.15 [-6.50, -3.50] | -5.70 [-7.16, -4.30] | 0.267 |
| log.sigma.1.0.mm.3D_firstorder_Minimum (median [IQR]) | -218.04 [-278.51, -73.32] | -233.29 [-278.51, -83.62] | -184.65 [-262.82, -56.33] | 0.385 |
| log.sigma.1.0.mm.3D_firstorder_Range (median [IQR]) | 264.57 [125.21, 316.17] | 281.24 [126.56, 316.17] | 243.31 [87.65, 310.84] | 0.379 |
| log.sigma.1.0.mm.3D_firstorder_RobustMeanAbsoluteDeviation (median [IQR]) | 5.82 [4.91, 6.80] | 5.62 [4.95, 6.80] | 6.13 [4.90, 6.86] | 0.575 |
| log.sigma.1.0.mm.3D_firstorder_RootMeanSquared (median [IQR]) | 17.83 [12.37, 27.45] | 18.60 [12.82, 28.77] | 16.90 [12.24, 25.60] | 0.717 |
| log.sigma.1.0.mm.3D_firstorder_Skewness (median [IQR]) | -2.93 [-3.93, -0.57] | -3.00 [-3.96, -0.67] | -2.55 [-3.44, -0.33] | 0.276 |
| log.sigma.1.0.mm.3D_firstorder_TotalEnergy (median [IQR]) | 12478824.15 [3206617.21, 43597470.46] | 14697400.03 [4393194.20, 52669481.40] | 7065326.81 [3101770.25, 28525917.34] | 0.169 |
| log.sigma.1.0.mm.3D_firstorder_Uniformity (median [IQR]) | 0.50 [0.44, 0.53] | 0.49 [0.44, 0.53] | 0.50 [0.44, 0.52] | 0.895 |
| log.sigma.1.0.mm.3D_firstorder_Variance (median [IQR]) | 279.94 [119.33, 587.58] | 281.88 [119.33, 664.23] | 236.15 [118.61, 509.89] | 0.652 |
| log.sigma.1.0.mm.3D_glcm_Autocorrelation (median [IQR]) | 85.28 [11.60, 145.48] | 101.48 [17.76, 145.48] | 68.96 [11.03, 130.37] | 0.356 |
| log.sigma.1.0.mm.3D_glcm_ClusterProminence (median [IQR]) | 30.01 [1.89, 172.71] | 37.10 [2.21, 210.25] | 20.30 [1.59, 107.11] | 0.403 |
| log.sigma.1.0.mm.3D_glcm_ClusterShade (median [IQR]) | -3.49 [-14.89, -0.05] | -3.49 [-19.40, -0.06] | -2.40 [-10.12, -0.04] | 0.516 |
| log.sigma.1.0.mm.3D_glcm_ClusterTendency (median [IQR]) | 1.30 [0.73, 2.49] | 1.37 [0.74, 2.85] | 1.23 [0.72, 2.28] | 0.545 |
| log.sigma.1.0.mm.3D_glcm_Contrast (median [IQR]) | 0.60 [0.42, 0.85] | 0.61 [0.42, 0.78] | 0.56 [0.43, 0.86] | 0.809 |
| log.sigma.1.0.mm.3D_glcm_Correlation (median [IQR]) | 0.36 [0.28, 0.50] | 0.36 [0.30, 0.52] | 0.38 [0.27, 0.48] | 0.733 |
| log.sigma.1.0.mm.3D_glcm_DifferenceAverage (median [IQR]) | 0.43 [0.39, 0.51] | 0.43 [0.39, 0.51] | 0.44 [0.38, 0.53] | 0.947 |
| log.sigma.1.0.mm.3D_glcm_DifferenceEntropy (median [IQR]) | 1.14 [1.01, 1.27] | 1.14 [1.01, 1.26] | 1.14 [1.02, 1.27] | 0.982 |
| log.sigma.1.0.mm.3D_glcm_DifferenceVariance (median [IQR]) | 0.37 [0.26, 0.54] | 0.38 [0.26, 0.52] | 0.36 [0.26, 0.54] | 0.783 |
| log.sigma.1.0.mm.3D_glcm_Id (median [IQR]) | 0.80 [0.77, 0.82] | 0.80 [0.78, 0.82] | 0.79 [0.77, 0.81] | 0.8 |
| log.sigma.1.0.mm.3D_glcm_Idm (median [IQR]) | 0.79 [0.77, 0.81] | 0.79 [0.77, 0.81] | 0.79 [0.76, 0.81] | 0.843 |
| log.sigma.1.0.mm.3D_glcm_Idmn (median [IQR]) | 0.99 [0.99, 1.00] | 0.99 [0.99, 1.00] | 0.99 [0.99, 1.00] | 0.775 |
| log.sigma.1.0.mm.3D_glcm_Idn (median [IQR]) | 0.96 [0.95, 0.97] | 0.96 [0.94, 0.97] | 0.96 [0.95, 0.97] | 0.621 |
| log.sigma.1.0.mm.3D_glcm_Imc1 (median [IQR]) | -0.10 [-0.13, -0.08] | -0.11 [-0.14, -0.08] | -0.10 [-0.13, -0.08] | 0.455 |
| log.sigma.1.0.mm.3D_glcm_Imc2 (median [IQR]) | 0.41 [0.33, 0.50] | 0.41 [0.35, 0.51] | 0.41 [0.31, 0.48] | 0.344 |
| log.sigma.1.0.mm.3D_glcm_InverseVariance (median [IQR]) | 0.37 [0.33, 0.39] | 0.37 [0.33, 0.39] | 0.38 [0.34, 0.40] | 0.575 |
| log.sigma.1.0.mm.3D_glcm_JointAverage (median [IQR]) | 9.22 [3.39, 12.04] | 10.04 [4.21, 12.04] | 8.29 [3.31, 11.40] | 0.361 |
| log.sigma.1.0.mm.3D_glcm_JointEnergy (median [IQR]) | 0.28 [0.23, 0.32] | 0.28 [0.23, 0.32] | 0.28 [0.24, 0.31] | 0.8 |
| log.sigma.1.0.mm.3D_glcm_JointEntropy (median [IQR]) | 2.37 [2.15, 2.69] | 2.36 [2.14, 2.69] | 2.40 [2.15, 2.72] | 1 |
| log.sigma.1.0.mm.3D_glcm_MCC (median [IQR]) | 0.50 [0.35, 0.58] | 0.50 [0.38, 0.58] | 0.47 [0.31, 0.56] | 0.409 |
| log.sigma.1.0.mm.3D_glcm_MaximumProbability (median [IQR]) | 0.44 [0.38, 0.51] | 0.44 [0.37, 0.51] | 0.43 [0.38, 0.49] | 0.86 |
| log.sigma.1.0.mm.3D_glcm_SumAverage (median [IQR]) | 18.44 [6.78, 24.07] | 20.08 [8.41, 24.07] | 16.59 [6.62, 22.80] | 0.361 |
| log.sigma.1.0.mm.3D_glcm_SumEntropy (median [IQR]) | 1.88 [1.69, 2.09] | 1.88 [1.69, 2.07] | 1.87 [1.72, 2.11] | 0.878 |
| log.sigma.1.0.mm.3D_glcm_SumSquares (median [IQR]) | 0.48 [0.29, 0.84] | 0.49 [0.29, 0.89] | 0.46 [0.29, 0.75] | 0.59 |
| log.sigma.1.0.mm.3D_glrlm_GrayLevelNonUniformity (median [IQR]) | 3851.80 [1912.04, 6933.70] | 4157.15 [2284.68, 8402.37] | 3476.02 [1478.28, 4955.09] | 0.173 |
| log.sigma.1.0.mm.3D_glrlm_GrayLevelNonUniformityNormalized (median [IQR]) | 0.41 [0.36, 0.46] | 0.41 [0.36, 0.47] | 0.42 [0.35, 0.45] | 0.852 |
| log.sigma.1.0.mm.3D_glrlm_GrayLevelVariance (median [IQR]) | 0.82 [0.38, 1.78] | 0.87 [0.38, 1.98] | 0.67 [0.37, 1.44] | 0.455 |
| log.sigma.1.0.mm.3D_glrlm_HighGrayLevelRunEmphasis (median [IQR]) | 82.47 [11.81, 140.79] | 98.76 [17.98, 140.79] | 68.63 [11.12, 127.33] | 0.339 |
| log.sigma.1.0.mm.3D_glrlm_LongRunEmphasis (median [IQR]) | 8.07 [6.96, 9.53] | 8.25 [7.12, 9.47] | 7.93 [6.76, 9.86] | 0.725 |
| log.sigma.1.0.mm.3D_glrlm_LongRunHighGrayLevelEmphasis (median [IQR]) | 642.68 [122.73, 1129.30] | 783.85 [133.26, 1176.34] | 553.22 [103.70, 941.01] | 0.344 |
| log.sigma.1.0.mm.3D_glrlm_LongRunLowGrayLevelEmphasis (median [IQR]) | 0.10 [0.05, 0.61] | 0.09 [0.05, 0.54] | 0.14 [0.05, 0.67] | 0.455 |
| log.sigma.1.0.mm.3D_glrlm_LowGrayLevelRunEmphasis (median [IQR]) | 0.01 [0.01, 0.09] | 0.01 [0.01, 0.06] | 0.02 [0.01, 0.11] | 0.385 |
| log.sigma.1.0.mm.3D_glrlm_RunEntropy (median [IQR]) | 3.43 [3.23, 3.70] | 3.43 [3.24, 3.70] | 3.41 [3.22, 3.70] | 0.598 |
| log.sigma.1.0.mm.3D_glrlm_RunLengthNonUniformity (median [IQR]) | 3722.78 [1740.44, 7615.79] | 4598.78 [1815.36, 8969.67] | 3148.50 [1485.98, 5458.27] | 0.191 |
| log.sigma.1.0.mm.3D_glrlm_RunLengthNonUniformityNormalized (median [IQR]) | 0.37 [0.34, 0.41] | 0.36 [0.34, 0.40] | 0.39 [0.36, 0.41] | 0.296 |
| log.sigma.1.0.mm.3D_glrlm_RunPercentage (median [IQR]) | 0.52 [0.49, 0.55] | 0.51 [0.49, 0.54] | 0.54 [0.49, 0.56] | 0.322 |
| log.sigma.1.0.mm.3D_glrlm_RunVariance (median [IQR]) | 3.09 [2.71, 3.62] | 3.09 [2.72, 3.62] | 3.11 [2.59, 3.64] | 0.717 |
| log.sigma.1.0.mm.3D_glrlm_ShortRunEmphasis (median [IQR]) | 0.60 [0.57, 0.64] | 0.59 [0.57, 0.63] | 0.61 [0.59, 0.65] | 0.361 |
| log.sigma.1.0.mm.3D_glrlm_ShortRunHighGrayLevelEmphasis (median [IQR]) | 50.36 [7.44, 83.57] | 59.34 [10.27, 86.64] | 39.90 [6.65, 82.15] | 0.455 |
| log.sigma.1.0.mm.3D_glrlm_ShortRunLowGrayLevelEmphasis (median [IQR]) | 0.01 [0.01, 0.05] | 0.01 [0.01, 0.04] | 0.01 [0.01, 0.07] | 0.328 |
| log.sigma.1.0.mm.3D_glszm_GrayLevelNonUniformity (median [IQR]) | 61.87 [35.81, 134.80] | 69.74 [38.10, 156.68] | 52.29 [35.57, 77.77] | 0.095 |
| log.sigma.1.0.mm.3D_glszm_GrayLevelNonUniformityNormalized (median [IQR]) | 0.20 [0.13, 0.39] | 0.19 [0.13, 0.39] | 0.21 [0.16, 0.40] | 0.516 |
| log.sigma.1.0.mm.3D_glszm_GrayLevelVariance (median [IQR]) | 4.72 [1.39, 6.63] | 5.03 [1.42, 6.82] | 4.17 [1.37, 6.01] | 0.575 |
| log.sigma.1.0.mm.3D_glszm_HighGrayLevelZoneEmphasis (median [IQR]) | 57.72 [13.18, 95.74] | 63.04 [14.39, 88.94] | 54.28 [9.33, 95.84] | 0.448 |
| log.sigma.1.0.mm.3D_glszm_LargeAreaEmphasis (median [IQR]) | 537235.63 [239647.15, 1003323.84] | 583658.51 [295376.47, 1222381.03] | 411796.77 [220357.30, 919835.38] | 0.276 |
| log.sigma.1.0.mm.3D_glszm_LargeAreaHighGrayLevelEmphasis (median [IQR]) | 28628951.07 [6552989.74, 116828072.67] | 36181597.76 [6713280.53, 116828072.67] | 22621112.42 [5560238.62, 82056152.74] | 0.448 |
| log.sigma.1.0.mm.3D_glszm_LargeAreaLowGrayLevelEmphasis (median [IQR]) | 8505.18 [3490.19, 29844.40] | 8505.18 [2924.77, 43996.03] | 9622.17 [3784.80, 24267.19] | 0.886 |
| log.sigma.1.0.mm.3D_glszm_LowGrayLevelZoneEmphasis (median [IQR]) | 0.03 [0.02, 0.13] | 0.03 [0.02, 0.13] | 0.04 [0.02, 0.19] | 0.524 |
| log.sigma.1.0.mm.3D_glszm_SizeZoneNonUniformity (median [IQR]) | 49.71 [22.84, 152.51] | 50.36 [29.68, 177.21] | 49.30 [22.75, 88.94] | 0.235 |
| log.sigma.1.0.mm.3D_glszm_SizeZoneNonUniformityNormalized (median [IQR]) | 0.21 [0.18, 0.24] | 0.21 [0.17, 0.24] | 0.21 [0.18, 0.23] | 0.809 |
| log.sigma.1.0.mm.3D_glszm_SmallAreaEmphasis (median [IQR]) | 0.45 [0.40, 0.49] | 0.45 [0.40, 0.50] | 0.45 [0.41, 0.47] | 0.733 |
| log.sigma.1.0.mm.3D_glszm_SmallAreaHighGrayLevelEmphasis (median [IQR]) | 26.54 [6.01, 39.01] | 27.28 [6.01, 38.04] | 24.80 [5.83, 40.67] | 0.582 |
| log.sigma.1.0.mm.3D_glszm_SmallAreaLowGrayLevelEmphasis (median [IQR]) | 0.02 [0.01, 0.04] | 0.02 [0.01, 0.03] | 0.02 [0.01, 0.05] | 0.991 |
| log.sigma.1.0.mm.3D_glszm_ZoneEntropy (median [IQR]) | 5.39 [4.39, 5.94] | 5.39 [4.59, 5.94] | 5.19 [4.31, 6.00] | 0.628 |
| log.sigma.1.0.mm.3D_glszm_ZonePercentage (median [IQR]) | 0.02 [0.01, 0.02] | 0.02 [0.01, 0.02] | 0.02 [0.01, 0.02] | 0.783 |
| log.sigma.1.0.mm.3D_glszm_ZoneVariance (median [IQR]) | 530394.95 [229935.33, 974174.64] | 582964.66 [294854.23, 1208994.29] | 381169.32 [189212.28, 905732.66] | 0.095 |
| log.sigma.1.0.mm.3D_ngtdm_Busyness (median [IQR]) | 20.95 [11.32, 57.43] | 22.38 [11.49, 62.08] | 18.67 [10.90, 36.05] | 0.416 |
| log.sigma.1.0.mm.3D_ngtdm_Coarseness (median [IQR]) | 0.00 [0.00, 0.00] | 0.00 [0.00, 0.00] | 0.00 [0.00, 0.00] | 0.106 |
| log.sigma.1.0.mm.3D_ngtdm_Complexity (median [IQR]) | 28.97 [5.94, 49.41] | 36.52 [6.38, 52.90] | 24.94 [4.35, 48.02] | 0.356 |
| log.sigma.1.0.mm.3D_ngtdm_Contrast (median [IQR]) | 0.01 [0.00, 0.01] | 0.01 [0.00, 0.01] | 0.00 [0.00, 0.01] | 0.991 |
| log.sigma.1.0.mm.3D_ngtdm_Strength (median [IQR]) | 0.05 [0.01, 0.10] | 0.04 [0.01, 0.10] | 0.05 [0.01, 0.09] | 0.956 |
| log.sigma.1.0.mm.3D_gldm_DependenceEntropy (median [IQR]) | 5.51 [5.36, 5.72] | 5.53 [5.36, 5.72] | 5.51 [5.38, 5.73] | 0.93 |
| log.sigma.1.0.mm.3D_gldm_DependenceNonUniformity (median [IQR]) | 933.64 [451.18, 1595.61] | 1001.43 [456.05, 2022.99] | 820.46 [355.04, 1233.33] | 0.244 |
| log.sigma.1.0.mm.3D_gldm_DependenceNonUniformityNormalized (median [IQR]) | 0.05 [0.05, 0.05] | 0.05 [0.05, 0.05] | 0.05 [0.05, 0.05] | 0.965 |
| log.sigma.1.0.mm.3D_gldm_DependenceVariance (median [IQR]) | 32.47 [29.48, 37.80] | 33.03 [29.50, 37.45] | 31.61 [28.74, 38.68] | 0.878 |
| log.sigma.1.0.mm.3D_gldm_GrayLevelNonUniformity (median [IQR]) | 8659.20 [4566.42, 17992.13] | 9350.29 [5438.21, 19328.73] | 7569.15 [3117.19, 12015.28] | 0.202 |
| log.sigma.1.0.mm.3D_gldm_GrayLevelVariance (median [IQR]) | 0.55 [0.30, 1.08] | 0.56 [0.29, 1.13] | 0.48 [0.30, 0.91] | 0.553 |
| log.sigma.1.0.mm.3D_gldm_HighGrayLevelEmphasis (median [IQR]) | 84.38 [11.72, 144.42] | 100.80 [17.71, 144.42] | 68.76 [11.00, 128.98] | 0.356 |
| log.sigma.1.0.mm.3D_gldm_LargeDependenceEmphasis (median [IQR]) | 214.97 [194.54, 237.35] | 215.95 [198.52, 237.35] | 202.82 [184.95, 235.76] | 0.317 |
| log.sigma.1.0.mm.3D_gldm_LargeDependenceHighGrayLevelEmphasis (median [IQR]) | 17121.26 [2757.80, 28726.47] | 20606.38 [2935.40, 29910.68] | 14564.63 [2207.21, 25167.45] | 0.262 |
| log.sigma.1.0.mm.3D_gldm_LargeDependenceLowGrayLevelEmphasis (median [IQR]) | 2.44 [1.29, 15.93] | 2.18 [1.31, 15.02] | 3.35 [1.26, 20.50] | 0.509 |
| log.sigma.1.0.mm.3D_gldm_LowGrayLevelEmphasis (median [IQR]) | 0.01 [0.01, 0.09] | 0.01 [0.01, 0.06] | 0.02 [0.01, 0.10] | 0.339 |
| log.sigma.1.0.mm.3D_gldm_SmallDependenceEmphasis (median [IQR]) | 0.02 [0.02, 0.03] | 0.02 [0.02, 0.03] | 0.02 [0.02, 0.03] | 0.684 |
| log.sigma.1.0.mm.3D_gldm_SmallDependenceHighGrayLevelEmphasis (median [IQR]) | 1.59 [0.28, 3.17] | 1.70 [0.28, 3.03] | 1.24 [0.21, 3.47] | 0.582 |
| log.sigma.1.0.mm.3D_gldm_SmallDependenceLowGrayLevelEmphasis (median [IQR]) | 0.00 [0.00, 0.00] | 0.00 [0.00, 0.00] | 0.00 [0.00, 0.00] | 0.495 |
| log.sigma.1.5.mm.3D_firstorder_10Percentile (median [IQR]) | -32.10 [-46.92, -26.07] | -32.10 [-55.30, -26.15] | -31.37 [-44.64, -25.91] | 0.843 |
| log.sigma.1.5.mm.3D_firstorder_90Percentile (median [IQR]) | 3.41 [1.60, 5.23] | 3.41 [1.73, 5.16] | 3.40 [0.34, 5.33] | 0.733 |
| log.sigma.1.5.mm.3D_firstorder_Energy (median [IQR]) | 13116260.69 [2911648.79, 51123153.90] | 16029785.78 [4025846.12, 68865599.40] | 9347774.96 [2596662.35, 43591600.78] | 0.218 |
| log.sigma.1.5.mm.3D_firstorder_Entropy (median [IQR]) | 1.55 [1.21, 1.88] | 1.57 [1.22, 1.86] | 1.52 [1.21, 1.88] | 0.8 |
| log.sigma.1.5.mm.3D_firstorder_InterquartileRange (median [IQR]) | 17.22 [14.50, 20.85] | 16.94 [14.54, 20.67] | 17.67 [13.36, 21.26] | 0.895 |
| log.sigma.1.5.mm.3D_firstorder_Kurtosis (median [IQR]) | 11.43 [3.99, 22.44] | 11.43 [4.06, 25.21] | 10.60 [3.46, 19.18] | 0.291 |
| log.sigma.1.5.mm.3D_firstorder_Maximum (median [IQR]) | 50.67 [30.74, 76.80] | 50.67 [30.21, 76.65] | 51.86 [33.66, 81.23] | 0.455 |
| log.sigma.1.5.mm.3D_firstorder_MeanAbsoluteDeviation (median [IQR]) | 14.43 [9.62, 20.51] | 14.80 [9.62, 24.51] | 12.68 [9.57, 19.75] | 0.676 |
| log.sigma.1.5.mm.3D_firstorder_Mean (median [IQR]) | -14.53 [-22.87, -10.65] | -14.36 [-24.34, -10.79] | -14.75 [-20.54, -10.11] | 0.86 |
| log.sigma.1.5.mm.3D_firstorder_Median (median [IQR]) | -9.59 [-12.93, -6.43] | -9.32 [-12.93, -6.32] | -10.93 [-13.36, -7.71] | 0.385 |
| log.sigma.1.5.mm.3D_firstorder_Minimum (median [IQR]) | -273.89 [-344.02, -77.10] | -287.06 [-344.37, -79.78] | -229.50 [-333.59, -68.59] | 0.367 |
| log.sigma.1.5.mm.3D_firstorder_Range (median [IQR]) | 311.09 [121.00, 398.96] | 315.73 [126.73, 398.96] | 284.85 [107.34, 398.04] | 0.509 |
| log.sigma.1.5.mm.3D_firstorder_RobustMeanAbsoluteDeviation (median [IQR]) | 7.35 [6.11, 9.44] | 7.35 [6.13, 9.46] | 7.35 [5.94, 9.19] | 0.878 |
| log.sigma.1.5.mm.3D_firstorder_RootMeanSquared (median [IQR]) | 29.64 [17.07, 41.07] | 31.18 [17.07, 47.73] | 23.54 [17.32, 39.37] | 0.652 |
| log.sigma.1.5.mm.3D_firstorder_Skewness (median [IQR]) | -2.48 [-3.57, -0.72] | -2.51 [-3.68, -0.72] | -2.18 [-3.33, -0.42] | 0.244 |
| log.sigma.1.5.mm.3D_firstorder_TotalEnergy (median [IQR]) | 31971890.10 [6503612.04, 90470612.94] | 36460675.06 [8642260.00, 113706261.73] | 16650764.28 [5089890.32, 66981459.80] | 0.15 |
| log.sigma.1.5.mm.3D_firstorder_Uniformity (median [IQR]) | 0.46 [0.38, 0.53] | 0.46 [0.39, 0.53] | 0.47 [0.38, 0.52] | 0.895 |
| log.sigma.1.5.mm.3D_firstorder_Variance (median [IQR]) | 653.39 [164.53, 1317.49] | 736.35 [173.20, 1679.11] | 401.99 [147.11, 1211.62] | 0.441 |
| log.sigma.1.5.mm.3D_glcm_Autocorrelation (median [IQR]) | 115.39 [16.89, 190.82] | 121.84 [16.95, 190.82] | 93.68 [10.44, 186.07] | 0.403 |
| log.sigma.1.5.mm.3D_glcm_ClusterProminence (median [IQR]) | 219.80 [3.90, 809.83] | 275.26 [4.42, 1096.26] | 76.99 [3.05, 467.43] | 0.291 |
| log.sigma.1.5.mm.3D_glcm_ClusterShade (median [IQR]) | -18.18 [-59.71, -0.27] | -18.77 [-70.09, -0.40] | -7.67 [-33.85, -0.17] | 0.361 |
| log.sigma.1.5.mm.3D_glcm_ClusterTendency (median [IQR]) | 2.69 [0.93, 6.04] | 3.20 [0.94, 8.22] | 2.09 [0.84, 4.91] | 0.409 |
| log.sigma.1.5.mm.3D_glcm_Contrast (median [IQR]) | 0.71 [0.40, 1.19] | 0.73 [0.40, 1.24] | 0.64 [0.40, 1.18] | 0.7 |
| log.sigma.1.5.mm.3D_glcm_Correlation (median [IQR]) | 0.57 [0.43, 0.68] | 0.56 [0.44, 0.69] | 0.58 [0.43, 0.68] | 0.575 |
| log.sigma.1.5.mm.3D_glcm_DifferenceAverage (median [IQR]) | 0.43 [0.35, 0.57] | 0.42 [0.35, 0.57] | 0.44 [0.36, 0.58] | 0.939 |
| log.sigma.1.5.mm.3D_glcm_DifferenceEntropy (median [IQR]) | 1.17 [0.97, 1.36] | 1.17 [0.97, 1.33] | 1.19 [0.98, 1.37] | 0.843 |
| log.sigma.1.5.mm.3D_glcm_DifferenceVariance (median [IQR]) | 0.46 [0.25, 0.80] | 0.48 [0.25, 0.87] | 0.41 [0.25, 0.77] | 0.652 |
| log.sigma.1.5.mm.3D_glcm_Id (median [IQR]) | 0.81 [0.78, 0.84] | 0.81 [0.78, 0.84] | 0.81 [0.77, 0.83] | 0.921 |
| log.sigma.1.5.mm.3D_glcm_Idm (median [IQR]) | 0.80 [0.77, 0.83] | 0.80 [0.77, 0.83] | 0.80 [0.76, 0.83] | 0.982 |
| log.sigma.1.5.mm.3D_glcm_Idmn (median [IQR]) | 1.00 [0.99, 1.00] | 1.00 [0.99, 1.00] | 1.00 [0.99, 1.00] | 0.792 |
| log.sigma.1.5.mm.3D_glcm_Idn (median [IQR]) | 0.97 [0.95, 0.97] | 0.97 [0.95, 0.98] | 0.97 [0.96, 0.97] | 0.733 |
| log.sigma.1.5.mm.3D_glcm_Imc1 (median [IQR]) | -0.19 [-0.23, -0.16] | -0.19 [-0.24, -0.16] | -0.18 [-0.22, -0.15] | 0.253 |
| log.sigma.1.5.mm.3D_glcm_Imc2 (median [IQR]) | 0.59 [0.50, 0.69] | 0.59 [0.51, 0.70] | 0.57 [0.49, 0.65] | 0.239 |
| log.sigma.1.5.mm.3D_glcm_InverseVariance (median [IQR]) | 0.32 [0.29, 0.35] | 0.32 [0.29, 0.35] | 0.34 [0.30, 0.35] | 0.448 |
| log.sigma.1.5.mm.3D_glcm_JointAverage (median [IQR]) | 10.70 [4.10, 13.78] | 11.03 [4.10, 13.78] | 9.65 [3.21, 13.61] | 0.422 |
| log.sigma.1.5.mm.3D_glcm_JointEnergy (median [IQR]) | 0.27 [0.22, 0.36] | 0.28 [0.22, 0.36] | 0.27 [0.22, 0.34] | 1 |
| log.sigma.1.5.mm.3D_glcm_JointEntropy (median [IQR]) | 2.60 [2.18, 3.17] | 2.60 [2.18, 3.17] | 2.66 [2.20, 3.15] | 0.895 |
| log.sigma.1.5.mm.3D_glcm_MCC (median [IQR]) | 0.65 [0.48, 0.75] | 0.66 [0.51, 0.75] | 0.62 [0.47, 0.72] | 0.495 |
| log.sigma.1.5.mm.3D_glcm_MaximumProbability (median [IQR]) | 0.48 [0.39, 0.56] | 0.49 [0.39, 0.57] | 0.46 [0.41, 0.54] | 0.75 |
| log.sigma.1.5.mm.3D_glcm_SumAverage (median [IQR]) | 21.40 [8.20, 27.57] | 22.05 [8.20, 27.57] | 19.30 [6.43, 27.22] | 0.422 |
| log.sigma.1.5.mm.3D_glcm_SumEntropy (median [IQR]) | 2.12 [1.78, 2.48] | 2.12 [1.79, 2.48] | 2.14 [1.77, 2.46] | 0.783 |
| log.sigma.1.5.mm.3D_glcm_SumSquares (median [IQR]) | 0.86 [0.32, 1.71] | 0.99 [0.35, 2.28] | 0.68 [0.31, 1.53] | 0.461 |
| log.sigma.1.5.mm.3D_glrlm_GrayLevelNonUniformity (median [IQR]) | 3093.77 [1565.91, 5486.33] | 3272.18 [1939.20, 6629.50] | 2723.50 [1251.19, 4234.89] | 0.218 |
| log.sigma.1.5.mm.3D_glrlm_GrayLevelNonUniformityNormalized (median [IQR]) | 0.34 [0.28, 0.42] | 0.33 [0.27, 0.42] | 0.35 [0.29, 0.41] | 0.775 |
| log.sigma.1.5.mm.3D_glrlm_GrayLevelVariance (median [IQR]) | 2.02 [0.48, 4.16] | 2.06 [0.48, 4.77] | 1.18 [0.47, 3.08] | 0.312 |
| log.sigma.1.5.mm.3D_glrlm_HighGrayLevelRunEmphasis (median [IQR]) | 111.10 [16.44, 180.66] | 120.46 [17.51, 180.66] | 91.60 [10.91, 180.57] | 0.403 |
| log.sigma.1.5.mm.3D_glrlm_LongRunEmphasis (median [IQR]) | 12.30 [9.88, 15.54] | 12.46 [9.95, 15.68] | 11.23 [9.85, 15.01] | 0.605 |
| log.sigma.1.5.mm.3D_glrlm_LongRunHighGrayLevelEmphasis (median [IQR]) | 1387.34 [210.30, 2200.37] | 1470.36 [228.17, 2464.04] | 1039.58 [169.07, 1797.46] | 0.296 |
| log.sigma.1.5.mm.3D_glrlm_LongRunLowGrayLevelEmphasis (median [IQR]) | 0.11 [0.06, 0.84] | 0.10 [0.06, 0.79] | 0.13 [0.06, 1.23] | 0.605 |
| log.sigma.1.5.mm.3D_glrlm_LowGrayLevelRunEmphasis (median [IQR]) | 0.01 [0.01, 0.07] | 0.01 [0.01, 0.06] | 0.01 [0.01, 0.11] | 0.553 |
| log.sigma.1.5.mm.3D_glrlm_RunEntropy (median [IQR]) | 3.85 [3.60, 4.22] | 3.90 [3.64, 4.22] | 3.71 [3.59, 4.08] | 0.488 |
| log.sigma.1.5.mm.3D_glrlm_RunLengthNonUniformity (median [IQR]) | 3214.20 [1440.66, 7727.74] | 3802.14 [1440.66, 8854.50] | 2872.57 [1420.79, 5718.70] | 0.226 |
| log.sigma.1.5.mm.3D_glrlm_RunLengthNonUniformityNormalized (median [IQR]) | 0.36 [0.32, 0.41] | 0.36 [0.31, 0.41] | 0.39 [0.33, 0.42] | 0.495 |
| log.sigma.1.5.mm.3D_glrlm_RunPercentage (median [IQR]) | 0.50 [0.45, 0.54] | 0.49 [0.44, 0.53] | 0.51 [0.46, 0.54] | 0.403 |
| log.sigma.1.5.mm.3D_glrlm_RunVariance (median [IQR]) | 5.78 [4.60, 7.08] | 5.78 [4.64, 7.08] | 5.68 [3.78, 7.05] | 0.668 |
| log.sigma.1.5.mm.3D_glrlm_ShortRunEmphasis (median [IQR]) | 0.60 [0.55, 0.65] | 0.59 [0.55, 0.65] | 0.63 [0.56, 0.64] | 0.582 |
| log.sigma.1.5.mm.3D_glrlm_ShortRunHighGrayLevelEmphasis (median [IQR]) | 68.38 [8.44, 112.41] | 77.03 [9.49, 111.04] | 55.71 [5.90, 115.00] | 0.468 |
| log.sigma.1.5.mm.3D_glrlm_ShortRunLowGrayLevelEmphasis (median [IQR]) | 0.01 [0.00, 0.04] | 0.01 [0.00, 0.03] | 0.01 [0.01, 0.06] | 0.468 |
| log.sigma.1.5.mm.3D_glszm_GrayLevelNonUniformity (median [IQR]) | 43.86 [27.16, 105.19] | 52.86 [28.07, 136.83] | 35.79 [24.69, 51.31] | 0.054 |
| log.sigma.1.5.mm.3D_glszm_GrayLevelNonUniformityNormalized (median [IQR]) | 0.17 [0.10, 0.33] | 0.15 [0.10, 0.32] | 0.19 [0.10, 0.41] | 0.75 |
| log.sigma.1.5.mm.3D_glszm_GrayLevelVariance (median [IQR]) | 7.43 [1.54, 10.79] | 7.85 [1.76, 10.59] | 5.59 [1.06, 11.05] | 0.767 |
| log.sigma.1.5.mm.3D_glszm_HighGrayLevelZoneEmphasis (median [IQR]) | 77.56 [16.04, 132.97] | 89.72 [20.17, 132.97] | 77.22 [13.31, 137.13] | 0.575 |
| log.sigma.1.5.mm.3D_glszm_LargeAreaEmphasis (median [IQR]) | 494671.15 [242385.13, 1166193.72] | 615982.90 [242385.13, 1356695.27] | 416526.08 [251140.01, 1052833.42] | 0.403 |
| log.sigma.1.5.mm.3D_glszm_LargeAreaHighGrayLevelEmphasis (median [IQR]) | 34146904.31 [9418016.83, 150538693.71] | 37872255.48 [9820903.47, 150538693.71] | 28829344.28 [7970460.60, 111555662.82] | 0.531 |
| log.sigma.1.5.mm.3D_glszm_LargeAreaLowGrayLevelEmphasis (median [IQR]) | 5160.63 [1886.21, 45484.38] | 4187.12 [1798.16, 46009.24] | 5960.70 [2101.06, 44135.95] | 0.826 |
| log.sigma.1.5.mm.3D_glszm_LowGrayLevelZoneEmphasis (median [IQR]) | 0.03 [0.02, 0.12] | 0.03 [0.02, 0.09] | 0.03 [0.02, 0.13] | 0.652 |
| log.sigma.1.5.mm.3D_glszm_SizeZoneNonUniformity (median [IQR]) | 34.17 [15.87, 109.45] | 34.69 [20.77, 150.44] | 27.86 [10.06, 60.74] | 0.095 |
| log.sigma.1.5.mm.3D_glszm_SizeZoneNonUniformityNormalized (median [IQR]) | 0.16 [0.13, 0.18] | 0.16 [0.13, 0.19] | 0.14 [0.12, 0.17] | 0.121 |
| log.sigma.1.5.mm.3D_glszm_SmallAreaEmphasis (median [IQR]) | 0.40 [0.33, 0.43] | 0.40 [0.35, 0.43] | 0.35 [0.31, 0.41] | 0.027 |
| log.sigma.1.5.mm.3D_glszm_SmallAreaHighGrayLevelEmphasis (median [IQR]) | 31.10 [6.67, 56.57] | 34.19 [6.67, 56.57] | 27.27 [6.60, 54.08] | 0.455 |
| log.sigma.1.5.mm.3D_glszm_SmallAreaLowGrayLevelEmphasis (median [IQR]) | 0.01 [0.01, 0.03] | 0.01 [0.01, 0.03] | 0.01 [0.01, 0.02] | 0.66 |
| log.sigma.1.5.mm.3D_glszm_ZoneEntropy (median [IQR]) | 5.91 [4.90, 6.59] | 5.91 [4.92, 6.59] | 5.94 [4.82, 6.55] | 0.684 |
| log.sigma.1.5.mm.3D_glszm_ZonePercentage (median [IQR]) | 0.02 [0.01, 0.02] | 0.01 [0.01, 0.02] | 0.02 [0.01, 0.02] | 0.717 |
| log.sigma.1.5.mm.3D_glszm_ZoneVariance (median [IQR]) | 472487.07 [233345.81, 1108818.79] | 613760.11 [239406.52, 1326465.04] | 384314.46 [186376.70, 882790.51] | 0.15 |
| log.sigma.1.5.mm.3D_ngtdm_Busyness (median [IQR]) | 12.14 [5.93, 37.36] | 12.14 [6.77, 37.40] | 12.04 [5.48, 33.13] | 0.524 |
| log.sigma.1.5.mm.3D_ngtdm_Coarseness (median [IQR]) | 0.00 [0.00, 0.00] | 0.00 [0.00, 0.00] | 0.00 [0.00, 0.00] | 0.118 |
| log.sigma.1.5.mm.3D_ngtdm_Complexity (median [IQR]) | 38.19 [5.30, 76.63] | 39.06 [6.11, 83.45] | 30.24 [3.49, 72.62] | 0.502 |
| log.sigma.1.5.mm.3D_ngtdm_Contrast (median [IQR]) | 0.01 [0.00, 0.01] | 0.01 [0.00, 0.01] | 0.01 [0.00, 0.01] | 0.826 |
| log.sigma.1.5.mm.3D_ngtdm_Strength (median [IQR]) | 0.07 [0.02, 0.16] | 0.07 [0.03, 0.16] | 0.07 [0.01, 0.17] | 0.86 |
| log.sigma.1.5.mm.3D_gldm_DependenceEntropy (median [IQR]) | 5.81 [5.52, 6.09] | 5.81 [5.52, 6.08] | 5.77 [5.51, 6.11] | 0.792 |
| log.sigma.1.5.mm.3D_gldm_DependenceNonUniformity (median [IQR]) | 855.80 [404.89, 1508.80] | 992.53 [507.82, 1940.13] | 773.52 [342.32, 1206.47] | 0.271 |
| log.sigma.1.5.mm.3D_gldm_DependenceNonUniformityNormalized (median [IQR]) | 0.05 [0.04, 0.05] | 0.05 [0.04, 0.05] | 0.05 [0.04, 0.05] | 0.676 |
| log.sigma.1.5.mm.3D_gldm_DependenceVariance (median [IQR]) | 41.67 [36.89, 46.67] | 42.23 [37.18, 46.87] | 41.07 [36.15, 45.18] | 0.545 |
| log.sigma.1.5.mm.3D_gldm_GrayLevelNonUniformity (median [IQR]) | 8037.83 [4382.92, 15031.69] | 8992.21 [4704.35, 17291.35] | 7053.44 [3642.58, 11732.25] | 0.169 |
| log.sigma.1.5.mm.3D_gldm_GrayLevelVariance (median [IQR]) | 1.08 [0.33, 2.17] | 1.25 [0.36, 2.75] | 0.73 [0.32, 2.02] | 0.385 |
| log.sigma.1.5.mm.3D_gldm_HighGrayLevelEmphasis (median [IQR]) | 115.97 [16.67, 188.86] | 121.17 [16.92, 188.86] | 93.06 [10.53, 185.29] | 0.409 |
| log.sigma.1.5.mm.3D_gldm_LargeDependenceEmphasis (median [IQR]) | 241.26 [212.10, 281.54] | 245.31 [217.73, 282.37] | 229.94 [208.52, 266.21] | 0.391 |
| log.sigma.1.5.mm.3D_gldm_LargeDependenceHighGrayLevelEmphasis (median [IQR]) | 27273.70 [4027.18, 46998.89] | 29546.54 [5026.94, 51432.76] | 22809.74 [2865.76, 38379.28] | 0.253 |
| log.sigma.1.5.mm.3D_gldm_LargeDependenceLowGrayLevelEmphasis (median [IQR]) | 2.10 [0.99, 16.61] | 1.74 [0.99, 15.86] | 2.31 [1.08, 27.50] | 0.531 |
| log.sigma.1.5.mm.3D_gldm_LowGrayLevelEmphasis (median [IQR]) | 0.01 [0.01, 0.07] | 0.01 [0.01, 0.06] | 0.01 [0.01, 0.11] | 0.516 |
| log.sigma.1.5.mm.3D_gldm_SmallDependenceEmphasis (median [IQR]) | 0.02 [0.01, 0.03] | 0.02 [0.01, 0.03] | 0.02 [0.01, 0.02] | 0.775 |
| log.sigma.1.5.mm.3D_gldm_SmallDependenceHighGrayLevelEmphasis (median [IQR]) | 2.12 [0.23, 4.47] | 2.24 [0.24, 4.38] | 1.74 [0.18, 4.52] | 0.502 |
| log.sigma.1.5.mm.3D_gldm_SmallDependenceLowGrayLevelEmphasis (median [IQR]) | 0.00 [0.00, 0.00] | 0.00 [0.00, 0.00] | 0.00 [0.00, 0.00] | 0.676 |
| log.sigma.2.0.mm.3D_firstorder_10Percentile (median [IQR]) | -39.44 [-67.55, -31.49] | -39.44 [-98.98, -30.89] | -39.22 [-66.32, -32.42] | 0.826 |
| log.sigma.2.0.mm.3D_firstorder_90Percentile (median [IQR]) | 0.52 [-1.89, 3.54] | 0.73 [-1.43, 3.61] | -0.32 [-2.60, 3.26] | 0.397 |
| log.sigma.2.0.mm.3D_firstorder_Energy (median [IQR]) | 22409516.11 [4329636.81, 76528533.71] | 30340106.48 [5920057.57, 112486636.44] | 13788309.02 [3312633.71, 65995006.67] | 0.187 |
| log.sigma.2.0.mm.3D_firstorder_Entropy (median [IQR]) | 1.73 [1.33, 2.11] | 1.73 [1.34, 2.12] | 1.72 [1.19, 2.08] | 0.644 |
| log.sigma.2.0.mm.3D_firstorder_InterquartileRange (median [IQR]) | 19.66 [16.46, 25.40] | 19.66 [16.61, 25.40] | 20.31 [16.38, 25.45] | 0.878 |
| log.sigma.2.0.mm.3D_firstorder_Kurtosis (median [IQR]) | 8.35 [3.96, 16.34] | 8.35 [4.04, 16.62] | 8.69 [3.47, 13.99] | 0.416 |
| log.sigma.2.0.mm.3D_firstorder_Maximum (median [IQR]) | 51.01 [32.10, 92.23] | 47.41 [32.60, 92.23] | 59.37 [30.11, 90.07] | 0.886 |
| log.sigma.2.0.mm.3D_firstorder_MeanAbsoluteDeviation (median [IQR]) | 16.22 [10.81, 26.58] | 19.09 [10.81, 33.55] | 15.28 [9.55, 24.49] | 0.553 |
| log.sigma.2.0.mm.3D_firstorder_Mean (median [IQR]) | -20.70 [-32.42, -14.61] | -21.42 [-34.81, -14.68] | -19.54 [-28.82, -13.97] | 0.758 |
| log.sigma.2.0.mm.3D_firstorder_Median (median [IQR]) | -14.28 [-19.55, -10.45] | -13.82 [-19.55, -10.45] | -15.55 [-19.56, -11.06] | 0.545 |
| log.sigma.2.0.mm.3D_firstorder_Minimum (median [IQR]) | -284.82 [-352.67, -73.45] | -310.95 [-355.77, -94.33] | -231.37 [-320.37, -65.11] | 0.166 |
| log.sigma.2.0.mm.3D_firstorder_Range (median [IQR]) | 332.24 [133.29, 423.16] | 339.85 [136.08, 445.38] | 296.73 [101.76, 393.82] | 0.276 |
| log.sigma.2.0.mm.3D_firstorder_RobustMeanAbsoluteDeviation (median [IQR]) | 8.68 [6.96, 11.47] | 8.67 [7.04, 12.41] | 9.02 [6.75, 10.99] | 0.809 |
| log.sigma.2.0.mm.3D_firstorder_RootMeanSquared (median [IQR]) | 38.58 [20.37, 51.63] | 38.61 [20.37, 62.55] | 28.33 [20.77, 48.78] | 0.516 |
| log.sigma.2.0.mm.3D_firstorder_Skewness (median [IQR]) | -2.03 [-3.17, -0.40] | -2.03 [-3.26, -0.48] | -2.13 [-2.83, -0.25] | 0.176 |
| log.sigma.2.0.mm.3D_firstorder_TotalEnergy (median [IQR]) | 50436123.74 [10137719.55, 153131779.43] | 58816691.14 [12792181.13, 189439821.83] | 24604740.72 [6975856.97, 96946964.59] | 0.129 |
| log.sigma.2.0.mm.3D_firstorder_Uniformity (median [IQR]) | 0.41 [0.33, 0.50] | 0.43 [0.33, 0.50] | 0.41 [0.33, 0.51] | 0.733 |
| log.sigma.2.0.mm.3D_firstorder_Variance (median [IQR]) | 928.23 [194.86, 1980.02] | 1014.89 [195.10, 2830.81] | 557.06 [143.67, 1648.48] | 0.306 |
| log.sigma.2.0.mm.3D_glcm_Autocorrelation (median [IQR]) | 123.64 [10.47, 189.48] | 139.34 [15.79, 205.59] | 90.26 [8.82, 165.17] | 0.153 |
| log.sigma.2.0.mm.3D_glcm_ClusterProminence (median [IQR]) | 282.01 [4.73, 1626.66] | 554.07 [4.78, 2025.10] | 150.00 [2.82, 795.71] | 0.159 |
| log.sigma.2.0.mm.3D_glcm_ClusterShade (median [IQR]) | -21.45 [-100.65, -0.32] | -41.36 [-124.96, -0.39] | -14.26 [-57.14, -0.26] | 0.271 |
| log.sigma.2.0.mm.3D_glcm_ClusterTendency (median [IQR]) | 3.96 [1.09, 10.23] | 4.51 [1.10, 14.33] | 2.98 [0.87, 8.05] | 0.276 |
| log.sigma.2.0.mm.3D_glcm_Contrast (median [IQR]) | 0.67 [0.34, 1.26] | 0.71 [0.34, 1.33] | 0.64 [0.35, 1.16] | 0.59 |
| log.sigma.2.0.mm.3D_glcm_Correlation (median [IQR]) | 0.68 [0.53, 0.79] | 0.68 [0.53, 0.79] | 0.69 [0.51, 0.78] | 0.56 |
| log.sigma.2.0.mm.3D_glcm_DifferenceAverage (median [IQR]) | 0.42 [0.31, 0.58] | 0.41 [0.32, 0.60] | 0.46 [0.30, 0.57] | 0.834 |
| log.sigma.2.0.mm.3D_glcm_DifferenceEntropy (median [IQR]) | 1.18 [0.92, 1.39] | 1.14 [0.92, 1.42] | 1.18 [0.92, 1.38] | 0.733 |
| log.sigma.2.0.mm.3D_glcm_DifferenceVariance (median [IQR]) | 0.47 [0.23, 0.89] | 0.48 [0.23, 0.91] | 0.39 [0.23, 0.77] | 0.475 |
| log.sigma.2.0.mm.3D_glcm_Id (median [IQR]) | 0.82 [0.77, 0.85] | 0.82 [0.77, 0.85] | 0.81 [0.77, 0.86] | 0.956 |
| log.sigma.2.0.mm.3D_glcm_Idm (median [IQR]) | 0.81 [0.76, 0.85] | 0.82 [0.76, 0.85] | 0.80 [0.76, 0.86] | 0.965 |
| log.sigma.2.0.mm.3D_glcm_Idmn (median [IQR]) | 1.00 [0.99, 1.00] | 1.00 [0.99, 1.00] | 1.00 [0.99, 1.00] | 0.692 |
| log.sigma.2.0.mm.3D_glcm_Idn (median [IQR]) | 0.97 [0.96, 0.97] | 0.97 [0.96, 0.98] | 0.97 [0.95, 0.97] | 0.56 |
| log.sigma.2.0.mm.3D_glcm_Imc1 (median [IQR]) | -0.26 [-0.30, -0.21] | -0.26 [-0.31, -0.22] | -0.25 [-0.29, -0.21] | 0.194 |
| log.sigma.2.0.mm.3D_glcm_Imc2 (median [IQR]) | 0.70 [0.59, 0.79] | 0.71 [0.60, 0.80] | 0.69 [0.56, 0.77] | 0.244 |
| log.sigma.2.0.mm.3D_glcm_InverseVariance (median [IQR]) | 0.31 [0.27, 0.34] | 0.30 [0.27, 0.33] | 0.32 [0.27, 0.35] | 0.502 |
| log.sigma.2.0.mm.3D_glcm_JointAverage (median [IQR]) | 10.96 [3.21, 13.56] | 11.79 [3.96, 14.17] | 9.46 [2.94, 12.79] | 0.159 |
| log.sigma.2.0.mm.3D_glcm_JointEnergy (median [IQR]) | 0.26 [0.20, 0.34] | 0.27 [0.19, 0.33] | 0.26 [0.21, 0.34] | 0.817 |
| log.sigma.2.0.mm.3D_glcm_JointEntropy (median [IQR]) | 2.82 [2.23, 3.45] | 2.74 [2.29, 3.38] | 2.92 [2.11, 3.47] | 0.676 |
| log.sigma.2.0.mm.3D_glcm_MCC (median [IQR]) | 0.75 [0.56, 0.82] | 0.75 [0.57, 0.84] | 0.75 [0.52, 0.81] | 0.553 |
| log.sigma.2.0.mm.3D_glcm_MaximumProbability (median [IQR]) | 0.46 [0.39, 0.55] | 0.46 [0.38, 0.55] | 0.47 [0.39, 0.54] | 0.725 |
| log.sigma.2.0.mm.3D_glcm_SumAverage (median [IQR]) | 21.92 [6.43, 27.12] | 23.57 [7.93, 28.34] | 18.92 [5.87, 25.57] | 0.159 |
| log.sigma.2.0.mm.3D_glcm_SumEntropy (median [IQR]) | 2.31 [1.85, 2.75] | 2.26 [1.92, 2.72] | 2.34 [1.75, 2.75] | 0.644 |
| log.sigma.2.0.mm.3D_glcm_SumSquares (median [IQR]) | 1.17 [0.37, 2.82] | 1.31 [0.37, 3.85] | 0.88 [0.29, 2.25] | 0.301 |
| log.sigma.2.0.mm.3D_glrlm_GrayLevelNonUniformity (median [IQR]) | 2715.26 [1441.37, 4505.39] | 2747.61 [1740.04, 5136.69] | 2431.00 [1023.61, 3571.45] | 0.271 |
| log.sigma.2.0.mm.3D_glrlm_GrayLevelNonUniformityNormalized (median [IQR]) | 0.30 [0.24, 0.40] | 0.29 [0.22, 0.39] | 0.31 [0.25, 0.41] | 0.56 |
| log.sigma.2.0.mm.3D_glrlm_GrayLevelVariance (median [IQR]) | 2.40 [0.52, 5.93] | 2.69 [0.52, 6.76] | 1.58 [0.45, 4.03] | 0.206 |
| log.sigma.2.0.mm.3D_glrlm_HighGrayLevelRunEmphasis (median [IQR]) | 110.50 [10.55, 177.58] | 132.65 [16.15, 182.23] | 87.39 [8.61, 156.13] | 0.153 |
| log.sigma.2.0.mm.3D_glrlm_LongRunEmphasis (median [IQR]) | 14.96 [10.99, 20.45] | 15.36 [11.59, 21.59] | 14.37 [10.49, 19.47] | 0.56 |
| log.sigma.2.0.mm.3D_glrlm_LongRunHighGrayLevelEmphasis (median [IQR]) | 1467.03 [224.52, 2494.71] | 1869.25 [363.06, 2968.98] | 1244.10 [143.64, 1650.59] | 0.097 |
| log.sigma.2.0.mm.3D_glrlm_LongRunLowGrayLevelEmphasis (median [IQR]) | 0.11 [0.06, 1.66] | 0.10 [0.06, 1.16] | 0.17 [0.08, 1.83] | 0.361 |
| log.sigma.2.0.mm.3D_glrlm_LowGrayLevelRunEmphasis (median [IQR]) | 0.01 [0.01, 0.11] | 0.01 [0.01, 0.07] | 0.01 [0.01, 0.16] | 0.271 |
| log.sigma.2.0.mm.3D_glrlm_RunEntropy (median [IQR]) | 4.10 [3.80, 4.48] | 4.13 [3.81, 4.53] | 3.96 [3.66, 4.34] | 0.257 |
| log.sigma.2.0.mm.3D_glrlm_RunLengthNonUniformity (median [IQR]) | 3078.89 [1475.37, 7933.44] | 3694.45 [1662.34, 9677.91] | 2789.66 [1392.28, 5602.41] | 0.248 |
| log.sigma.2.0.mm.3D_glrlm_RunLengthNonUniformityNormalized (median [IQR]) | 0.36 [0.31, 0.43] | 0.35 [0.30, 0.43] | 0.39 [0.33, 0.43] | 0.538 |
| log.sigma.2.0.mm.3D_glrlm_RunPercentage (median [IQR]) | 0.49 [0.42, 0.54] | 0.48 [0.42, 0.54] | 0.50 [0.45, 0.55] | 0.482 |
| log.sigma.2.0.mm.3D_glrlm_RunVariance (median [IQR]) | 7.74 [5.48, 10.47] | 8.13 [5.59, 10.80] | 7.53 [4.69, 10.01] | 0.409 |
| log.sigma.2.0.mm.3D_glrlm_ShortRunEmphasis (median [IQR]) | 0.60 [0.53, 0.66] | 0.58 [0.53, 0.66] | 0.63 [0.55, 0.65] | 0.692 |
| log.sigma.2.0.mm.3D_glrlm_ShortRunHighGrayLevelEmphasis (median [IQR]) | 73.68 [5.72, 111.14] | 81.24 [8.60, 115.36] | 52.55 [5.06, 98.30] | 0.239 |
| log.sigma.2.0.mm.3D_glrlm_ShortRunLowGrayLevelEmphasis (median [IQR]) | 0.01 [0.01, 0.06] | 0.01 [0.01, 0.04] | 0.01 [0.01, 0.09] | 0.231 |
| log.sigma.2.0.mm.3D_glszm_GrayLevelNonUniformity (median [IQR]) | 36.92 [20.65, 79.94] | 41.90 [21.89, 97.44] | 33.30 [16.88, 46.18] | 0.136 |
| log.sigma.2.0.mm.3D_glszm_GrayLevelNonUniformityNormalized (median [IQR]) | 0.16 [0.10, 0.33] | 0.13 [0.10, 0.28] | 0.19 [0.11, 0.38] | 0.385 |
| log.sigma.2.0.mm.3D_glszm_GrayLevelVariance (median [IQR]) | 8.37 [1.50, 12.64] | 9.13 [1.79, 12.64] | 6.22 [1.20, 11.84] | 0.296 |
| log.sigma.2.0.mm.3D_glszm_HighGrayLevelZoneEmphasis (median [IQR]) | 85.74 [15.45, 140.38] | 88.07 [19.62, 142.12] | 83.65 [14.07, 129.07] | 0.344 |
| log.sigma.2.0.mm.3D_glszm_LargeAreaEmphasis (median [IQR]) | 528846.17 [235638.27, 1530587.28] | 568821.37 [235638.27, 1720678.19] | 524349.52 [317605.63, 903405.57] | 0.545 |
| log.sigma.2.0.mm.3D_glszm_LargeAreaHighGrayLevelEmphasis (median [IQR]) | 37633724.14 [9451859.45, 146797704.63] | 42985452.07 [11714337.05, 164117181.80] | 30303852.29 [7876166.43, 106116786.43] | 0.361 |
| log.sigma.2.0.mm.3D_glszm_LargeAreaLowGrayLevelEmphasis (median [IQR]) | 6791.81 [2212.09, 37901.51] | 6343.07 [1724.27, 37409.37] | 7945.83 [2751.38, 55221.26] | 0.613 |
| log.sigma.2.0.mm.3D_glszm_LowGrayLevelZoneEmphasis (median [IQR]) | 0.03 [0.02, 0.12] | 0.03 [0.02, 0.09] | 0.03 [0.02, 0.13] | 0.397 |
| log.sigma.2.0.mm.3D_glszm_SizeZoneNonUniformity (median [IQR]) | 27.90 [10.63, 88.16] | 28.82 [11.65, 116.20] | 26.13 [9.88, 55.68] | 0.194 |
| log.sigma.2.0.mm.3D_glszm_SizeZoneNonUniformityNormalized (median [IQR]) | 0.15 [0.12, 0.17] | 0.15 [0.12, 0.17] | 0.15 [0.11, 0.20] | 0.725 |
| log.sigma.2.0.mm.3D_glszm_SmallAreaEmphasis (median [IQR]) | 0.38 [0.33, 0.41] | 0.38 [0.34, 0.41] | 0.36 [0.29, 0.44] | 0.725 |
| log.sigma.2.0.mm.3D_glszm_SmallAreaHighGrayLevelEmphasis (median [IQR]) | 32.17 [6.77, 59.33] | 33.60 [7.97, 62.45] | 30.60 [5.55, 50.05] | 0.339 |
| log.sigma.2.0.mm.3D_glszm_SmallAreaLowGrayLevelEmphasis (median [IQR]) | 0.01 [0.01, 0.02] | 0.01 [0.01, 0.02] | 0.01 [0.01, 0.02] | 0.852 |
| log.sigma.2.0.mm.3D_glszm_ZoneEntropy (median [IQR]) | 6.11 [4.96, 6.71] | 6.25 [5.15, 6.81] | 5.91 [4.43, 6.39] | 0.111 |
| log.sigma.2.0.mm.3D_glszm_ZonePercentage (median [IQR]) | 0.01 [0.01, 0.02] | 0.01 [0.01, 0.02] | 0.01 [0.01, 0.02] | 0.636 |
| log.sigma.2.0.mm.3D_glszm_ZoneVariance (median [IQR]) | 506239.65 [225412.77, 1222492.96] | 566099.86 [225412.77, 1685742.68] | 502223.60 [210295.89, 788482.17] | 0.231 |
| log.sigma.2.0.mm.3D_ngtdm_Busyness (median [IQR]) | 10.75 [5.55, 25.10] | 10.09 [5.68, 25.10] | 11.85 [4.83, 24.77] | 0.758 |
| log.sigma.2.0.mm.3D_ngtdm_Coarseness (median [IQR]) | 0.00 [0.00, 0.00] | 0.00 [0.00, 0.00] | 0.00 [0.00, 0.00] | 0.104 |
| log.sigma.2.0.mm.3D_ngtdm_Complexity (median [IQR]) | 47.58 [4.96, 78.54] | 52.70 [5.63, 84.06] | 30.71 [3.42, 62.82] | 0.379 |
| log.sigma.2.0.mm.3D_ngtdm_Contrast (median [IQR]) | 0.01 [0.00, 0.01] | 0.01 [0.00, 0.01] | 0.01 [0.01, 0.01] | 0.75 |
| log.sigma.2.0.mm.3D_ngtdm_Strength (median [IQR]) | 0.08 [0.02, 0.18] | 0.08 [0.03, 0.18] | 0.07 [0.02, 0.16] | 0.575 |
| log.sigma.2.0.mm.3D_gldm_DependenceEntropy (median [IQR]) | 5.91 [5.61, 6.31] | 5.87 [5.61, 6.34] | 5.93 [5.55, 6.20] | 0.725 |
| log.sigma.2.0.mm.3D_gldm_DependenceNonUniformity (median [IQR]) | 903.94 [417.73, 1699.04] | 1087.81 [532.73, 1975.26] | 808.26 [324.11, 1132.15] | 0.111 |
| log.sigma.2.0.mm.3D_gldm_DependenceNonUniformityNormalized (median [IQR]) | 0.05 [0.05, 0.05] | 0.05 [0.05, 0.05] | 0.05 [0.05, 0.05] | 0.912 |
| log.sigma.2.0.mm.3D_gldm_DependenceVariance (median [IQR]) | 44.33 [39.25, 49.97] | 44.75 [40.67, 50.93] | 43.14 [38.07, 47.60] | 0.235 |
| log.sigma.2.0.mm.3D_gldm_GrayLevelNonUniformity (median [IQR]) | 7394.00 [4167.25, 14177.79] | 7791.38 [4402.28, 16504.11] | 6253.85 [3514.80, 11777.80] | 0.206 |
| log.sigma.2.0.mm.3D_gldm_GrayLevelVariance (median [IQR]) | 1.56 [0.38, 3.26] | 1.69 [0.39, 4.58] | 0.98 [0.31, 2.70] | 0.291 |
| log.sigma.2.0.mm.3D_gldm_HighGrayLevelEmphasis (median [IQR]) | 121.94 [10.42, 186.69] | 138.80 [15.91, 202.50] | 89.77 [8.77, 164.18] | 0.147 |
| log.sigma.2.0.mm.3D_gldm_LargeDependenceEmphasis (median [IQR]) | 250.15 [215.89, 306.93] | 251.66 [216.52, 307.87] | 242.11 [196.79, 290.41] | 0.502 |
| log.sigma.2.0.mm.3D_gldm_LargeDependenceHighGrayLevelEmphasis (median [IQR]) | 27354.02 [3010.51, 47885.81] | 35372.57 [5223.31, 56957.19] | 21953.29 [2383.76, 34732.15] | 0.097 |
| log.sigma.2.0.mm.3D_gldm_LargeDependenceLowGrayLevelEmphasis (median [IQR]) | 1.88 [1.05, 28.04] | 1.61 [0.99, 18.99] | 2.58 [1.33, 30.11] | 0.267 |
| log.sigma.2.0.mm.3D_gldm_LowGrayLevelEmphasis (median [IQR]) | 0.01 [0.01, 0.11] | 0.01 [0.01, 0.07] | 0.01 [0.01, 0.14] | 0.244 |
| log.sigma.2.0.mm.3D_gldm_SmallDependenceEmphasis (median [IQR]) | 0.02 [0.01, 0.03] | 0.02 [0.01, 0.03] | 0.02 [0.01, 0.02] | 0.826 |
| log.sigma.2.0.mm.3D_gldm_SmallDependenceHighGrayLevelEmphasis (median [IQR]) | 2.19 [0.21, 4.40] | 2.45 [0.22, 4.68] | 1.57 [0.17, 3.62] | 0.257 |
| log.sigma.2.0.mm.3D_gldm_SmallDependenceLowGrayLevelEmphasis (median [IQR]) | 0.00 [0.00, 0.00] | 0.00 [0.00, 0.00] | 0.00 [0.00, 0.00] | 0.428 |
| log.sigma.2.5.mm.3D_firstorder_10Percentile (median [IQR]) | -47.11 [-101.02, -34.57] | -47.78 [-121.85, -34.51] | -45.61 [-88.23, -36.24] | 0.621 |
| log.sigma.2.5.mm.3D_firstorder_90Percentile (median [IQR]) | -1.34 [-5.21, 1.75] | -1.12 [-5.07, 2.16] | -3.31 [-6.03, 1.02] | 0.312 |
| log.sigma.2.5.mm.3D_firstorder_Energy (median [IQR]) | 28828429.84 [5646471.40, 119525879.55] | 39583964.81 [8196080.24, 149710323.29] | 17360466.63 [3913803.17, 84413309.38] | 0.156 |
| log.sigma.2.5.mm.3D_firstorder_Entropy (median [IQR]) | 1.88 [1.35, 2.31] | 1.88 [1.38, 2.36] | 1.89 [1.27, 2.21] | 0.516 |
| log.sigma.2.5.mm.3D_firstorder_InterquartileRange (median [IQR]) | 22.12 [16.74, 29.31] | 21.87 [16.63, 30.30] | 22.74 [17.25, 27.65] | 0.783 |
| log.sigma.2.5.mm.3D_firstorder_Kurtosis (median [IQR]) | 6.63 [3.41, 11.43] | 6.34 [3.41, 11.58] | 7.31 [3.58, 10.01] | 0.575 |
| log.sigma.2.5.mm.3D_firstorder_Maximum (median [IQR]) | 53.75 [34.15, 100.74] | 53.22 [35.92, 100.74] | 55.14 [24.44, 93.52] | 0.692 |
| log.sigma.2.5.mm.3D_firstorder_MeanAbsoluteDeviation (median [IQR]) | 18.12 [11.04, 34.01] | 21.55 [11.71, 39.79] | 17.54 [9.98, 28.40] | 0.361 |
| log.sigma.2.5.mm.3D_firstorder_Mean (median [IQR]) | -26.43 [-39.84, -17.67] | -26.69 [-43.77, -17.67] | -22.75 [-36.28, -17.86] | 0.717 |
| log.sigma.2.5.mm.3D_firstorder_Median (median [IQR]) | -18.16 [-27.03, -13.76] | -17.73 [-27.25, -13.76] | -19.55 [-24.83, -14.31] | 0.66 |
| log.sigma.2.5.mm.3D_firstorder_Minimum (median [IQR]) | -260.52 [-338.95, -75.77] | -293.78 [-355.00, -80.01] | -208.59 [-301.92, -58.52] | 0.095 |
| log.sigma.2.5.mm.3D_firstorder_Range (median [IQR]) | 316.13 [129.00, 424.66] | 354.50 [154.01, 446.71] | 289.44 [100.87, 381.21] | 0.18 |
| log.sigma.2.5.mm.3D_firstorder_RobustMeanAbsoluteDeviation (median [IQR]) | 9.69 [7.08, 14.34] | 9.69 [7.08, 17.05] | 10.14 [7.25, 13.45] | 0.717 |
| log.sigma.2.5.mm.3D_firstorder_RootMeanSquared (median [IQR]) | 43.21 [23.04, 62.52] | 44.56 [22.61, 72.99] | 31.56 [24.06, 56.66] | 0.428 |
| log.sigma.2.5.mm.3D_firstorder_Skewness (median [IQR]) | -1.73 [-2.59, -0.31] | -1.73 [-2.78, -0.35] | -1.76 [-2.43, -0.06] | 0.173 |
| log.sigma.2.5.mm.3D_firstorder_TotalEnergy (median [IQR]) | 63422287.87 [12124704.07, 203316759.65] | 77968946.89 [16712724.15, 257371566.26] | 30982748.80 [8585756.59, 122741910.80] | 0.116 |
| log.sigma.2.5.mm.3D_firstorder_Uniformity (median [IQR]) | 0.39 [0.30, 0.48] | 0.40 [0.30, 0.48] | 0.38 [0.31, 0.47] | 0.852 |
| log.sigma.2.5.mm.3D_firstorder_Variance (median [IQR]) | 1120.76 [202.36, 2725.34] | 1247.23 [213.39, 3567.79] | 687.84 [157.67, 1895.41] | 0.206 |
| log.sigma.2.5.mm.3D_glcm_Autocorrelation (median [IQR]) | 111.74 [12.41, 171.02] | 118.14 [16.67, 171.60] | 75.52 [8.75, 137.77] | 0.135 |
| log.sigma.2.5.mm.3D_glcm_ClusterProminence (median [IQR]) | 297.39 [5.06, 2114.10] | 644.68 [5.58, 2536.35] | 189.41 [2.81, 828.00] | 0.135 |
| log.sigma.2.5.mm.3D_glcm_ClusterShade (median [IQR]) | -26.29 [-126.05, -0.27] | -47.01 [-153.54, -0.37] | -18.63 [-67.89, -0.18] | 0.135 |
| log.sigma.2.5.mm.3D_glcm_ClusterTendency (median [IQR]) | 4.94 [1.21, 14.60] | 6.49 [1.27, 19.32] | 3.91 [0.95, 10.55] | 0.222 |
| log.sigma.2.5.mm.3D_glcm_Contrast (median [IQR]) | 0.64 [0.32, 1.24] | 0.66 [0.32, 1.27] | 0.57 [0.33, 1.03] | 0.516 |
| log.sigma.2.5.mm.3D_glcm_Correlation (median [IQR]) | 0.76 [0.58, 0.84] | 0.78 [0.59, 0.86] | 0.76 [0.57, 0.82] | 0.502 |
| log.sigma.2.5.mm.3D_glcm_DifferenceAverage (median [IQR]) | 0.42 [0.30, 0.59] | 0.41 [0.30, 0.64] | 0.44 [0.31, 0.59] | 0.717 |
| log.sigma.2.5.mm.3D_glcm_DifferenceEntropy (median [IQR]) | 1.12 [0.89, 1.39] | 1.12 [0.89, 1.49] | 1.13 [0.90, 1.35] | 0.59 |
| log.sigma.2.5.mm.3D_glcm_DifferenceVariance (median [IQR]) | 0.43 [0.22, 0.79] | 0.45 [0.22, 0.86] | 0.35 [0.22, 0.67] | 0.416 |
| log.sigma.2.5.mm.3D_glcm_Id (median [IQR]) | 0.82 [0.77, 0.85] | 0.82 [0.77, 0.85] | 0.81 [0.77, 0.85] | 0.912 |
| log.sigma.2.5.mm.3D_glcm_Idm (median [IQR]) | 0.81 [0.76, 0.85] | 0.82 [0.75, 0.85] | 0.80 [0.76, 0.85] | 0.878 |
| log.sigma.2.5.mm.3D_glcm_Idmn (median [IQR]) | 1.00 [0.99, 1.00] | 1.00 [0.99, 1.00] | 1.00 [0.99, 1.00] | 0.767 |
| log.sigma.2.5.mm.3D_glcm_Idn (median [IQR]) | 0.97 [0.96, 0.98] | 0.97 [0.96, 0.98] | 0.97 [0.96, 0.97] | 0.59 |
| log.sigma.2.5.mm.3D_glcm_Imc1 (median [IQR]) | -0.31 [-0.35, -0.25] | -0.31 [-0.36, -0.26] | -0.30 [-0.34, -0.24] | 0.244 |
| log.sigma.2.5.mm.3D_glcm_Imc2 (median [IQR]) | 0.77 [0.65, 0.86] | 0.80 [0.65, 0.87] | 0.76 [0.63, 0.84] | 0.231 |
| log.sigma.2.5.mm.3D_glcm_InverseVariance (median [IQR]) | 0.30 [0.26, 0.33] | 0.30 [0.26, 0.33] | 0.31 [0.26, 0.34] | 0.545 |
| log.sigma.2.5.mm.3D_glcm_JointAverage (median [IQR]) | 10.54 [3.48, 12.80] | 10.79 [4.07, 13.06] | 8.66 [2.93, 11.61] | 0.138 |
| log.sigma.2.5.mm.3D_glcm_JointEnergy (median [IQR]) | 0.25 [0.17, 0.33] | 0.25 [0.17, 0.34] | 0.24 [0.18, 0.32] | 0.939 |
| log.sigma.2.5.mm.3D_glcm_JointEntropy (median [IQR]) | 3.00 [2.34, 3.69] | 2.95 [2.35, 3.73] | 3.04 [2.18, 3.65] | 0.636 |
| log.sigma.2.5.mm.3D_glcm_MCC (median [IQR]) | 0.81 [0.62, 0.88] | 0.82 [0.62, 0.88] | 0.80 [0.59, 0.85] | 0.582 |
| log.sigma.2.5.mm.3D_glcm_MaximumProbability (median [IQR]) | 0.43 [0.33, 0.52] | 0.43 [0.33, 0.52] | 0.42 [0.32, 0.50] | 0.834 |
| log.sigma.2.5.mm.3D_glcm_SumAverage (median [IQR]) | 21.07 [6.96, 25.60] | 21.59 [8.15, 26.12] | 17.32 [5.86, 23.22] | 0.138 |
| log.sigma.2.5.mm.3D_glcm_SumEntropy (median [IQR]) | 2.44 [1.99, 2.98] | 2.44 [2.02, 3.00] | 2.51 [1.81, 2.91] | 0.567 |
| log.sigma.2.5.mm.3D_glcm_SumSquares (median [IQR]) | 1.42 [0.39, 3.89] | 1.79 [0.42, 5.19] | 1.11 [0.32, 2.89] | 0.239 |
| log.sigma.2.5.mm.3D_glrlm_GrayLevelNonUniformity (median [IQR]) | 2506.73 [1433.32, 4058.55] | 2553.23 [1490.73, 4387.02] | 2201.65 [996.21, 3306.17] | 0.339 |
| log.sigma.2.5.mm.3D_glrlm_GrayLevelNonUniformityNormalized (median [IQR]) | 0.27 [0.20, 0.40] | 0.26 [0.19, 0.38] | 0.28 [0.22, 0.42] | 0.373 |
| log.sigma.2.5.mm.3D_glrlm_GrayLevelVariance (median [IQR]) | 2.71 [0.52, 6.79] | 3.14 [0.54, 7.59] | 1.82 [0.45, 4.39] | 0.108 |
| log.sigma.2.5.mm.3D_glrlm_HighGrayLevelRunEmphasis (median [IQR]) | 108.67 [13.44, 155.00] | 115.14 [17.13, 166.69] | 74.12 [9.32, 128.78] | 0.138 |
| log.sigma.2.5.mm.3D_glrlm_LongRunEmphasis (median [IQR]) | 16.21 [10.54, 22.12] | 16.21 [11.02, 24.02] | 16.15 [10.39, 19.74] | 0.509 |
| log.sigma.2.5.mm.3D_glrlm_LongRunHighGrayLevelEmphasis (median [IQR]) | 1139.87 [326.89, 2399.14] | 1574.84 [434.78, 2509.65] | 1096.22 [141.97, 1494.29] | 0.108 |
| log.sigma.2.5.mm.3D_glrlm_LongRunLowGrayLevelEmphasis (median [IQR]) | 0.16 [0.08, 1.98] | 0.14 [0.06, 1.93] | 0.22 [0.08, 2.56] | 0.356 |
| log.sigma.2.5.mm.3D_glrlm_LowGrayLevelRunEmphasis (median [IQR]) | 0.01 [0.01, 0.10] | 0.01 [0.01, 0.07] | 0.02 [0.01, 0.13] | 0.21 |
| log.sigma.2.5.mm.3D_glrlm_RunEntropy (median [IQR]) | 4.23 [3.86, 4.64] | 4.30 [3.91, 4.68] | 4.10 [3.74, 4.50] | 0.191 |
| log.sigma.2.5.mm.3D_glrlm_RunLengthNonUniformity (median [IQR]) | 3170.13 [1408.19, 8385.39] | 3361.75 [1515.12, 9064.53] | 2573.66 [1352.52, 5418.80] | 0.231 |
| log.sigma.2.5.mm.3D_glrlm_RunLengthNonUniformityNormalized (median [IQR]) | 0.37 [0.30, 0.43] | 0.36 [0.30, 0.44] | 0.40 [0.29, 0.43] | 0.912 |
| log.sigma.2.5.mm.3D_glrlm_RunPercentage (median [IQR]) | 0.48 [0.41, 0.56] | 0.48 [0.41, 0.54] | 0.51 [0.43, 0.56] | 0.684 |
| log.sigma.2.5.mm.3D_glrlm_RunVariance (median [IQR]) | 7.92 [5.52, 11.98] | 8.40 [5.89, 12.57] | 6.74 [5.16, 10.66] | 0.317 |
| log.sigma.2.5.mm.3D_glrlm_ShortRunEmphasis (median [IQR]) | 0.60 [0.53, 0.67] | 0.59 [0.53, 0.67] | 0.63 [0.52, 0.65] | 0.974 |
| log.sigma.2.5.mm.3D_glrlm_ShortRunHighGrayLevelEmphasis (median [IQR]) | 62.78 [7.58, 100.93] | 66.05 [8.18, 113.89] | 40.43 [5.05, 83.62] | 0.202 |
| log.sigma.2.5.mm.3D_glrlm_ShortRunLowGrayLevelEmphasis (median [IQR]) | 0.01 [0.01, 0.05] | 0.01 [0.01, 0.03] | 0.01 [0.01, 0.07] | 0.214 |
| log.sigma.2.5.mm.3D_glszm_GrayLevelNonUniformity (median [IQR]) | 32.84 [15.92, 63.79] | 33.00 [18.23, 79.45] | 25.12 [14.06, 42.62] | 0.122 |
| log.sigma.2.5.mm.3D_glszm_GrayLevelNonUniformityNormalized (median [IQR]) | 0.15 [0.10, 0.33] | 0.14 [0.09, 0.32] | 0.17 [0.12, 0.38] | 0.306 |
| log.sigma.2.5.mm.3D_glszm_GrayLevelVariance (median [IQR]) | 7.84 [1.32, 12.44] | 8.77 [1.69, 12.77] | 5.75 [0.91, 10.65] | 0.153 |
| log.sigma.2.5.mm.3D_glszm_HighGrayLevelZoneEmphasis (median [IQR]) | 91.73 [20.62, 131.47] | 100.21 [21.54, 140.19] | 82.11 [15.49, 124.49] | 0.317 |
| log.sigma.2.5.mm.3D_glszm_LargeAreaEmphasis (median [IQR]) | 599502.87 [232486.90, 1597113.63] | 609995.35 [232486.90, 2150847.40] | 586901.50 [319493.27, 948012.22] | 0.502 |
| log.sigma.2.5.mm.3D_glszm_LargeAreaHighGrayLevelEmphasis (median [IQR]) | 40994919.19 [9454374.01, 132739437.15] | 45713486.20 [17248431.71, 137175215.87] | 29841647.25 [6257999.63, 104996713.69] | 0.322 |
| log.sigma.2.5.mm.3D_glszm_LargeAreaLowGrayLevelEmphasis (median [IQR]) | 8258.74 [2211.13, 55835.24] | 8039.45 [2113.14, 55835.24] | 10280.49 [3119.06, 63900.87] | 0.7 |
| log.sigma.2.5.mm.3D_glszm_LowGrayLevelZoneEmphasis (median [IQR]) | 0.03 [0.02, 0.09] | 0.03 [0.02, 0.07] | 0.03 [0.02, 0.10] | 0.367 |
| log.sigma.2.5.mm.3D_glszm_SizeZoneNonUniformity (median [IQR]) | 25.55 [8.22, 76.23] | 30.18 [10.35, 100.60] | 20.35 [6.98, 58.00] | 0.147 |
| log.sigma.2.5.mm.3D_glszm_SizeZoneNonUniformityNormalized (median [IQR]) | 0.16 [0.13, 0.18] | 0.16 [0.13, 0.18] | 0.15 [0.12, 0.18] | 0.652 |
| log.sigma.2.5.mm.3D_glszm_SmallAreaEmphasis (median [IQR]) | 0.39 [0.34, 0.43] | 0.39 [0.35, 0.43] | 0.36 [0.32, 0.44] | 0.235 |
| log.sigma.2.5.mm.3D_glszm_SmallAreaHighGrayLevelEmphasis (median [IQR]) | 40.23 [7.34, 56.20] | 40.75 [8.86, 55.33] | 29.77 [5.35, 57.06] | 0.35 |
| log.sigma.2.5.mm.3D_glszm_SmallAreaLowGrayLevelEmphasis (median [IQR]) | 0.01 [0.00, 0.02] | 0.01 [0.00, 0.02] | 0.01 [0.01, 0.02] | 0.974 |
| log.sigma.2.5.mm.3D_glszm_ZoneEntropy (median [IQR]) | 5.91 [4.72, 6.48] | 5.99 [4.92, 6.59] | 5.59 [4.38, 6.23] | 0.144 |
| log.sigma.2.5.mm.3D_glszm_ZonePercentage (median [IQR]) | 0.01 [0.01, 0.02] | 0.01 [0.01, 0.02] | 0.01 [0.01, 0.01] | 0.567 |
| log.sigma.2.5.mm.3D_glszm_ZoneVariance (median [IQR]) | 585815.10 [219749.29, 1503303.73] | 595192.16 [222712.54, 2104650.41] | 536332.93 [205013.03, 753792.17] | 0.214 |
| log.sigma.2.5.mm.3D_ngtdm_Busyness (median [IQR]) | 9.33 [5.07, 30.27] | 9.33 [4.82, 30.27] | 9.52 [5.88, 25.01] | 0.834 |
| log.sigma.2.5.mm.3D_ngtdm_Coarseness (median [IQR]) | 0.00 [0.00, 0.00] | 0.00 [0.00, 0.00] | 0.00 [0.00, 0.00] | 0.124 |
| log.sigma.2.5.mm.3D_ngtdm_Complexity (median [IQR]) | 34.83 [4.93, 68.05] | 41.75 [6.20, 70.51] | 24.75 [3.17, 56.26] | 0.21 |
| log.sigma.2.5.mm.3D_ngtdm_Contrast (median [IQR]) | 0.01 [0.00, 0.01] | 0.01 [0.00, 0.02] | 0.01 [0.01, 0.01] | 0.904 |
| log.sigma.2.5.mm.3D_ngtdm_Strength (median [IQR]) | 0.08 [0.02, 0.15] | 0.09 [0.02, 0.17] | 0.07 [0.02, 0.13] | 0.553 |
| log.sigma.2.5.mm.3D_gldm_DependenceEntropy (median [IQR]) | 5.99 [5.65, 6.48] | 5.94 [5.67, 6.50] | 6.02 [5.64, 6.37] | 0.767 |
| log.sigma.2.5.mm.3D_gldm_DependenceNonUniformity (median [IQR]) | 1104.31 [443.05, 1942.07] | 1166.29 [540.88, 2043.10] | 902.51 [394.53, 1479.40] | 0.235 |
| log.sigma.2.5.mm.3D_gldm_DependenceNonUniformityNormalized (median [IQR]) | 0.05 [0.05, 0.06] | 0.05 [0.05, 0.06] | 0.05 [0.05, 0.06] | 0.582 |
| log.sigma.2.5.mm.3D_gldm_DependenceVariance (median [IQR]) | 44.19 [39.71, 49.40] | 44.99 [40.51, 50.21] | 42.01 [37.72, 47.45] | 0.202 |
| log.sigma.2.5.mm.3D_gldm_GrayLevelNonUniformity (median [IQR]) | 6967.50 [3737.65, 12499.82] | 7104.37 [4154.38, 14179.26] | 5652.29 [2909.71, 10942.01] | 0.262 |
| log.sigma.2.5.mm.3D_gldm_GrayLevelVariance (median [IQR]) | 1.88 [0.40, 4.39] | 2.11 [0.42, 5.78] | 1.19 [0.34, 3.11] | 0.235 |
| log.sigma.2.5.mm.3D_gldm_HighGrayLevelEmphasis (median [IQR]) | 111.72 [13.10, 169.79] | 120.99 [16.88, 173.16] | 75.60 [8.96, 137.88] | 0.129 |
| log.sigma.2.5.mm.3D_gldm_LargeDependenceEmphasis (median [IQR]) | 254.90 [206.24, 312.87] | 257.30 [208.67, 313.30] | 233.32 [198.98, 296.53] | 0.582 |
| log.sigma.2.5.mm.3D_gldm_LargeDependenceHighGrayLevelEmphasis (median [IQR]) | 24198.93 [3677.97, 42703.01] | 29973.72 [5045.40, 49478.00] | 19877.92 [2424.53, 30761.43] | 0.129 |
| log.sigma.2.5.mm.3D_gldm_LargeDependenceLowGrayLevelEmphasis (median [IQR]) | 2.20 [1.21, 22.67] | 2.00 [1.01, 21.61] | 3.63 [1.40, 37.86] | 0.206 |
| log.sigma.2.5.mm.3D_gldm_LowGrayLevelEmphasis (median [IQR]) | 0.01 [0.01, 0.09] | 0.01 [0.01, 0.06] | 0.02 [0.01, 0.13] | 0.166 |
| log.sigma.2.5.mm.3D_gldm_SmallDependenceEmphasis (median [IQR]) | 0.02 [0.01, 0.03] | 0.02 [0.01, 0.03] | 0.02 [0.01, 0.02] | 0.775 |
| log.sigma.2.5.mm.3D_gldm_SmallDependenceHighGrayLevelEmphasis (median [IQR]) | 1.94 [0.18, 4.06] | 2.15 [0.21, 4.17] | 1.16 [0.14, 3.31] | 0.257 |
| log.sigma.2.5.mm.3D_gldm_SmallDependenceLowGrayLevelEmphasis (median [IQR]) | 0.00 [0.00, 0.00] | 0.00 [0.00, 0.00] | 0.00 [0.00, 0.00] | 0.495 |
| log.sigma.3.0.mm.3D_firstorder_10Percentile (median [IQR]) | -53.58 [-124.85, -38.82] | -55.64 [-141.03, -35.58] | -50.05 [-102.46, -39.25] | 0.422 |
| log.sigma.3.0.mm.3D_firstorder_90Percentile (median [IQR]) | -3.96 [-7.79, 0.00] | -2.63 [-7.02, 0.84] | -4.99 [-8.71, -0.65] | 0.267 |
| log.sigma.3.0.mm.3D_firstorder_Energy (median [IQR]) | 34819509.22 [7364994.67, 145809024.87] | 45054716.27 [10136321.70, 176897511.88] | 20181767.24 [4606108.57, 99809941.42] | 0.153 |
| log.sigma.3.0.mm.3D_firstorder_Entropy (median [IQR]) | 2.05 [1.38, 2.47] | 2.05 [1.39, 2.59] | 2.02 [1.26, 2.34] | 0.428 |
| log.sigma.3.0.mm.3D_firstorder_InterquartileRange (median [IQR]) | 24.02 [17.58, 36.64] | 23.82 [17.25, 44.11] | 26.30 [18.21, 31.99] | 0.613 |
| log.sigma.3.0.mm.3D_firstorder_Kurtosis (median [IQR]) | 6.21 [3.31, 8.95] | 6.21 [3.31, 9.02] | 5.92 [3.42, 8.51] | 0.502 |
| log.sigma.3.0.mm.3D_firstorder_Maximum (median [IQR]) | 63.39 [32.73, 122.07] | 63.39 [32.73, 111.00] | 59.99 [30.64, 131.26] | 0.636 |
| log.sigma.3.0.mm.3D_firstorder_MeanAbsoluteDeviation (median [IQR]) | 20.15 [11.16, 38.52] | 24.54 [11.31, 43.76] | 19.58 [10.71, 30.92] | 0.226 |
| log.sigma.3.0.mm.3D_firstorder_Mean (median [IQR]) | -30.96 [-44.92, -19.92] | -31.41 [-52.43, -19.92] | -26.39 [-42.48, -21.22] | 0.621 |
| log.sigma.3.0.mm.3D_firstorder_Median (median [IQR]) | -21.65 [-31.69, -16.36] | -21.18 [-33.09, -16.36] | -22.69 [-28.70, -16.97] | 0.904 |
| log.sigma.3.0.mm.3D_firstorder_Minimum (median [IQR]) | -242.69 [-349.89, -75.17] | -270.04 [-353.75, -90.14] | -183.53 [-303.86, -62.98] | 0.08 |
| log.sigma.3.0.mm.3D_firstorder_Range (median [IQR]) | 320.61 [139.68, 447.36] | 345.84 [149.26, 451.67] | 283.95 [100.58, 400.82] | 0.159 |
| log.sigma.3.0.mm.3D_firstorder_RobustMeanAbsoluteDeviation (median [IQR]) | 10.93 [7.41, 18.85] | 10.93 [7.41, 21.83] | 11.08 [7.47, 16.50] | 0.502 |
| log.sigma.3.0.mm.3D_firstorder_RootMeanSquared (median [IQR]) | 46.47 [25.29, 73.86] | 49.04 [23.99, 80.03] | 34.34 [25.98, 62.13] | 0.391 |
| log.sigma.3.0.mm.3D_firstorder_Skewness (median [IQR]) | -1.36 [-2.18, -0.30] | -1.36 [-2.28, -0.37] | -1.43 [-1.97, 0.03] | 0.187 |
| log.sigma.3.0.mm.3D_firstorder_TotalEnergy (median [IQR]) | 71496510.77 [14366114.71, 237433261.58] | 92597604.49 [22487851.42, 315329649.81] | 36862259.27 [10266418.19, 146278641.04] | 0.104 |
| log.sigma.3.0.mm.3D_firstorder_Uniformity (median [IQR]) | 0.36 [0.27, 0.46] | 0.36 [0.24, 0.46] | 0.36 [0.28, 0.46] | 0.7 |
| log.sigma.3.0.mm.3D_firstorder_Variance (median [IQR]) | 1302.02 [207.58, 3179.15] | 1398.69 [225.96, 3816.59] | 834.12 [167.60, 2033.94] | 0.144 |
| log.sigma.3.0.mm.3D_glcm_Autocorrelation (median [IQR]) | 86.52 [13.45, 157.04] | 103.88 [17.91, 174.58] | 61.99 [7.02, 130.05] | 0.095 |
| log.sigma.3.0.mm.3D_glcm_ClusterProminence (median [IQR]) | 368.17 [5.18, 2321.27] | 500.58 [6.79, 2562.96] | 214.26 [3.53, 843.78] | 0.082 |
| log.sigma.3.0.mm.3D_glcm_ClusterShade (median [IQR]) | -29.73 [-138.68, -0.35] | -30.94 [-146.39, -0.55] | -19.96 [-70.62, -0.11] | 0.092 |
| log.sigma.3.0.mm.3D_glcm_ClusterTendency (median [IQR]) | 6.33 [1.22, 17.35] | 7.76 [1.37, 23.29] | 4.86 [1.08, 12.06] | 0.166 |
| log.sigma.3.0.mm.3D_glcm_Contrast (median [IQR]) | 0.63 [0.33, 1.18] | 0.64 [0.34, 1.30] | 0.53 [0.32, 1.08] | 0.461 |
| log.sigma.3.0.mm.3D_glcm_Correlation (median [IQR]) | 0.80 [0.62, 0.86] | 0.81 [0.62, 0.87] | 0.78 [0.62, 0.83] | 0.397 |
| log.sigma.3.0.mm.3D_glcm_DifferenceAverage (median [IQR]) | 0.42 [0.29, 0.60] | 0.41 [0.29, 0.64] | 0.42 [0.31, 0.59] | 0.605 |
| log.sigma.3.0.mm.3D_glcm_DifferenceEntropy (median [IQR]) | 1.12 [0.88, 1.42] | 1.12 [0.91, 1.47] | 1.10 [0.87, 1.39] | 0.509 |
| log.sigma.3.0.mm.3D_glcm_DifferenceVariance (median [IQR]) | 0.42 [0.22, 0.78] | 0.44 [0.22, 0.87] | 0.34 [0.21, 0.63] | 0.373 |
| log.sigma.3.0.mm.3D_glcm_Id (median [IQR]) | 0.82 [0.76, 0.85] | 0.82 [0.75, 0.86] | 0.81 [0.78, 0.85] | 0.809 |
| log.sigma.3.0.mm.3D_glcm_Idm (median [IQR]) | 0.81 [0.74, 0.85] | 0.82 [0.74, 0.85] | 0.80 [0.76, 0.85] | 0.792 |
| log.sigma.3.0.mm.3D_glcm_Idmn (median [IQR]) | 1.00 [0.99, 1.00] | 1.00 [0.99, 1.00] | 1.00 [0.99, 1.00] | 0.422 |
| log.sigma.3.0.mm.3D_glcm_Idn (median [IQR]) | 0.97 [0.96, 0.97] | 0.97 [0.96, 0.97] | 0.97 [0.96, 0.97] | 0.495 |
| log.sigma.3.0.mm.3D_glcm_Imc1 (median [IQR]) | -0.34 [-0.38, -0.28] | -0.34 [-0.38, -0.29] | -0.33 [-0.36, -0.28] | 0.248 |
| log.sigma.3.0.mm.3D_glcm_Imc2 (median [IQR]) | 0.82 [0.68, 0.90] | 0.85 [0.69, 0.90] | 0.80 [0.66, 0.87] | 0.173 |
| log.sigma.3.0.mm.3D_glcm_InverseVariance (median [IQR]) | 0.31 [0.26, 0.34] | 0.31 [0.25, 0.34] | 0.31 [0.28, 0.35] | 0.733 |
| log.sigma.3.0.mm.3D_glcm_JointAverage (median [IQR]) | 9.21 [3.63, 12.27] | 9.94 [4.21, 13.00] | 7.80 [2.61, 11.26] | 0.104 |
| log.sigma.3.0.mm.3D_glcm_JointEnergy (median [IQR]) | 0.22 [0.14, 0.29] | 0.22 [0.14, 0.29] | 0.22 [0.15, 0.27] | 0.974 |
| log.sigma.3.0.mm.3D_glcm_JointEntropy (median [IQR]) | 3.12 [2.36, 4.04] | 3.08 [2.38, 4.13] | 3.21 [2.23, 3.76] | 0.509 |
| log.sigma.3.0.mm.3D_glcm_MCC (median [IQR]) | 0.84 [0.65, 0.89] | 0.85 [0.65, 0.90] | 0.83 [0.64, 0.87] | 0.482 |
| log.sigma.3.0.mm.3D_glcm_MaximumProbability (median [IQR]) | 0.39 [0.28, 0.49] | 0.40 [0.28, 0.49] | 0.38 [0.29, 0.45] | 0.758 |
| log.sigma.3.0.mm.3D_glcm_SumAverage (median [IQR]) | 18.42 [7.27, 24.55] | 19.88 [8.41, 26.00] | 15.61 [5.22, 22.52] | 0.104 |
| log.sigma.3.0.mm.3D_glcm_SumEntropy (median [IQR]) | 2.64 [2.00, 3.19] | 2.64 [2.03, 3.34] | 2.69 [1.88, 3.00] | 0.391 |
| log.sigma.3.0.mm.3D_glcm_SumSquares (median [IQR]) | 1.81 [0.38, 4.65] | 2.13 [0.45, 6.14] | 1.34 [0.35, 3.23] | 0.173 |
| log.sigma.3.0.mm.3D_glrlm_GrayLevelNonUniformity (median [IQR]) | 2379.54 [1347.02, 3849.15] | 2397.43 [1401.32, 4172.97] | 2040.18 [981.51, 3129.00] | 0.356 |
| log.sigma.3.0.mm.3D_glrlm_GrayLevelNonUniformityNormalized (median [IQR]) | 0.24 [0.17, 0.39] | 0.24 [0.17, 0.38] | 0.25 [0.21, 0.43] | 0.271 |
| log.sigma.3.0.mm.3D_glrlm_GrayLevelVariance (median [IQR]) | 2.95 [0.54, 6.99] | 3.38 [0.56, 8.07] | 2.09 [0.42, 4.53] | 0.09 |
| log.sigma.3.0.mm.3D_glrlm_HighGrayLevelRunEmphasis (median [IQR]) | 84.99 [13.81, 144.03] | 98.68 [18.47, 159.71] | 61.61 [7.70, 126.16] | 0.104 |
| log.sigma.3.0.mm.3D_glrlm_LongRunEmphasis (median [IQR]) | 16.66 [10.29, 23.94] | 16.66 [11.51, 24.63] | 15.77 [9.71, 19.45] | 0.66 |
| log.sigma.3.0.mm.3D_glrlm_LongRunHighGrayLevelEmphasis (median [IQR]) | 1105.79 [316.82, 1960.80] | 1344.25 [362.74, 2148.23] | 881.85 [129.87, 1255.79] | 0.049 |
| log.sigma.3.0.mm.3D_glrlm_LongRunLowGrayLevelEmphasis (median [IQR]) | 0.20 [0.08, 2.26] | 0.17 [0.07, 1.77] | 0.31 [0.09, 3.28] | 0.226 |
| log.sigma.3.0.mm.3D_glrlm_LowGrayLevelRunEmphasis (median [IQR]) | 0.02 [0.01, 0.09] | 0.02 [0.01, 0.06] | 0.02 [0.01, 0.17] | 0.191 |
| log.sigma.3.0.mm.3D_glrlm_RunEntropy (median [IQR]) | 4.33 [3.93, 4.75] | 4.40 [3.93, 4.77] | 4.15 [3.78, 4.58] | 0.156 |
| log.sigma.3.0.mm.3D_glrlm_RunLengthNonUniformity (median [IQR]) | 3240.39 [1518.98, 8828.07] | 3418.79 [1543.64, 9754.30] | 2622.19 [1401.60, 5339.15] | 0.21 |
| log.sigma.3.0.mm.3D_glrlm_RunLengthNonUniformityNormalized (median [IQR]) | 0.37 [0.30, 0.45] | 0.36 [0.30, 0.45] | 0.39 [0.30, 0.43] | 0.947 |
| log.sigma.3.0.mm.3D_glrlm_RunPercentage (median [IQR]) | 0.49 [0.41, 0.56] | 0.48 [0.41, 0.55] | 0.50 [0.42, 0.56] | 0.8 |
| log.sigma.3.0.mm.3D_glrlm_RunVariance (median [IQR]) | 7.76 [4.97, 12.19] | 7.98 [5.21, 13.02] | 6.88 [4.55, 11.36] | 0.281 |
| log.sigma.3.0.mm.3D_glrlm_ShortRunEmphasis (median [IQR]) | 0.60 [0.53, 0.67] | 0.59 [0.53, 0.68] | 0.62 [0.53, 0.66] | 0.8 |
| log.sigma.3.0.mm.3D_glrlm_ShortRunHighGrayLevelEmphasis (median [IQR]) | 51.01 [6.98, 95.92] | 57.03 [8.04, 100.99] | 35.21 [4.73, 84.08] | 0.163 |
| log.sigma.3.0.mm.3D_glrlm_ShortRunLowGrayLevelEmphasis (median [IQR]) | 0.01 [0.01, 0.05] | 0.01 [0.01, 0.03] | 0.02 [0.01, 0.09] | 0.191 |
| log.sigma.3.0.mm.3D_glszm_GrayLevelNonUniformity (median [IQR]) | 29.55 [16.26, 60.96] | 31.36 [16.31, 66.86] | 23.64 [13.66, 36.23] | 0.244 |
| log.sigma.3.0.mm.3D_glszm_GrayLevelNonUniformityNormalized (median [IQR]) | 0.15 [0.09, 0.36] | 0.14 [0.09, 0.31] | 0.15 [0.12, 0.40] | 0.202 |
| log.sigma.3.0.mm.3D_glszm_GrayLevelVariance (median [IQR]) | 7.57 [1.52, 11.24] | 7.69 [2.09, 12.14] | 5.41 [0.99, 8.54] | 0.153 |
| log.sigma.3.0.mm.3D_glszm_HighGrayLevelZoneEmphasis (median [IQR]) | 85.88 [21.17, 137.87] | 96.41 [26.79, 137.57] | 70.17 [13.44, 139.00] | 0.317 |
| log.sigma.3.0.mm.3D_glszm_LargeAreaEmphasis (median [IQR]) | 589965.59 [259824.85, 1816974.97] | 591864.55 [259824.85, 2131323.07] | 549423.39 [292060.62, 947982.95] | 0.582 |
| log.sigma.3.0.mm.3D_glszm_LargeAreaHighGrayLevelEmphasis (median [IQR]) | 40007326.65 [10687085.35, 104610461.69] | 42318495.92 [18541721.88, 114485190.15] | 24943717.01 [4419489.75, 84971343.66] | 0.239 |
| log.sigma.3.0.mm.3D_glszm_LargeAreaLowGrayLevelEmphasis (median [IQR]) | 10254.28 [2236.66, 69456.33] | 9043.17 [1677.56, 69456.33] | 10492.55 [3385.54, 74386.86] | 0.636 |
| log.sigma.3.0.mm.3D_glszm_LowGrayLevelZoneEmphasis (median [IQR]) | 0.03 [0.02, 0.09] | 0.03 [0.02, 0.07] | 0.03 [0.02, 0.13] | 0.403 |
| log.sigma.3.0.mm.3D_glszm_SizeZoneNonUniformity (median [IQR]) | 22.14 [7.24, 85.14] | 26.38 [9.46, 111.66] | 15.49 [5.41, 61.29] | 0.169 |
| log.sigma.3.0.mm.3D_glszm_SizeZoneNonUniformityNormalized (median [IQR]) | 0.17 [0.12, 0.20] | 0.17 [0.13, 0.20] | 0.17 [0.11, 0.20] | 0.441 |
| log.sigma.3.0.mm.3D_glszm_SmallAreaEmphasis (median [IQR]) | 0.40 [0.34, 0.45] | 0.41 [0.35, 0.47] | 0.40 [0.31, 0.44] | 0.163 |
| log.sigma.3.0.mm.3D_glszm_SmallAreaHighGrayLevelEmphasis (median [IQR]) | 39.40 [8.31, 66.17] | 45.31 [9.29, 64.08] | 30.67 [4.79, 66.58] | 0.322 |
| log.sigma.3.0.mm.3D_glszm_SmallAreaLowGrayLevelEmphasis (median [IQR]) | 0.01 [0.00, 0.02] | 0.01 [0.00, 0.02] | 0.01 [0.00, 0.02] | 0.904 |
| log.sigma.3.0.mm.3D_glszm_ZoneEntropy (median [IQR]) | 5.80 [4.68, 6.31] | 5.93 [4.84, 6.36] | 5.56 [4.48, 6.16] | 0.231 |
| log.sigma.3.0.mm.3D_glszm_ZonePercentage (median [IQR]) | 0.01 [0.01, 0.02] | 0.01 [0.01, 0.02] | 0.01 [0.01, 0.01] | 0.904 |
| log.sigma.3.0.mm.3D_glszm_ZoneVariance (median [IQR]) | 577083.11 [229381.09, 1585116.24] | 587569.22 [254112.31, 2057208.92] | 480099.07 [211440.51, 822293.32] | 0.226 |
| log.sigma.3.0.mm.3D_ngtdm_Busyness (median [IQR]) | 9.12 [4.87, 29.77] | 9.01 [4.64, 27.12] | 9.47 [6.01, 38.41] | 0.912 |
| log.sigma.3.0.mm.3D_ngtdm_Coarseness (median [IQR]) | 0.00 [0.00, 0.00] | 0.00 [0.00, 0.00] | 0.00 [0.00, 0.00] | 0.15 |
| log.sigma.3.0.mm.3D_ngtdm_Complexity (median [IQR]) | 30.54 [4.07, 72.08] | 37.25 [6.94, 72.08] | 23.22 [2.75, 59.94] | 0.187 |
| log.sigma.3.0.mm.3D_ngtdm_Contrast (median [IQR]) | 0.01 [0.00, 0.01] | 0.01 [0.00, 0.01] | 0.01 [0.01, 0.01] | 0.7 |
| log.sigma.3.0.mm.3D_ngtdm_Strength (median [IQR]) | 0.08 [0.03, 0.13] | 0.08 [0.04, 0.18] | 0.07 [0.01, 0.12] | 0.416 |
| log.sigma.3.0.mm.3D_gldm_DependenceEntropy (median [IQR]) | 6.09 [5.66, 6.64] | 6.09 [5.68, 6.67] | 6.14 [5.65, 6.48] | 0.758 |
| log.sigma.3.0.mm.3D_gldm_DependenceNonUniformity (median [IQR]) | 1047.08 [451.12, 2040.29] | 1198.43 [551.64, 2084.96] | 937.56 [405.45, 1666.97] | 0.214 |
| log.sigma.3.0.mm.3D_gldm_DependenceNonUniformityNormalized (median [IQR]) | 0.05 [0.05, 0.06] | 0.05 [0.05, 0.06] | 0.05 [0.05, 0.06] | 0.306 |
| log.sigma.3.0.mm.3D_gldm_DependenceVariance (median [IQR]) | 42.32 [39.25, 47.81] | 43.58 [40.41, 48.84] | 41.79 [36.52, 45.17] | 0.176 |
| log.sigma.3.0.mm.3D_gldm_GrayLevelNonUniformity (median [IQR]) | 6625.80 [3318.78, 11121.45] | 6947.40 [3432.06, 12405.81] | 5277.20 [2616.84, 9680.71] | 0.291 |
| log.sigma.3.0.mm.3D_gldm_GrayLevelVariance (median [IQR]) | 2.15 [0.41, 5.12] | 2.32 [0.44, 6.15] | 1.41 [0.34, 3.34] | 0.163 |
| log.sigma.3.0.mm.3D_gldm_HighGrayLevelEmphasis (median [IQR]) | 87.66 [13.70, 156.21] | 104.90 [18.06, 174.52] | 62.52 [7.10, 131.54] | 0.09 |
| log.sigma.3.0.mm.3D_gldm_LargeDependenceEmphasis (median [IQR]) | 253.98 [197.41, 314.09] | 255.00 [198.51, 318.45] | 238.16 [196.76, 302.68] | 0.725 |
| log.sigma.3.0.mm.3D_gldm_LargeDependenceHighGrayLevelEmphasis (median [IQR]) | 20035.40 [3791.75, 37909.74] | 22982.03 [6021.76, 39055.23] | 15180.34 [2066.87, 27653.74] | 0.088 |
| log.sigma.3.0.mm.3D_gldm_LargeDependenceLowGrayLevelEmphasis (median [IQR]) | 2.67 [1.19, 25.49] | 2.50 [1.12, 21.99] | 4.70 [1.49, 47.67] | 0.101 |
| log.sigma.3.0.mm.3D_gldm_LowGrayLevelEmphasis (median [IQR]) | 0.02 [0.01, 0.08] | 0.01 [0.01, 0.06] | 0.02 [0.01, 0.17] | 0.159 |
| log.sigma.3.0.mm.3D_gldm_SmallDependenceEmphasis (median [IQR]) | 0.02 [0.01, 0.03] | 0.02 [0.01, 0.03] | 0.02 [0.01, 0.03] | 0.792 |
| log.sigma.3.0.mm.3D_gldm_SmallDependenceHighGrayLevelEmphasis (median [IQR]) | 1.64 [0.18, 4.05] | 1.77 [0.20, 4.27] | 1.08 [0.13, 3.43] | 0.21 |
| log.sigma.3.0.mm.3D_gldm_SmallDependenceLowGrayLevelEmphasis (median [IQR]) | 0.00 [0.00, 0.00] | 0.00 [0.00, 0.00] | 0.00 [0.00, 0.00] | 0.276 |
