# Supplementary material for: A Machine Learning Model for Predicting a Major Response to Neoadjuvant Chemotherapy in Advanced Gastric Cancer
Source: Front Oncol. 2021 Jun 1;11:675458. doi: 10.3389/fonc.2021.675458 (PMC8204104; doi:10.3389/fonc.2021.675458)
Supplement: Supplementary file 5 [file DataSheet_5.docx]

***Procedure of statistical analysis, machine learning algorithm, and R packages utilized in the study***

The normality of data was assessed using the Kolmogorov-Smirnov test and normal probability plots. Parameters that were not normally distributed were expressed in the form of the median (upper quartile to lower quartile) and were analyzed using non-parametric tests: Mann-Whitney test or Kruskal–Wallis test, as appropriate. Normally distributed parameters were expressed in the form of mean ± standard deviation and were analyzed by Student’s t-test. Categorical variables were analyzed by the chi-square test. A p-value<0.05 were identified as statistically significant. Features that achieve statistical significance in univariate analysis were enrolled into the binary logistic regression model for multivariate analysis.

Considering the wide range of radiomic features value, all radiomic feature values were standardized according to their distance to the mean before enrolled into the final analysis. {Formula: (x-mean(x))/standard deviation(x)}. The correlations between the radiomic features and pathological response were firstly selected by univariate analysis. Then, a machine learning algorithm of least absolute shrinkage and selection operator (LASSO) method were used to reduce data dimensionalities, features with a nonzero coefficient were further selected, and radscore was calculated by linearly combining the finally selected coefficients of features.

All statistical analyses were performed using the R software version 3.6.1 (The R Foundation for Statistical Computing, Vienna, Austria; [www.r-project.org](http://www.r-project.org)). The packages of R software used for model training and validation include: “glmnet” package for Lasso binary logistic regression; “rms” package for Mann-Whitney test, Kruskal–Wallis test, Student’s t-test, Chi-square test, multivariate logistic regression, nomograms and calibration plots; “Hmisc” package for calculating the C-index; “rmda” package for decision curve analysis.
