# Supplementary material for: A Machine Learning Model for Predicting a Major Response to Neoadjuvant Chemotherapy in Advanced Gastric Cancer
Source: Front Oncol. 2021 Jun 1;11:675458. doi: 10.3389/fonc.2021.675458 (PMC8204104; doi:10.3389/fonc.2021.675458)
Supplement: Supplementary file 6 [file DataSheet_6.docx]

***Radiomics score (Radscore) calculation formula:***

Radscore = 0.088 × gldm_DependenceNonUniformity - 1.1 × original_shape_LeastAxisLength - 0.12 × original_shape_Maximum3DDiameter - 0.25 × original_firstorder_RootMeanSquared - 0.24 × original_glszm_ZoneVariance

**1. Dependence Non-Uniformity (DN)**


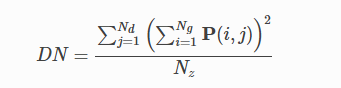


Measures the similarity of dependence throughout the image, with a lower value indicating more homogeneity among dependencies in the image.

**2.Least Axis Length**


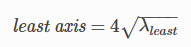


This feature yield the smallest axis length of the ROI-enclosing ellipsoid and is calculated using the largest principal component λleast. In case of a 2D segmentation, this value will be 0. The principal component analysis is performed using the physical coordinates of the voxel centers defining the ROI. It therefore takes spacing into account, but does not make use of the shape mesh.

**3. Maximum 3D diameter**

Maximum 3D diameter is defined as the largest pairwise Euclidean distance between tumor surface mesh vertices. Also known as Feret Diameter.

**4. Root Mean Squared (RMS)**


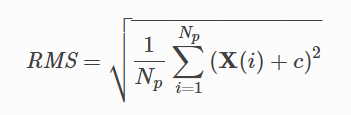


Here, c is optional value, defined by voxelArrayShift, which shifts the intensities to prevent negative values in X. This ensures that voxels with the lowest gray values contribute the least to RMS, instead of voxels with gray level intensity closest to 0. RMS is the square-root of the mean of all the squared intensity values. It is another measure of the magnitude of the image values. This feature is volume-confounded, a larger value of c increases the effect of volume-confounding.

**5. Zone Variance (ZV)**


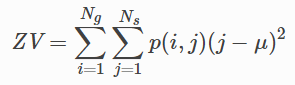

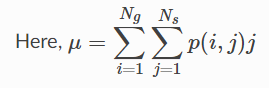


ZV measures the variance in zone size volumes for the zones.

**Reference:**

1. van Griethuysen, J. J. M., Fedorov, A., Parmar, C., Hosny, A., Aucoin, N., Narayan, V., Beets-Tan, R. G. H., Fillon-Robin, J. C., Pieper, S., Aerts, H. J. W. L. (2017). Computational Radiomics System to Decode the Radiographic Phenotype. Cancer Research, 77(21), e104–e107. `https://doi.org/10.1158/0008-5472.CAN-17-0339 <https://doi.org/10.1158/0008-5472.CAN-17-0339>
